# Donafenib and sintilimab combined with hepatic arterial infusion chemotherapy for unresectable hepatocellular carcinoma: a prospective, single-arm phase II trial (DoHAICs study)

**DOI:** 10.1016/j.eclinm.2025.103217

**Published:** 2025-05-05

**Authors:** Wei Gao, Zhao-Long Pan, Xiao-Hui Zhao, Lu Yang, Jun-Bo Cao, Dong-Yang Li, Hai-Jing Zheng, Chen Liu, Guang-Tao Li, Xu Bao, Xiao-Meng Liu, Wei-Hao Zhang, Xiao-Lin Zhu, Bo-Han Xiao, Tian-Qiang Song, Qiang Li, Wei Lu, Wen-Ge Xing, Wei Zhang

**Affiliations:** a Department of Interventional Therapy, Tianjin Medical University Cancer Institute & Hospital, National Clinical Research Center for Cancer, Tianjin, China; b Department of Hepatobiliary Surgery, Liver Cancer Prevention and Treatment Research Center, Tianjin Medical University Cancer Institute & Hospital, Tianjin, China; c Department of Radiology, Liver Cancer Center, Tianjin Medical University Cancer Institute and Hospital, National Clinical Research Center for Cancer, Key Laboratory of Cancer Prevention and Therapy, Tianjin's Clinical Research Center for Cancer, Tianjin Medical University, Tianjin, China; d Department of Hepatobiliary Oncology, Liver Cancer Prevention and Treatment Research Center, Tianjin Medical University Cancer Institute & Hospital, Tianjin, China; e National Clinical Research Center for Cancer, Tianjin, China; f Tianjin’s Clinical Research Center for Cancer, Tianjin, China; g Tianjin Key Laboratory of Digestive Cancer, Tianjin, China; h Key Laboratory of Cancer Prevention and Therapy, Tianjin, China

**Keywords:** Hepatocellular carcinoma, Hepatic arterial infusion chemotherapy, Conversion therapy, Surgical resection, Immune checkpoint inhibitors

## Abstract

**Background:**

Donafenib demonstrates superior survival benefits and safety compared to sorafenib. Hepatic arterial infusion chemotherapy (HAIC) proves effective for large unresectable hepatocellular carcinoma (uHCC) over transarterial chemoembolization. This study aims to investigate the safety and efficacy of combining HAIC with donafenib and sintilimab in patients with uHCC.

**Methods:**

This prospective single-arm study enrolled patients with histologically confirmed uHCC and no prior systemic treatment from December 2021 to May 2023 in Tianjin Medical University Cancer Institute and Hospital, China. Participants received donafenib, sintilimab and HAIC until disease progression or unacceptable toxicity. The primary endpoint was the objective response rate (ORR). The secondary endpoints included overall survival (OS), event-free survival (EFS), disease control rate (DCR) and safety. The study was registered at ClinicalTrials.gov, NCT05166772.

**Findings:**

A total of 36 patients were enrolled, and the median follow-up was 16 months. ORR were 58.3% and 80.6% per RECIST 1.1 and mRECIST respectively, and DCR were all 94.4%. 24-month OS rate was 59.6%. Median EFS was 15.3 months. The conversion success rate was 50.0%, 18 patients underwent hepatectomy, seven (38.9%) achieved pathological complete response, and nine (50.0%) achieved major pathological responses. Median OS was not achieved (NA) in the surgical group vs. 14.3 months (95% CI 12.1–NA) in the non-surgical group (*p* = 0.035). Adverse reactions were manageable.

**Interpretation:**

Combining HAIC with donafenib and sintilimab demonstrates promising clinical outcomes while maintaining acceptable toxicity, making it a potential strategy for conversion therapy.

**Funding:**

Tianjin Key Medical Discipline (Specialty) Construction Project (TJYXZDXK-009A) and Precision Treatment Project of Surgical Oncology of Tianjin Medical University Cancer Institute and Hospital (No. ZLWKJZZL14).

**Research in context**

**Evidence before this study**

Hepatocellular carcinoma (HCC) is often diagnosed at advanced stages, conversion therapies combining hepatic arterial infusion chemotherapy (HAIC) and systemic treatments are emerging as promising approaches to transform unresectable HCC into resectable disease, but their efficacy and safety require further investigation. We searched PubMed, with no language restrictions, for studies published from the inception of the database until December 25, 2024, using the search terms (“unresectable” AND “hepatocellular carcinoma” AND “hepatic arterial infusion chemotherapy” AND “tyrosine kinase inhibitor” AND “immune checkpoint inhibitor”). We found retrospective studies, meta-analyses and case report, but no prospective trials investigating the efficacy and safety of HAIC combined with donafenib and sintilimab for unresectable HCC.

**Added value of this study**

This study demonstrates that the combination of HAIC, donafenib, and sintilimab resulted in a favourable objective response rate and enabled a significant proportion of patients with unresectable HCC (N = 18/36) to undergo successful surgical resection.

**Implications of all the available evidence**

The findings suggest that integrating HAIC, donafenib, and sintilimab into clinical practice could improve outcomes for patients with advanced HCC, supporting further research and potential changes in treatment protocols to include combination immune-based therapies for advanced-stage cancers.

## Introduction

In China, hepatocellular carcinoma (HCC) ranks fourth in incidence^1^ and is the second leading cause of cancer-related death.^2^^,^^3^ Although the incidence and mortality rates of HCC are decreasing,^4^ the overall life expectancy of patients with HCC remains low. A significant contributing factor is that most HCC cases are diagnosed at an advanced stage, thereby missing the opportunity for radical resection.^5^ According to the China Liver Cancer Staging (CNLC) system, patients with early and some intermediate HCC (mainly patients with stage Ia, Ib, and some stage IIa) are candidates for radical treatment, such as surgical resection, local ablation, and liver transplantation, which offers a median survival time exceeding 5 years.^6^^,^^7^ Unfortunately, the majority of patients with HCC are diagnosed at middle and late stages (stages IIb, IIIa, and IIIb), which correspond to advanced stages in the Barcelona Clinic Liver Cancer (BCLC) system (including some BCLC-B patients and all BCLC-C patients). A small proportion of patients with advanced HCC can undergo surgical resection, which may be superior to nonsurgical treatment. However, short-term recurrence rates after surgery are very high. For most patients with advanced HCC, local and/or systemic therapy is the primary treatment option.^8^^,^^9^

Systemic therapies of anti-angiogenesis combined with immune checkpoint inhibitors has achieved rapid development in recent years, such combined therapy has shown the treatment benefits in multiple phase III studies^10^, ^11^, ^12^ and has become the standard treatment for unresectable hepatocellular carcinoma (uHCC). Emerald-1 and LEAP-012 study demonstrated that TACE combined with systemic therapies acquired better ORR and PFS than TACE monotherapy in intermediate uHCC,^13^^,^^14^ and a multicenter, retrospective cohort study showed comprehensive benefits of TACE combined with systemic therapies compared to systemic therapies in advanced uHCC initially.^15^ Local treatment combined with systemic treatment has been a new direction for therapeutic development.

Hepatic arterial infusion chemotherapy (HAIC) has recently emerged as a significant local treatment for advanced HCC. A multicenter randomized controlled trial showed that HAIC had a significantly higher objective response rate (ORR) and survial benefit than TACE in the uHCC patient with large tumor.^16^ Another multicenter randomized controlled trial demonstrated that HAIC achieved better benefits than sorafenib in uHCC patients with portal vein tumor thrombosis (PVTT).^17^ A similar study comparing HAIC combined with sorafenib vs. sorafenib monotherapy in uHCC patients with PVTT found that 12.8% of patients in the combination group achieved regression and underwent radical surgical resection, with three patients achieving a pathological complete response (pCR).^18^ These findings suggest that HAIC-based local therapy combined with systemic therapy can achieve higher antitumor activity, thereby enabling more patients with advanced disease to undergo resection. Five-year survival rates of 50–60% can be achieved in patients undergoing “conversion and resection”, comparable to the survival rates following resection of early-stage HCC, thus offering the possibility of long-term survival.^19^^,^^20^

Oriental-32 study reported that Sintilimab combined with bevacizumab showed good efficacy and safety in uHCC patients.^11^ Furthermore, the ZGDH3 study indicated that donafenib was superior to sorafenib in improving overall survival (OS), making it more suitable for Chinese patients.^21^ Sintilimab combined with bevacizumab and donafenib is recommended as first-line systemic drugs in the “Guideline for the Diagnosis and Treatment of Primary Liver Cancer (2024 edition)”.^22^ However, the effects of combining donafenib and sintilimab have not been reported.

Therefore, this study prospectively enrolled 36 consecutive patients with uHCC, utilizing a strategy of HAIC combined with donafenib and sintilimab, followed by R0 resection. The aim was to assess the efficacy and safety of this strategy, as well as to analyze the potential survival benefit for patients undergoing radical surgery after successful conversion therapy.

## Methods

### Study design and participants

This was a single-center, single-arm, phase II study evaluating the combination therapy of donafenib and sintilimab with HAIC to treat uHCC. Eligible participants, aged 18–80 years old, were clinically diagnosed with unresectable or metastatic HCC according to the Guidelines for Diagnosis and Treatment of Primary Liver Cancer in China (2019 edition)^21^ or confirmed by histology/cytology. Inclusion criteria encompassed Eastern Cooperative Oncology Group performance status (ECOG PS) 0–1, Child-Pugh score ≤7, and at least one measurable target lesion assessed per Response Evaluation Criteria in Solid Tumors, Version 1.1 (RECIST 1.1). Exclusion criteria included: 1) prior systemic therapy (e.g., sorafenib, lenvatinib, and regorafenib), targeted therapies against vascular endothelial growth factor/vascular endothelial growth factor receptor (VEGF/VEGFR), rapidly accelerated fibrosarcoma (RAF), and mitogen-activated protein kinase (MEK) signaling pathways, or immune modulators (e.g., anti-PD-1, anti-programmed cell death-ligand 1 (anti-PD-L1), or anti-cytotoxic T lymphocyte-associated antigen-4 (anti-CTLA-4) agents); 2) tumor vascular invasion presents one or more of the following conditions: a) Involvement of the superior mesenteric vein; b) Involvement of the inferior vena cava.

### Ethics

This study adhered to international clinical trial standards and the Declaration of Helsinki. Written informed consent was obtained from all participants before enrollment. The study protocol was approved by the Ethics Committee of Tianjin Medical University Cancer Institute and Hospital (E20210872). This study was registered at ClinicalTrials. gov (NCT05166772).

### Procedures

Enrolled patients received donafenib (200 mg twice daily, taken orally, initiated 3–7 days before the first HAIC session), sintilimab (200 mg intravenously every 3 weeks, administered 0–1 day before each HAIC treatment), and HAIC (oxaliplatin 85 mg/m^2^ over 2 h, leucovorin 400 mg/m^2^ over 2 h, bolus fluorouracil 400 mg/m^2^ within the first 10 min, followed by fluorouracil infusion 1200 mg/m^2^ over 23 h, every 3 weeks. The dose of oxaliplatin can be adjusted based on tumor size and vascularity. For tumors with a diameter greater than 10 cm and abundant blood supply, a dose of 130 mg/m2 of oxaliplatin is used. For tumors with a diameter of ≤10 cm and less abundant blood supply, a dose of 85 mg/m2 of oxaliplatin is selected. Additionally, if a large tumor significantly shrinks after several courses of HAIC, the dose can be appropriately reduced, even to 60 mg/m^2^. Subsequent dosage adjustments can be made based on the actual treatment response and patient tolerability.) until disease progression, unacceptable toxicity, or patient withdrawal.

### Outcomes and assessments

The primary endpoint was the ORR, defined as the percentage of patients with an overall best response of complete response (CR) or partial response (PR) based on RECIST 1.1 and modified RECIST (mRECIST). RECIST 1.1 was used as the primary standard for assessment. Secondary endpoints included: 1) OS, defined as time from initiation of combination therapy to death. If the participants were still alive at the end of the database enrollment period, they were censored at the last date of follow-up in the database; 2) event-free survival (EFS), time from initiation of combination therapy to progression, recurrence, or death. For participants lost to follow-up without confirmation of radiological progression, recurrence, or death, they were considered as censored, and the censoring time was defined as the last follow-up date where no evidence of progression or recurrence was found. For surviving participants with no confirmed tumor progression or recurrence at the end of the database enrollment period, they were censored based on the last imaging tumor assessment date; 3) disease control rate (DCR), percentage of patients with an overall best response of CR, PR, or stable disease (SD), 4) duration of response (DoR), time from the first evidence of CR or PR to disease progression or death, 5) time to response (TTR), time from initiation of study treatment to the first evidence of CR or PR), 6) changes in alpha-fetoprotein (AFP) levels and safety assessed using the Common Terminology Criteria for Adverse Events (CTCAE) V.5.0.

Tumors were assessed using dynamic contrast-enhanced computed tomography (CT) or magnetic resonance imaging (MRI) according to mRECIST criteria within 14 days before the first dose. Post-baseline measurements were conducted every 6 weeks until progression, intolerable toxicity, withdrawal of consent, or trial conclusion.

### Statistics

This study is an exploratory study with a fixed sample size of 30 cases. Statistical analyses were performed using R version 4.3.1. Efficacy and safety were analyzed in all patients who received at least one dose of the study treatment. Categorical variables were presented as frequencies and percentages and compared using Pearson’s χ2 or Fisher’s exact tests. Continuous variables were presented as median with range or mean with standard deviation and compared using the t-test or Mann–Whitney U test. Paired values were compared using paired t-test or Wilcoxon matched-pair signed-rank test. Multiple comparison errors were controlled using Bonferroni correction.

The ORR and DCR were summarized as binomial response rates, with the corresponding two-sided 95% exact confidence intervals (CIs) calculated using the Clopper-Pearson method. Survival analyses were conducted using the Kaplan–Meier method, with differences analyzed using the log-rank test. All variables with *p* < 0.2 during univariate analyses were included in multivariate analyses, which included a Cox regression analysis to identify factors independently associated with EFS and OS. The proportional hazards (PH) assumption was verified using Schoenfeld residuals and global tests. *p* values were two-sided, with a significance level 0.05 for all analyses.

### Role of the funding source

The funders of the study had no role in study design, data collection, data analysis, data interpretation, or writing of the report.

## Results

### Patient characteristics

**Fig. 1:**
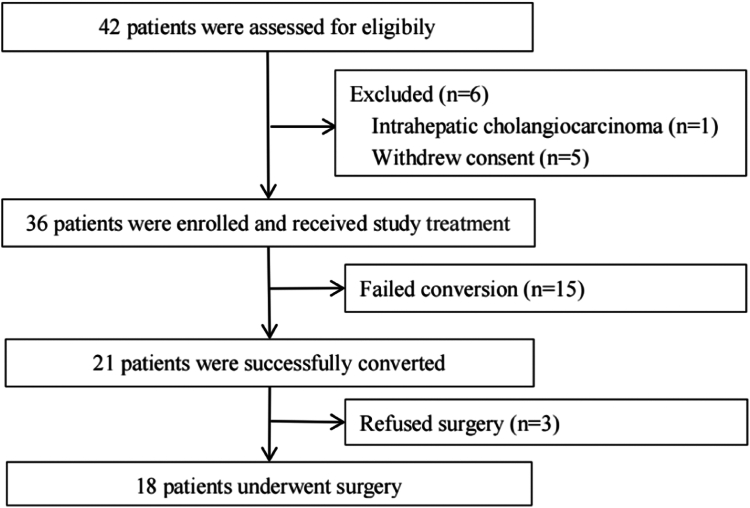
**Study flow diagram of patients**.

Thirty-six eligible patients were enrolled between December 2021 and May 2023 (Fig. 1). Baseline characteristics are presented in Table 1. BCLC stage C was observed in 55.6% of patients, with the majority having an ECOG PS of 0 and Child-Pugh class A. The median maximum tumor diameter was 81.5 mm. Twelve patients (33.4%) had PVTT of Vp 3 or 4, and three (8.3%) had extrahepatic metastasis. Seventeen patients (47.2%) had liver cirrhosis.

**Table 1:** Baseline characteristics.

Characteristic	Overall (n = 36)	Characteristic	Overall (n = 36)
Age, mean (SD), years	58.8 (9.3)	IIIb	3 (8.3)
Sex, n (%)		Extrahepatic disease, n (%)	
Male	30 (83.3)	No	33 (91.7)
Female	6 (16.7)	Yes	3 (8.3)
ECOG PS, n (%)		Macrovascular invasion, n (%)	
0	35 (97.2)	No	18 (50.0)
1	1 (2.8)	Yes	18 (50.0)
HBV infection, n (%)		PVTT, n (%)	
Yes	29 (80.6)	Vp1	1 (2.8)
No	7 (19.4)	Vp2	1 (2.8)
Cirrhosis, n (%)		Vp3	11 (30.6)
Yes	17 (47.2)	VP4	1 (2.8)
No	19 (52.8)	IVCTT, n (%)	3 (8.3)
AFP, n (%), ng/mL		HVTT, n (%)	7 (19.4)
<400	21 (58.3)	Maximum diameter of tumor, median (range), mm	81.5 (33.3, 193.0)
≥400	15 (41.7)	Drinking history, n (%)	
BCLC stage, n (%)		No	15 (41.7)
A	4 (11.1)	Yes	21 (58.3)
B	12 (33.3)	History of treatment, n (%)	
C	20 (55.6)	No	33 (91.7)
CNLC stage, n (%)		Yes^a^	3 (8.3)
Ib	4 (11.1)	Child-Pugh class (%)	
IIa	6 (16.7)	A	35 (97.2)
IIb	6 (16.7)	B	1 (2.8)
IIIa	17 (47.2)		

AFP, Alpha-fetoprotein; BCLC, Barcelona Clinic Liver Cancer; CNLC, China liver cancer staging; ECOG PS, Eastern Cooperative Oncology Group Performance Status; HBV, Hepatitis B Virus; HVTT, Hepatic vein tumor thrombus; IVCTT, Inferior vena cava tumor thrombus; PVTT, portal vein tumor thrombus.

The sample size is 36.

a One patient had a history of R0 liver resection, one had a history of ablation, and one had a history of transcatheter arterial embolization.

### Efficacy

**Fig. 2:**
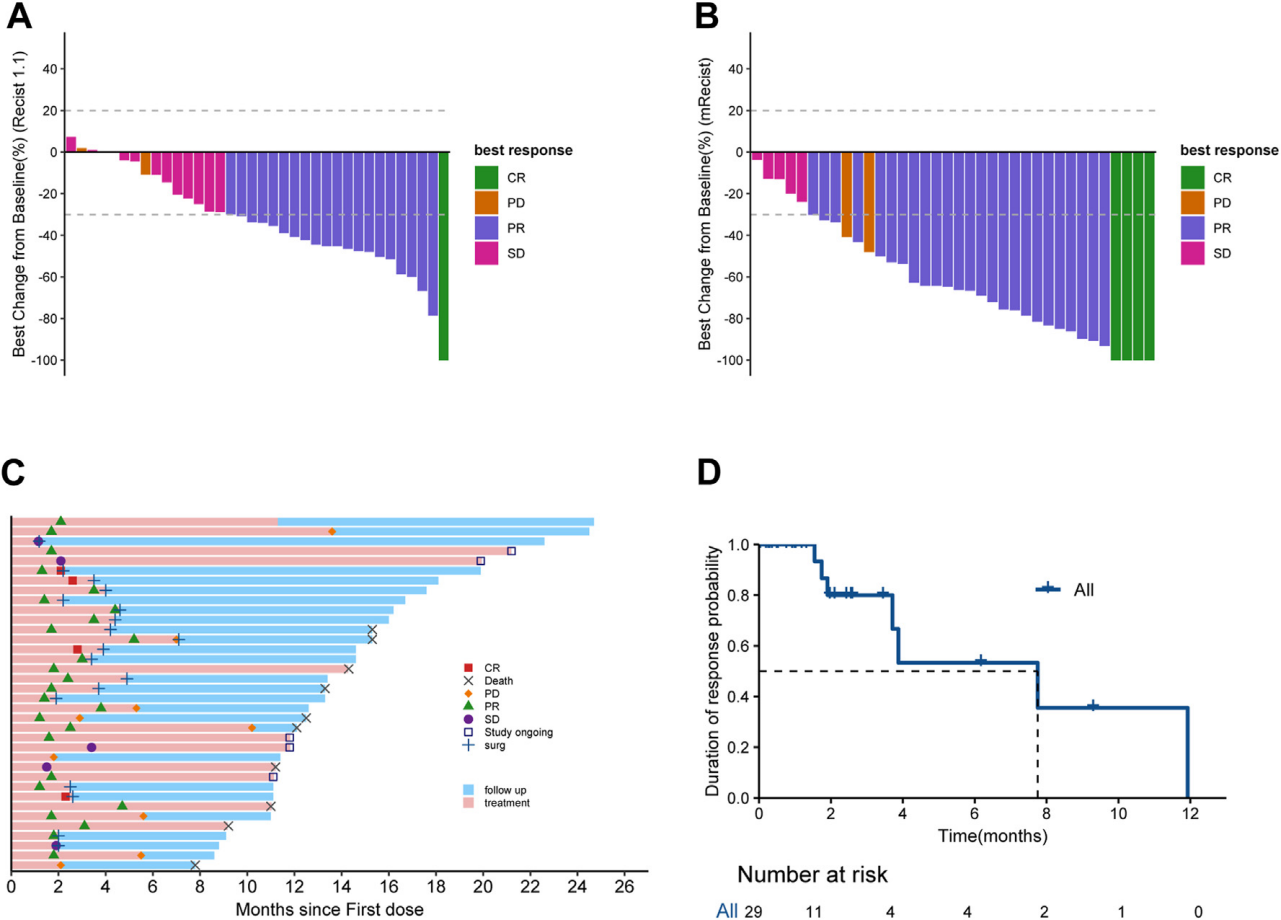
**Response of 36 patients with HCC to the combined therapy of donafenib plus sintilimab with HAIC.** (A) Waterfall plot of the best percentage change in target-lesion size from baseline according to RECIST 1.1. (B) Waterfall plot of the best percentage change in target-lesion size from baseline according to mRECIST. (C) A swimmer plot demonstrating the clinical courses of study participants. (D) Kaplan–Meier curves of duration of response. HAIC, hepatic arterial infusion chemotherapy; HCC, Hepatocellular carcinoma; mRECIST, modified Response Evaluation Criteria in Solid Tumors; RECIST 1.1, Response Evaluation Criteria in Solid Tumors V.1.1.

As of January 26, 2024, the median follow-up duration was 16 months (95% CI 13.3–19.9). The median number of HAIC was three. According to the RECIST 1.1, one patients achieved CR (2.8%, 95% CI 0.1–14.5), 20 achieved PR (55.5, 95% CI 38.1–72.1), resulting in an ORR of 58.3% (95% CI 40.8–74.5) and a DCR of 94.4% (95% CI 81.3–99.3). According to the mRECIST, four patients achieved CR (11.1%, 95% CI 3.1–26.1), and 25 achieved PR (69.4%, 95% CI 51.9–83.7), resulting in an ORR of 80.6% (95% CI 64.0–91.8) and a DCR of 94.4% (95% CI 81.3–99.3) (Online Supplemental Table S1, Fig. 2A and B). Most patients showed a decrease in target lesion size at the first tumor assessment (Online Supplemental Fig. S1). The median TTR was 1.8 (range:1.2–5.2) months, and the median DoR was 7.8 months (95% CI 3.71–not achieved [NA]) per mRECIST (Fig. 2D). Subgroup analysis of the ORR was consistent across all subgroups, with ORR exceeding 57.1% in all subgroups and reaching 91.7% in patients with PVTT of Vp3 and Vp4 (Online Supplemental Fig. S2). Furthermore, when comparing the baseline characteristics of non-responders to therapy with responders, it was found that non-responders had a lower proportion of HBV infection rate (57.1% vs. 86.2%, *p* = 0.226) in comparison to responders (Online Supplemental Table S2).

Among the 36 patients, 21 patients met the criteria for surgical resection after receiving conversion therapy, defined as a) no extrahepatic metastasis; b) sufficient remaining liver volume; c) regressed or inactive large vessel tumor thrombosis; and d) adequate surgical margins. Among them, 4 patients achieved CR, 15 patients achieved PR, and 2 patients had SD. Eighteen patients underwent surgical resection, all achieving R0 resection. The conversion success rate was 50.0% (95% CI 32.9–67.1). The median time from treatment initiation to surgery was 105 days (range: 36–215 days) (Fig. 2C). Postoperative pathology showed a pCR (defined as 100% necrosis) in seven patients (38.9%) and a major pathological response (MPR, defined as 70% ≤ necrosis <100%) in nine patients (50%). Baseline characteristics of patients who underwent surgery vs. those who did not are detailed in Online Supplemental Table S2. These results were obtained through post-hoc analyses.

**Fig. 3:**
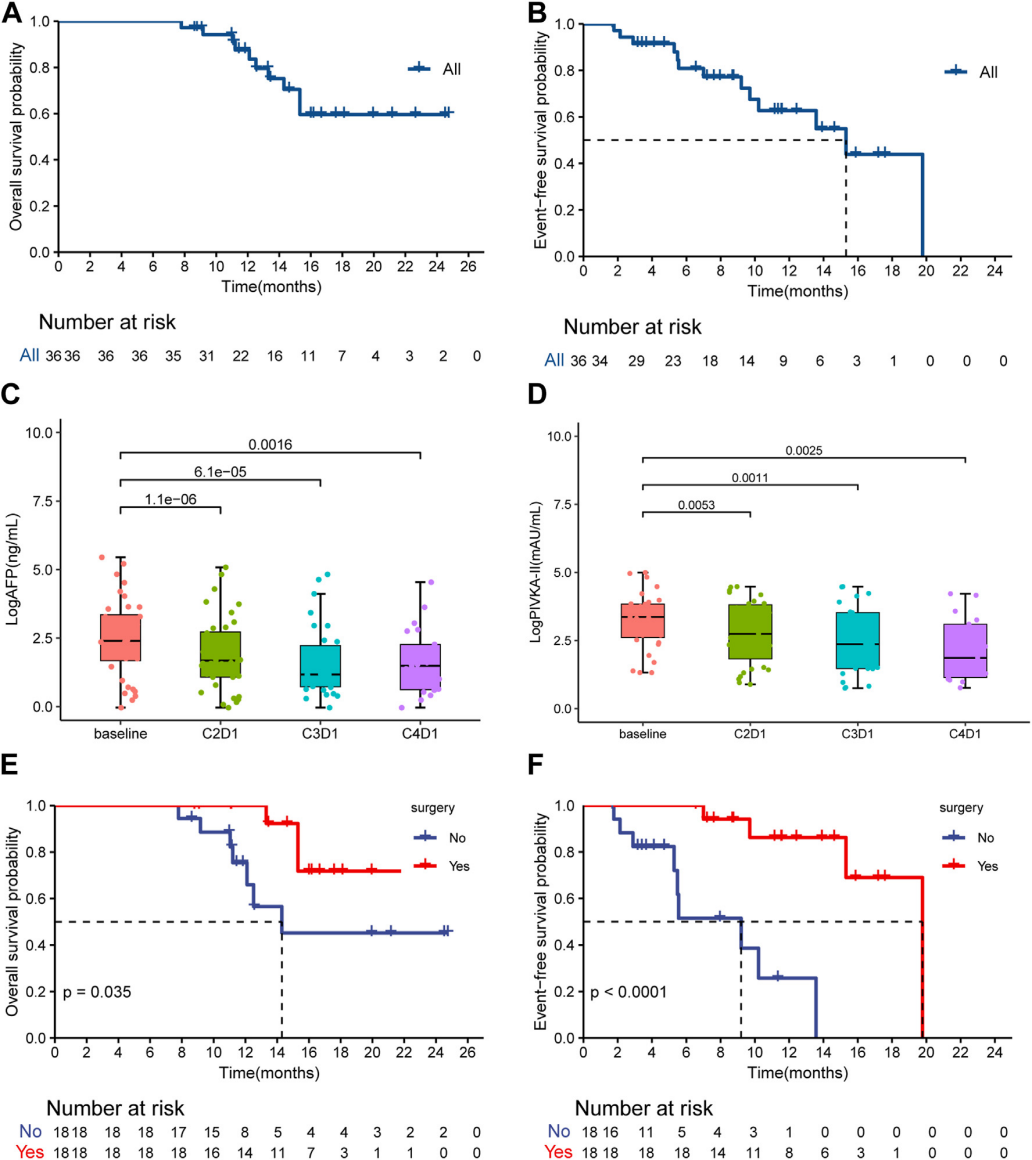
**Kaplan–Meier curves of event-free survival (EFS) and overall survival (OS) among the enrolled patients and the change of AFP and PIVKA-II.** (A) Kaplan–Meier curves of OS among 36 patients. (B) Kaplan–Meier curves of EFS among 36 patients. (C) The change of AFP from baseline to during treatment (C2D1, C3D1, C4D1). (D) The change of PIVKA-II from baseline to during treatment. (E) Kaplan–Meier curves of OS among patients stratified by surgery. (F) Kaplan–Meier curves of EFS among patients stratified by surgery. EFS was assessed according to mRECIST. (C) Used Wilcoxon matched-pair signed-rank test to compare the difference between baseline and CND1 with sample sizes of n = 36 each. (D) Used paired t-test to compare the difference between baseline and CND1 with sample sizes of n = 36 each. (E) and (F) Used the log-rank test to compare the difference between two groups with sample sizes of n = 18 each. AFP, Alpha-fetoprotein; C2D1, Cycle (C) 2 Day (D) 1; C3D1, Cycle (C) 3 Day (D) 1; C4D1, Cycle (C) 4 Day (D) 1; mRECIST, modified Response Evaluation Criteria in Solid Tumors; PIVKA-II, Proteins induced by vitamin K absence or antagonist-II.

The median OS was not achieved for the 36 patients who received combination therapy, with a 24-month OS rate of 59.6% (95% CI 42.4–83.9) (Fig. 3A). The median EFS was 15.3 months (95% CI 10.2–NA), with EFS rates of 80.9% and 62.7% at 6 and 12 months, respectively (Fig. 3B). Significant reductions were observed in AFP levels and proteins induced by vitamin K absence or antagonist-II (PIVKA) (Online Supplemental Tables S4 and S5, Fig. 3C and D).

At the time of data cutoff, 83.3% of patients in the surgical group were still alive and under continuous follow-up. The median OS was not achieved in the surgical group, whereas it was 14.3 months (95% CI 12.1–NA) in the non-surgical group, showing a statistically significant difference (*p* = 0.035) (Fig. 3E). The surgical group also had significantly longer EFS (median EFS, 19.8 vs. 9.2 months, *p* < 0.001) compared to the non-surgical group (Fig. 3F). The PH assumption was satisfied for all covariates (Online Supplemental Tables S6 and S7), and visual inspection of Schoenfeld residual plots (Online Supplemental Figs. S3 and S4) confirmed no systematic deviation from proportionality over time. Cox regression analysis indicated that surgery demonstrated a significant association with better EFS (hazard ratio (HR) 0.10, 95% CI 0.02–0.51, *p* = 0.006) and OS (HR 0.24, 95% CI 0.06–0.97, *p* = 0.046) (Online Supplemental Tables S8 and S9). However, surgery isn’t a traditional prognostic factor at baseline, it’s a variable related to treatment.

**Fig. 4:**
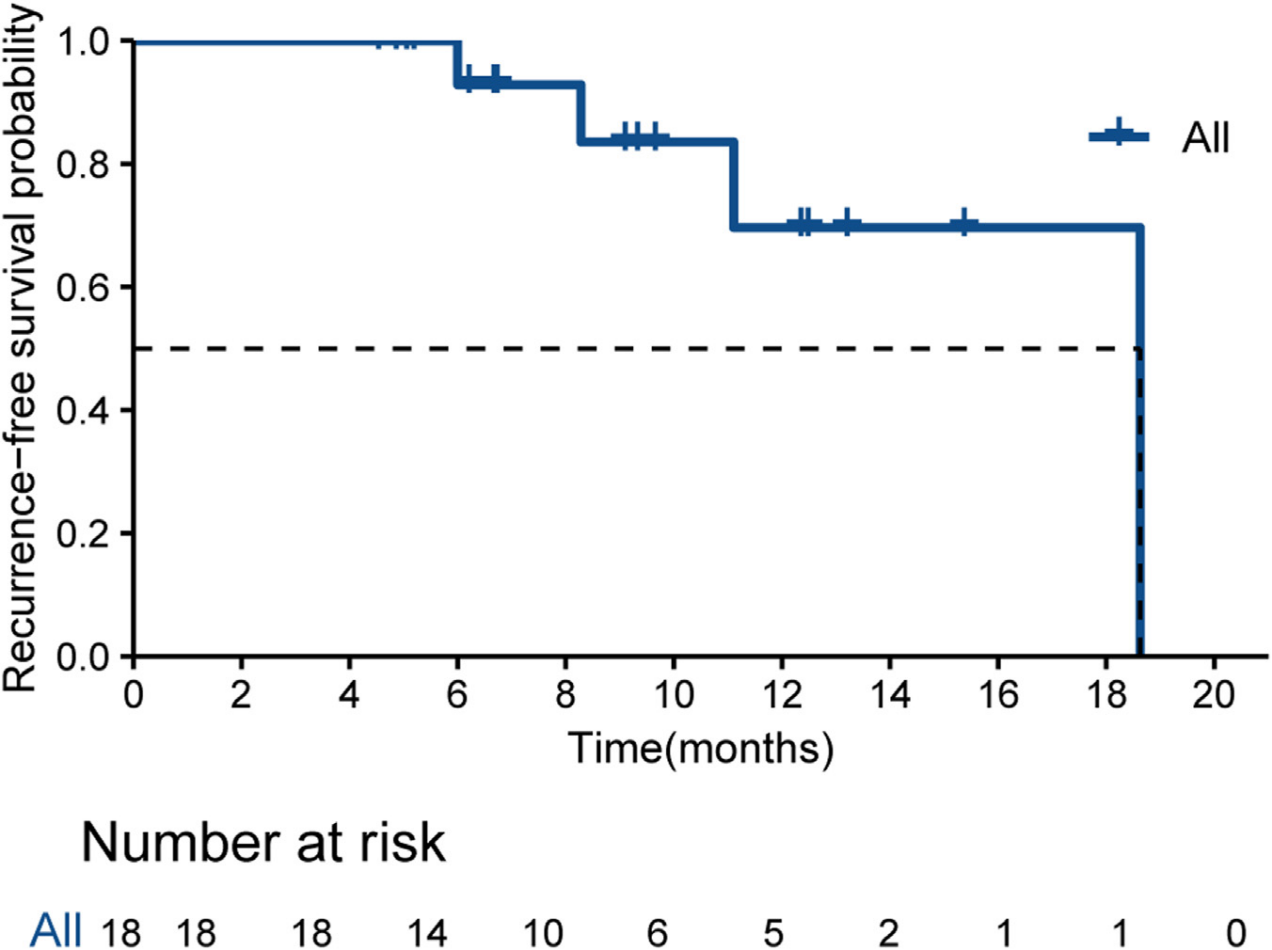
**Kaplan–Meier analysis of recurrence-free survival among 18 patients reveived surgery**.

Patients continued to receive combination therapy of donafenib and sintilimab after surgery. Currently, four patients have experienced recurrence or death postoperatively. All four cases were intrahepatic recurrences. The median recurrence-free survival (RFS) in the surgical group was 18.6 months (95% CI, 11.1–NA). The 12-month RFS rates in the surgical group were 69.6% (95% CI 44.9%-100.0%) (Fig. 4). These results were obtained through post-hoc analyses.

### Safety

Among 36 patients who received at least one dose of combination therapy, treatment-emergent adverse events (TEAEs) of any grade occurred in 35 (97.2%) patients. The most common TEAEs were thrombocytopenia (n = 25, 69.4%), increased alanine aminotransferase (ALT) (n = 17, 47.2%), increased aspartate aminotransferase (AST) (n = 16, 44.4%), and anemia (n = 15, 41.7%). Twelve (33.3%) patients reported grade 3 or 4 TEAEs, including 4 cases of rash, 4 cases of increased ALT, 4 cases of increased AST, 2 cases of hand-foot syndrome, 1 case of thrombocytopenia, and 1 case of increased blood bilirubin. No treatment-related deaths occurred during the study period (Table 2).

**Table 2:** Treatment-emergent adverse events occurring in >3% of patients by CTCAE grade.

	Any grade n (%)	Grade ≥3 n (%)
Patients with any AE	35 (97.2)	12 (33.3)
Thrombocytopenia	25 (69.4)	1 (2.8)
Alanine aminotransferase increased	17 (47.2)	4 (11.1)
Aspartate aminotransferase increased	16 (44.4)	4 (11.1)
Anemia	15 (41.7)	
Neutropenia	12 (33.3)	
Alkaline phosphatase increased	11 (30.6)	
Hand-foot syndrome	10 (27.8)	2 (5.6)
Rash	10 (27.8)	4 (11.1)
White blood cell count decreased	8 (22.2)	
Blood bilirubin increased	8 (22.2)	1 (2.8)
Diarrhea	7 (19.4)	
Fever	4 (11.1)	
Increased hemoglobin	2 (5.6)	
Anorexia	2 (5.6)	

AE, adverse event; CTCAE, Common Terminology Criteria for Adverse Event. The sample size is 36.

### Typical case

**Fig. 5:**
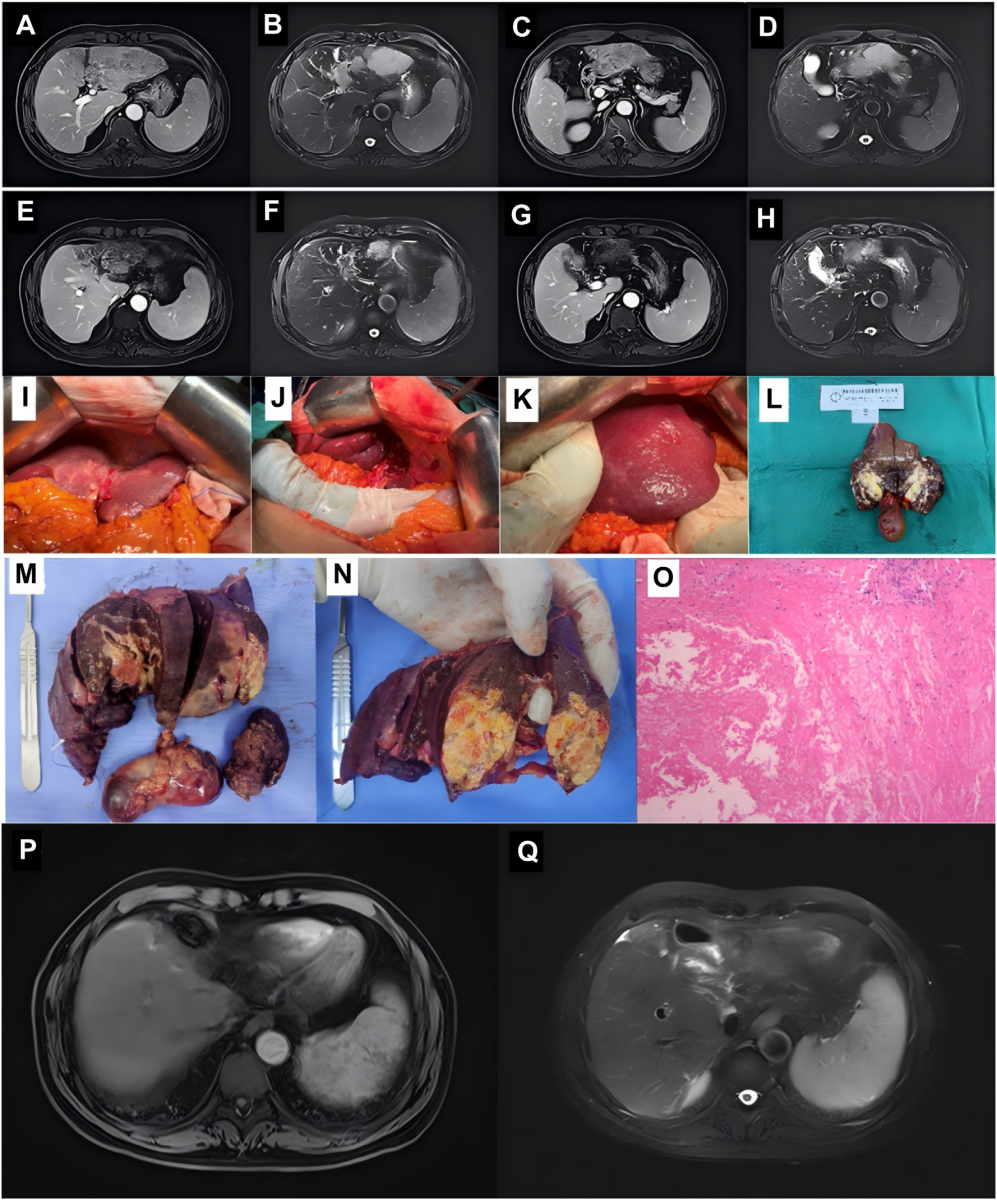
**Typical case.** (A–D) Pre-treatment MRI results of patients. (E–H) MRI results of patients after 2 cycles of treatment. (I–N) Surgical resection specimens. (O) Postoperative pathology of patients. (P and Q) Postoperative follow-up MRI results. MRI, magnetic resonance imaging.

The patient was admitted with the chief complaint of “liver mass detected for over one week.” Abdominal MRI plain scan and contrast-enhanced imaging revealed: a mass in the left lobe of the liver, approximately 6.4 × 10.1 cm, accompanied by cancer thrombus in the right branch of the portal vein (Fig. 5A–D). Biopsy: (Left lobe liver biopsy) Malignant tumor, combined with immunohistochemistry results, consistent with poorly differentiated carcinoma, tending toward hepatocellular carcinoma. The patient was classified as BCLC stage C. Two cycles of combined treatment with donafenib, sintilimab, and HAIC were administered. Follow-up MRI showed a significant reduction in the size of the lesion in the left lobe of the liver and the thrombus in the left branch of the portal vein, with reduced enhancement (Fig. 5E–H). According to mRECIST criteria, this indicated a PR. Surgery was performed for resection (Fig. 5I-N). Postoperative pathology: (Left hemihepatectomy + left caudate lobe) No definitive residual cancer tissue was observed in all sections, with large areas of necrosis and fibrosis, consistent with pCR (Fig. 5O). Postoperative follow-up: Signal in the residual liver surgical area was uneven, and a long T1 and long T2 signal shadow was observed around the surgical area, with no enhancement detected (Fig. 5P, Q). The patient consented to the utilization of the information, and the informed consent form has been placed in the online Supplemental.

## Discussion

This phase II study demonstrated that combining donafenib and sintilimab with HAIC effectively treats uHCC. Eighteen patients underwent surgery, effectively converting those with unresectable advanced HCC into resectable cases. This suggests that surgical resection following successful conversion therapy provides the potential for long-term survival and that the regimen is well tolerated.

HAIC monotherapy achieves an ORR of 20%–40% in patients with advance HCC and shows survival benefit, especially for the patients with portal vein invasion or large tumors, primarily through enhanced local control.^16^^,^^17^^,^^23^ Systemic therapies of anti-angiogenesis combined with immune checkpoint inhibitors has gotten rapid development in recent years, showed ORR of over 20% and OS of around 20 months.^10^, ^11^, ^12^ Combination strategies (e.g., HAIC + TKIs) have shown ORRs of 40%, suggesting additive effects.^18^^,^^24^ Notably, our regimen (HAIC + donafenib + sintilimab) achieved an ORR of 58.3% (RECIST 1.1) and 80.6% (mRECIST), with 50% of patients undergoing conversion surgery. These results surpass historical data for both HAIC monotherapy and systemic therapies, supporting the hypothesis that combining localized and systemic modalities may overcome limitations of single approaches. HAIC may enhance tumor immunogenicity via rapid debulking, potentially sensitizing tumors to anti-PD-1 agents, while TKIs like donafenib could suppress compensatory angiogenesis post-HAIC. As one of the studies to integrate HAIC with TKIs and PD-1 inhibitors, our data provide a foundation for phase III trials comparing this combination against standard therapies.

In this study, we observed seven patients who did not respond to treatment. Baseline analysis revealed that non-responders had a lower HBV infection rate (57.1% vs. 86.2%) compared to responders. However, due to the small sample size, this difference did not reach statistical significance. Furthermore, although these seven patients did not achieve a PR or CR by the end of the study, there were varying degrees of the intrahepatic target lesions reduction after treatment. Among them, two patients showed tumor reduction and achieved SD status, meeting the criteria for surgical resection. Subsequently, these patients underwent surgery. Moreover, two patients developed new lesions during the initial tumor assessment, indicating the presence of micro distant metastasis before treatment, which might have been undetectable before the treatment.

From a successful conversion perspective, the most important factors to consider are the ORR and response pattern, including the primary tumor progression rate (PD), TTR, and DoR. In this study, the PD rate was only 5.6%, the median TTR was 1.8 months, and the median DoR was 7.8 months. Lower PD rate and shorter TTR indicate that fewer patients develop tumor progression during conversion therapy while having a better tumor response, which can be more effective in shrinking or even downgrading tumors and also helps to reduce the incidence of adverse reactions. A longer DoR indicates a longer tumor response duration, providing a longer time window for subsequent treatment.

In previous studies, lenvatinib combined with PD-1 inhibitors had the high ORR (36.0–54.2%) and low disease progression rates. Multiple studies reported conversion success rates ranging from 15.9% to 30.8%.^25^, ^26^, ^27^ In a prospective Phase II study by Zhang et al. evaluating lenvatinib with PD-1 inhibitors in patients with advanced uHCC, the results showed a conversion success rate of 55.4% (31/56), an ORR of mRECIST of 53.6%, an ORR of RECIST 1.1 of 44.6%, and an mOS of 23.9 months.^27^ Although similar to this study, the ORR was much lower. It is worth noting that the ORR in the study by Zhang et al. was lower than the conversion rate, indicating a relatively high proportion of oncologically unresectable cases. Combined with baseline conditions of 94.6% macrovascular invasion and 28.6% extrahepatic metastasis, it aligns more with the “neoadjuvant” concept; technically resectable initially, but with poor survival benefit due to macrovascular invasion and extrahepatic metastasis. However, in this study, patients with tumor response could not be resected if they did not meet the R0 resection criteria. This discrepancy explains why the ORR in this study was much higher than the conversion success rate. The higher ORR may be attributed to HAIC. For patients with HCC with a tumor burden concentrated in the liver or PVTT, multiple clinical studies have confirmed that HAIC treatment has a higher tumor response rate than systemic treatment.^17^^,^^24^ After HAIC treatment, some patients experienced significant tumor burden reduction or large vessel tumor thrombus regression, leading to opportunities for surgical resection or ablation treatment. Generally, four or more consecutive HAIC courses are required to achieve optimal conversion chances, but the median number of HAIC treatments in this study was three. This may be related to the combination therapy strategy of donafenib and sintilimab, which facilitated faster conversion, as indicated by the shorter TTR mentioned earlier.

Zhu et al. showed Lenvatinib, sintilimab plus transarterial chemoembolization for advanced stage hepatocellular carcinoma. The objective response rate was 60.0% per mRECIST and 30.0% per RECIST 1.1.^28^ In contrast, the ORR in this study was higher, which may be related to the advantage of HAIC in treating uHCC with PVTT. Three randomized controlled studies have shown that HAIC monotherapy or HAIC combined with sorafenib achieves an ORR of 20%–40% and mOS of 13–15 months in patients with portal vein invasion, which was superior to sorafenib or TACE monotherapy.^17^^,^^18^^,^^24^ While increasing local treatment inevitably raises costs, trauma, and side effects and complicates the evaluation of drug treatment effects, its potential benefits cannot be ignored. The key to achieving a higher ORR may be local treatments, such as TACE and HAIC.

The current OS result may not fully reveal the long-term survival outcomes because of the relatively short follow-up period. The mOS in surgical group was not reached, and is worthy of further long-term observation. The mOS of 14.3 months in non-surgical group isn’t a stable result due to the short follow-up time of 12.6 months. And 2/7 deceased patients in non-resected group didn’t receive any second-line treatment, which also has an indirect influence on the outcome.

The combination treatment used in this trial was generally well tolerated, with controllable adverse events, and no new or unexpected toxic effects were found. No patients experienced grade 5 adverse events.

This study had certain limitations. First, while inferior vena cava invasion was an exclusion criterion, three patients with inferior vena cava involvement were included based on individualized clinical assessments. This highlights the real-world challenges in balancing protocol adherence with patient-centered care in uHCC. Future studies should prospectively address whether inferior vena cava invasion modifies donafenib and sintilimab combined with HAIC response. Second, our study did not pre-specify the sample size due to the exploratory nature of the research questions. This limitation may reduce the statistical power of our study. Future studies should incorporate a priori sample size calculations to ensure adequate statistical power and robustness of the results. Third, the current follow-up duration may not be sufficient to fully reveal the long-term survival outcomes. We will continue to follow up to provide more comprehensive data on long-term outcomes. Fourth, peripheral neuropathy caused by oxaliplatin wasn’t observed with a possibility of underreporting. Last, we acknowledge that our study’s sample size was relatively small, which may affect the generalizability of our findings. And we recognize that conducting our study in a single center introduces a potential bias. This study also lacks a control group, which may limit the ability to confirm the causal relationship between the treatment and the outcomes. In order to address these issues, we are planning to conduct a larger sample size, multicenter randomized controlled trial to validate our findings and provide stronger evidence.

In conclusion, this prospective study demonstrated that HAIC combined with donafenib and sintilimab was safe and effective as a conversion therapy for patients with unresectable advanced HCC, showing good clinical efficacy and acceptable toxicity. This regimen is a potential conversion therapy strategy.

## Contributors

WG, ZL-P and XH-Z contributed equally to this paper and are co- lead authors. WZ, and WG-X contributed to the development of the protocol and data analysis and interpretation and have been active investigators. WG, ZL-P, XH-Z, TQ-S, QL, WL, WZ, and WG-X contributed to the development of the DoHAICs protocol and data analysis and interpretation and have been active investigators. WH-Z contributed to data analysis. LY, JB-C, DY-L, HJ-Z, CL, GT-L, XB, XM-L, XL-Z and BH-X contributed to the study design, data analysis, and data interpretation. All authors contributed to drafting and critical review of the manuscript. All authors read and approved the final version of the manuscript. WZ and WG had access to and verified the underlying data. WZ is the guarantor of the manuscript and accepts full responsibility for the work and/or the conduct of the study, had access to the data, and controlled the decision to publish.

## Data sharing statement

The Excel format data used to support the findings of this study are available from the corresponding author at [zhang.wei@tmu.edu.cn] upon request.

## Declaration of interests

The authors have no conflict of interest related to this study.

## Appendix ASupplementary data

Supplementary Figs. S1–S4 and Tables S1–S9

Protocol
