# Supplementary material for: Donafenib and sintilimab combined with hepatic arterial infusion chemotherapy for unresectable hepatocellular carcinoma: a prospective, single-arm phase II trial (DoHAICs study)
Source: eClinicalMedicine. 2025 May 5;83:103217. doi: 10.1016/j.eclinm.2025.103217 (PMC12138396; doi:10.1016/j.eclinm.2025.103217)
Supplement: Supplementary Figs. S1–S4 and Tables S1–S9 [file mmc1.docx]

**List of Supplementary Tables and Figures**

**Supplementary Table S1:** **Efficacy results**

**Supplementary Table S2: Baseline characteristics between responder and non-responder patients**

**Supplementary Table S3: Baseline characteristics between surgical and nonsurgical patients**

**Supplementary Table S4: Changes in AFP levels during the treatment**

**Supplementary Table S5: Changes in PIVKA-II levels during the treatment**

**Supplementary Table S6: The result of the proportional hazards assumption on event-free survival (EFS)**

**Supplementary Table S7: The result of the proportional hazards assumption on overall survival (OS)**

**Supplementary Table S8:** **Univariate and multivariate analysis of event-free survival (EFS)**

**Supplementary Table S9: Univariate and multivariate analysis of overall survival (OS)**

**Supplementary Figure S1: Spider plot of target lesions assessed per modified Response Evaluation Criteria in Solid Tumors**

**Supplementary Figure S2: Subgroup analysis of objective response rate per modified Response Evaluation Criteria in Solid Tumors**

**Supplementary Figure S3: Schoenfeld residual plots for testing the proportional hazards assumption of event-free survival. IVCTT, Inferior vena cava tumor thrombus; PVTT, portal vein tumor thrombus**

**Supplementary Figure S4: Schoenfeld residual plots for testing the proportional hazards assumption of overall survival. IVCTT, Inferior vena cava tumor thrombus; PVTT, portal vein tumor thrombus**

| **Supplementary Table S1 Efficacy results** | | |
| --- | --- | --- |
| **Variables** | **All patients (n=36)** | |
|  | **RECIST 1.1 (n=36)** | **mRECIST (n=36)** |
| Best overall response, n (%) |  |  |
| Complete response | 1 (2.8) | 4 (11.1) |
| Partial response | 20 (55.5) | 25 (69.4) |
| Stable disease | 13 (36.1) | 5 (13.9) |
| Progressive disease | 2 (5.6) | 2 (5.6) |
| Best overall response rate, % (95 % CI) | 58.3 (40.8-74.5) | 80.6 (64.0-91.8) |
| Median time to response, mo (range) | 2.1 (1.2-4.7) | 1.8 (1.2-5.2) |
| Surgical resection, n (%) | 18 (50.0) | |
| Pathological complete response, n (%) | 7 (38.9) | |
| Major pathological response, n (%) | 9 (50.0) | |
| CI, confidence interval | | |

| **Supplementary Table S2** Baseline characteristics between responder and non-responder patients | | | |
| --- | --- | --- | --- |
| **Characteristic** | **responder (n=29)** | **non-responder(n=7)** | ***p*.value** |
| Age, mean (SD), years | 59.69 (8.91) | 54.86 (10.71) | 0.223 |
| Sex, n (%) |  |  |  |
| Male | 25 (86.2) | 5 ( 71.4) | 0.706 |
| Female | 4 (13.8) | 2 ( 28.6) |  |
| ECOG PS, n (%) |  |  |  |
| 0 | 28 (96.6) | 7 (100.0) | 1.000 |
| 1 | 1 ( 3.4) | 0 ( 0.0) |  |
| HBV infection, n (%) |  |  |  |
| Yes | 25 (86.2) | 4 ( 57.1) | 0.226 |
| No | 4 (13.8) | 3 ( 42.9) |  |
| AFP, n (%), ng/mL |  |  |  |
| <400 | 18 (62.1) | 3 ( 42.9) | 0.618 |
| ≥400 | 11 (37.9) | 4 ( 57.1) |  |
| BCLC stage, n (%) |  |  |  |
| A | 4 (13.8) | 0 ( 0.0) | 0.547 |
| B | 9 (31.0) | 3 ( 42.9) |  |
| C | 16 (55.2) | 4 ( 57.1) |  |
| Extrahepatic disease, n (%) |  |  |  |
| No | 27 (93.1) | 6 ( 85.7) | 1.000 |
| Yes | 2 ( 6.9) | 1 ( 14.3) |  |
| Macrovascular invasion, n (%) |  |  |  |
| No | 14 (48.3) | 4 ( 57.1) | 1.000 |
| Yes | 15 (51.7) | 3 ( 42.9) |  |
| PVTT, n (%) |  |  |  |
| No | 16 (55.2) | 6 ( 85.7) | 0.291 |
| Yes | 13 (44.8) | 1 ( 14.3) |  |
| IVCTT, n (%) |  |  |  |
| No | 26 (89.7) | 7 (100.0) | 0.899 |
| Yes | 3 (10.3) | 0 ( 0.0) |  |
| HVTT, n (%) |  |  |  |
| No | 24 (82.8) | 5 ( 71.4) | 0.883 |
| Yes | 5 (17.2) | 2 ( 28.6) |  |
| Maximum diameter of tumor, median (range), mm | 83.00 [54.00, 110.00] | 71.00 [51.50, 147.50] | 0.984 |
| Categorical variables were compared using Pearson’s χ2 or Fisher’s exact tests. Continuous variables were compared using the t-test or Mann-Whitney U test. The sample size of the responsive group is 29, while the sample size of the non-responsive group is 7.  AFP, Alpha-fetoprotein; BCLC, Barcelona Clinic Liver Cancer; ECOG PS, Eastern Cooperative Oncology Group Performance Status; HBV, Hepatitis B Virus; HVTT, Hepatic vein tumor thrombus; IVCTT, Inferior vena cava tumor thrombus; PVTT, portal vein tumor thrombus. | | | |

| **Supplementary Table S3** Baseline characteristics between surgical and nonsurgical patients | | | |
| --- | --- | --- | --- |
| **Characteristic** | **non-surg(n=18)** | **surg(n=18)** | ***p*.value** |
| Age (mean (SD)) | 56.06 (9.33) | 61.44 (8.75) | 0.083 |
| Sex (%) |  |  |  |
| Male | 16 (88.9) | 14 ( 77.8) | 0.655 |
| Female | 2 (11.1) | 4 ( 22.2) |  |
| ECOG PS (%) |  |  |  |
| 0 | 17 (94.4) | 18 (100.0) | 1.000 |
| 1 | 1 ( 5.6) | 0 ( 0.0) |  |
| HBV infection (%) |  |  |  |
| Yes | 16 (88.9) | 13 ( 72.2) | 0.400 |
| No | 2 (11.1) | 5 ( 27.8) |  |
| AFP(%),ng/mL |  |  |  |
| <400 | 11 (61.1) | 10 ( 55.6) | 1.000 |
| ≥400 | 7 (38.9) | 8 ( 44.4) |  |
| BCLC stage(%) |  |  |  |
| A | 1 ( 5.6) | 3 ( 16.7) | 0.549 |
| B | 6 (33.3) | 6 ( 33.3) |  |
| C | 11 (61.1) | 9 ( 50.0) |  |
| Extrahepaticdisease (%) |  |  |  |
| No | 16 (88.9) | 17 ( 94.4) | 1.000 |
| Yes | 2 (11.1) | 1 ( 5.6) |  |
| PVTT (%) |  |  |  |
| No | 11 (61.1) | 11 ( 61.1) | 1.000 |
| Yes | 7 (38.9) | 7 ( 38.9) |  |
| Macrovascular invasion(%) |  |  |  |
| No | 8 (44.4) | 10 ( 55.6) | 0.739 |
| Yes | 10 (55.6) | 8 ( 44.4) |  |
| Maximum diameter of tumor(median [range]),mm | 94.50 [37.0, 187.0] | 74.50 [33.3, 193.0] | 0.117 |
| Categorical variables were compared using Pearson’s χ2 or Fisher’s exact tests. Continuous variables were compared using the t-test or Mann-Whitney U test. The sample size for each group is 18.  AFP, Alpha-fetoprotein; BCLC, Barcelona Clinic Liver Cancer; ECOG PS, Eastern Cooperative Oncology Group Performance Status; HBV, Hepatitis B Virus; PVTT, portal vein tumor thrombus. | | | |

| **Supplementary Table S4** Changes in AFP levels during the treatment | | | | |
| --- | --- | --- | --- | --- |
|  | **Log AFP (Mean (SD))** | **method** | ***p*** | **Adjusted *p* (Bonferroni)** |
| Baseline | 2.46（1.45） | Wilcoxon matched-pair signed-rank test | 0.0000011 | 0.0000033 |
| C2D1 | 1.92（1.34） |  |  |  |
| Baseline | 2.46（1.45） | Wilcoxon matched-pair signed-rank test | 0.000061 | 0.000183 |
| C3D1 | 1.61（1.28） |  |  |  |
| Baseline | 2.46（1.45） | Wilcoxon matched-pair signed-rank test | 0.0016 | 0.0048 |
| C4D1 | 1.61（1.21） |  |  |  |
| AFP, alpha-fetoprotein; C2D1, Cycle (C) 2 Day (D) 1; C3D1, Cycle (C) 3 Day (D) 1; C4D1, Cycle (C) 4 Day (D) 1. | | | | |

| **Supplementary Table S5** Changes in PIVKA-II levels during the treatment | | | | |
| --- | --- | --- | --- | --- |
|  | **Log PIVKA-II (Mean (SD))** | **method** | ***p*** | **Adjusted *p* (Bonferroni)** |
| Baseline | 3.22（1.02） | paired t-test | 0.0053 | 0.0159 |
| C2D1 | 2.76（1.16） |  |  |  |
| Baseline | 3.22（1.02） | paired t-test | 0.0011 | 0.0033 |
| C3D1 | 2.51（1.24） |  |  |  |
| Baseline | 3.22（1.02） | paired t-test | 0.0025 | 0.0075 |
| C4D1 | 2.17（1.16） |  |  |  |
| C2D1, Cycle (C) 2 Day (D) 1; C3D1, Cycle (C) 3 Day (D) 1; C4D1, Cycle (C) 4 Day (D) 1; PIVKA-II, Proteins induced by vitamin K absence or antagonist-II. | | | | |

| **Supplementary Table S6** The result of the proportional hazards assumption on event-free survival (EFS) | | | |
| --- | --- | --- | --- |
|  | **chisq** | **df** | ***p*** |
| Age | 0.6915 | 1 | 0.406 |
| BCLC stage | 0.0358 | 1 | 0.850 |
| PVTT | 0.2797 | 1 | 0.597 |
| Target tumor number | 0.5690 | 1 | 0.451 |
| Surgery | 0.0662 | 1 | 0.797 |
| IVCTT | 3.1630 | 1 | 0.075 |
| Maximum diameter of lesions | 0.5832 | 1 | 0.445 |
| GLOBAL | 8.7342 | 7 | 0.272 |
| The proportional hazards assumption was verified using Schoenfeld residuals and global tests. The sample size is 36. BCLC, Barcelona Clinic Liver Cancer; IVCTT, Inferior vena cava tumor thrombus; PVTT, portal vein tumor thrombus. | | | |

| **Supplementary Table S7** The result of the proportional hazards assumption on overall survival (OS) | | | |
| --- | --- | --- | --- |
|  | **chisq** | **df** | ***p*** |
| Age | 0.4841 | 1 | 0.487 |
| BCLC stage | 0.8053 | 1 | 0.370 |
| PVTT | 0.0385 | 1 | 0.844 |
| Target tumor number | 0.0033 | 1 | 0.954 |
| Surgery | 2.0797 | 1 | 0.149 |
| IVCTT | 3.7731 | 1 | 0.052 |
| Maximum diameter of lesions | 1.0313 | 1 | 0.310 |
| GLOBAL | 12.9714 | 7 | 0.073 |
| The proportional hazards assumption was verified using Schoenfeld residuals and global tests. The sample size is 36. BCLC, Barcelona Clinic Liver Cancer; IVCTT, Inferior vena cava tumor thrombus; PVTT, portal vein tumor thrombus. | | | |

| **Supplementary Table S8** Univariate and multivariate analysis of event-free survival (EFS) | | | | | | |
| --- | --- | --- | --- | --- | --- | --- |
| **Variable** | **Univariate** | | | **Multivariate** | | |
|  | HR | 95% CI | *p*.value | HR | 95% CI | *p*.value |
| Age (mean, ≥59 vs＜59) | 0.62 | (0.19-2.00) | 0.426 |  |  |  |
| BCLC stage (C vs B&A) | 0.88 | (0.28-2.72) | 0.818 |  |  |  |
| PVTT (yes vs no) | 0.49 | (0.13-1.80) | 0.281 |  |  |  |
| IVCTT (yes vs no) | 1.90 | (0.23-15.40) | 0.549 |  |  |  |
| Maximum diameter of lesions (median, ≥8.2cm vs ＜8.2cm) | 0.93 | (0.29-2.97) | 0.899 |  |  |  |
| Target tumor number (＞1 vs 1) | 3.68 | (0.99-13.70) | **0.052** | 1.96 | (0.49-7.84) | 0.341 |
| Surgery (yes vs no) | 0.08 | (0.02-0.38) | **0.002** | 0.10 | (0.02-0.51) | **0.006** |
| EFS was assessed according to mRECIST. All variables with p < 0.2 during univariate analyses were included in multivariate analyses, which included a Cox regression analysis to identify factors independently associated with EFS. The sample size is 36. BCLC, Barcelona Clinic Liver Cancer; IVCTT, Inferior vena cava tumor thrombus; PVTT, portal vein tumor thrombus. | | | | | | |

| **Supplementary Table S9** Univariate and multivariate analysis of overall survival (OS) | | | | | | |
| --- | --- | --- | --- | --- | --- | --- |
| **Variable** | **Univariate** | | | **Multivariate** | | |
|  | HR | 95% CI | *p*.value | HR | 95% CI | *p*.value |
| Age (mean, ≥59 vs＜59) | 1.35 | (0.39-4.69) | 0.635 |  |  |  |
| BCLC stage(C vs B&A) | 2.52 | (0.64-9.86) | **0.185** | 2.15 | (0.50-9.22) | 0.303 |
| PVTT (yes vs no) | 1.21 | (0.34-4.29) | 0.773 |  |  |  |
| IVCTT (yes vs no) | 5.28 | (1.01-27.70) | **0.049** | 4.37 | (0.74-25.87) | 0.104 |
| Maximum diameter of lesions (median, ≥82mm vs ＜82mm) | 2.29 | (0.64-8.23) | 0.205 |  |  |  |
| Target tumor number (＞1 vs 1) | 1.41 | (0.40-5.02) | 0.592 |  |  |  |
| Surgery(yes vs no) | 0.26 | (0.07-1.02) | **0.053** | 0.24 | (0.06-0.97) | **0.046** |
| All variables with p < 0.2 during univariate analyses were included in multivariate analyses, which included a Cox regression analysis to identify factors independently associated with OS. The sample size is 36. BCLC, Barcelona Clinic Liver Cancer; IVCTT, Inferior vena cava tumor thrombus; PVTT, portal vein tumor thrombus. | | | | | | |


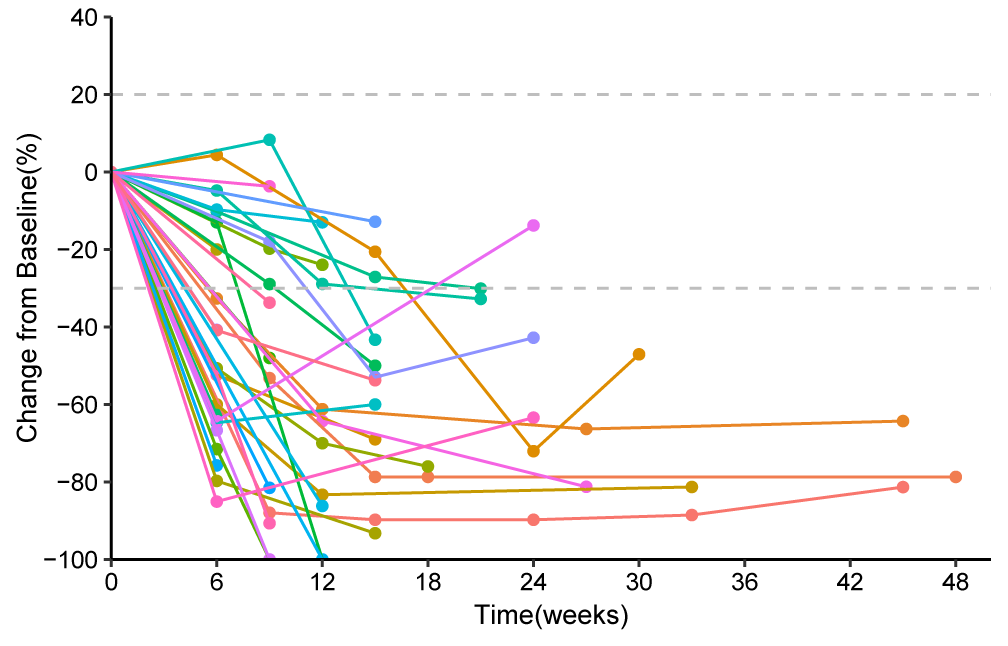


**Supplementary Figure S1** Spider plot of target lesions assessed per modified Response Evaluation Criteria in Solid Tumors.


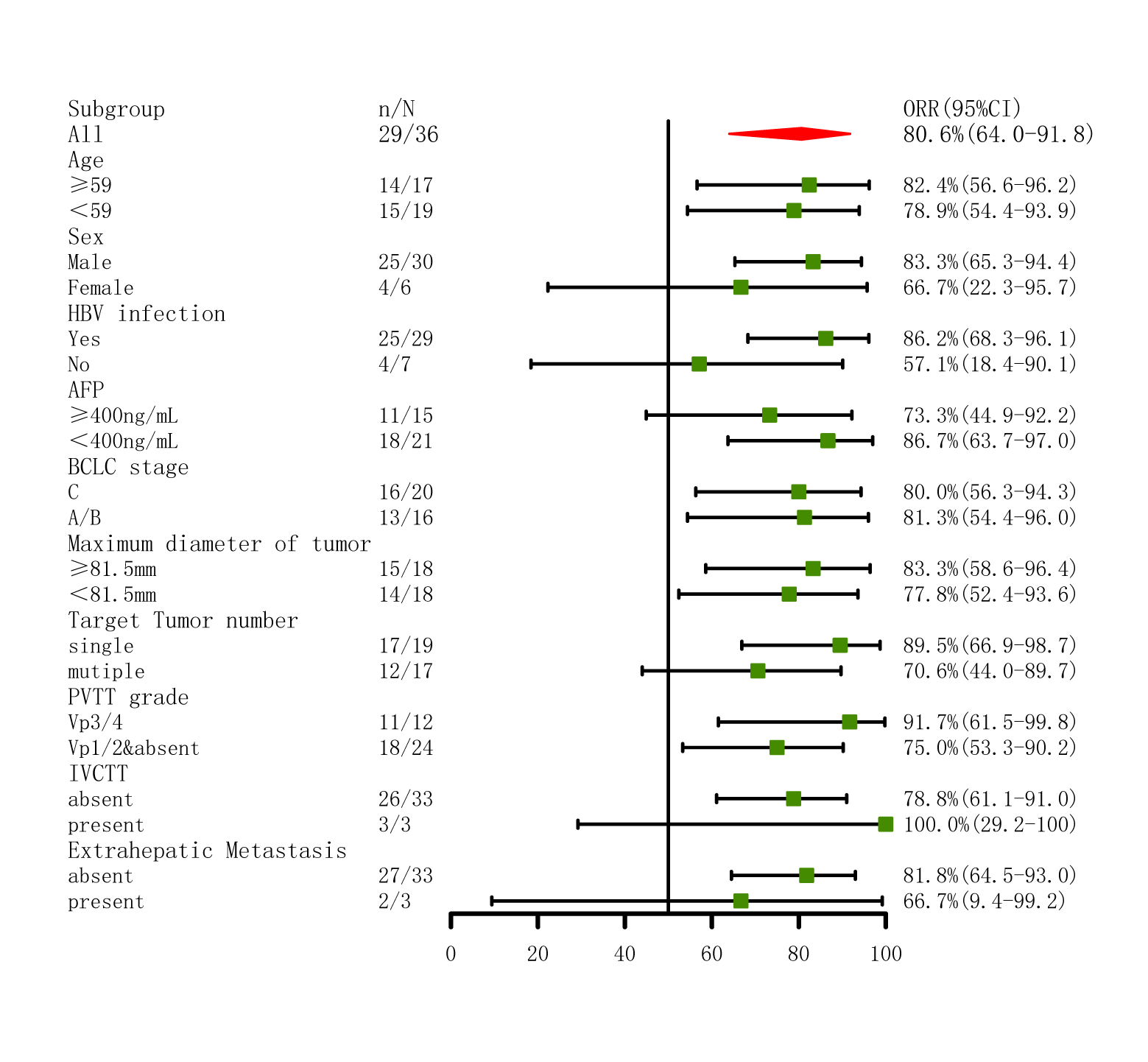


**Supplementary Figure S2** Subgroup analysis of objective response rate per modified Response Evaluation Criteria in Solid Tumors.


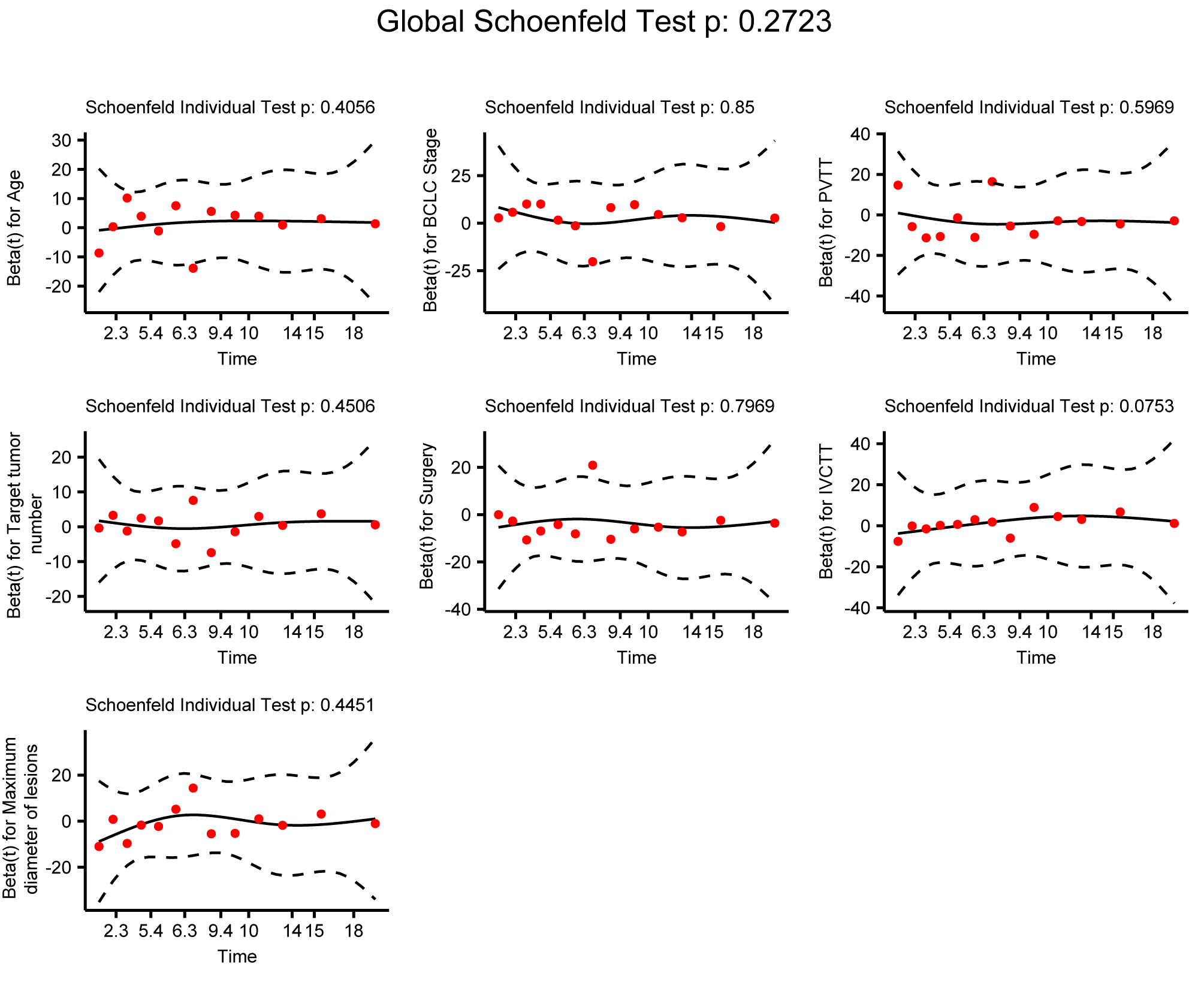


**Supplementary Figure S3** Schoenfeld residual plots for testing the proportional hazards assumption of event-free survival. IVCTT, Inferior vena cava tumor thrombus; PVTT, portal vein tumor thrombus.


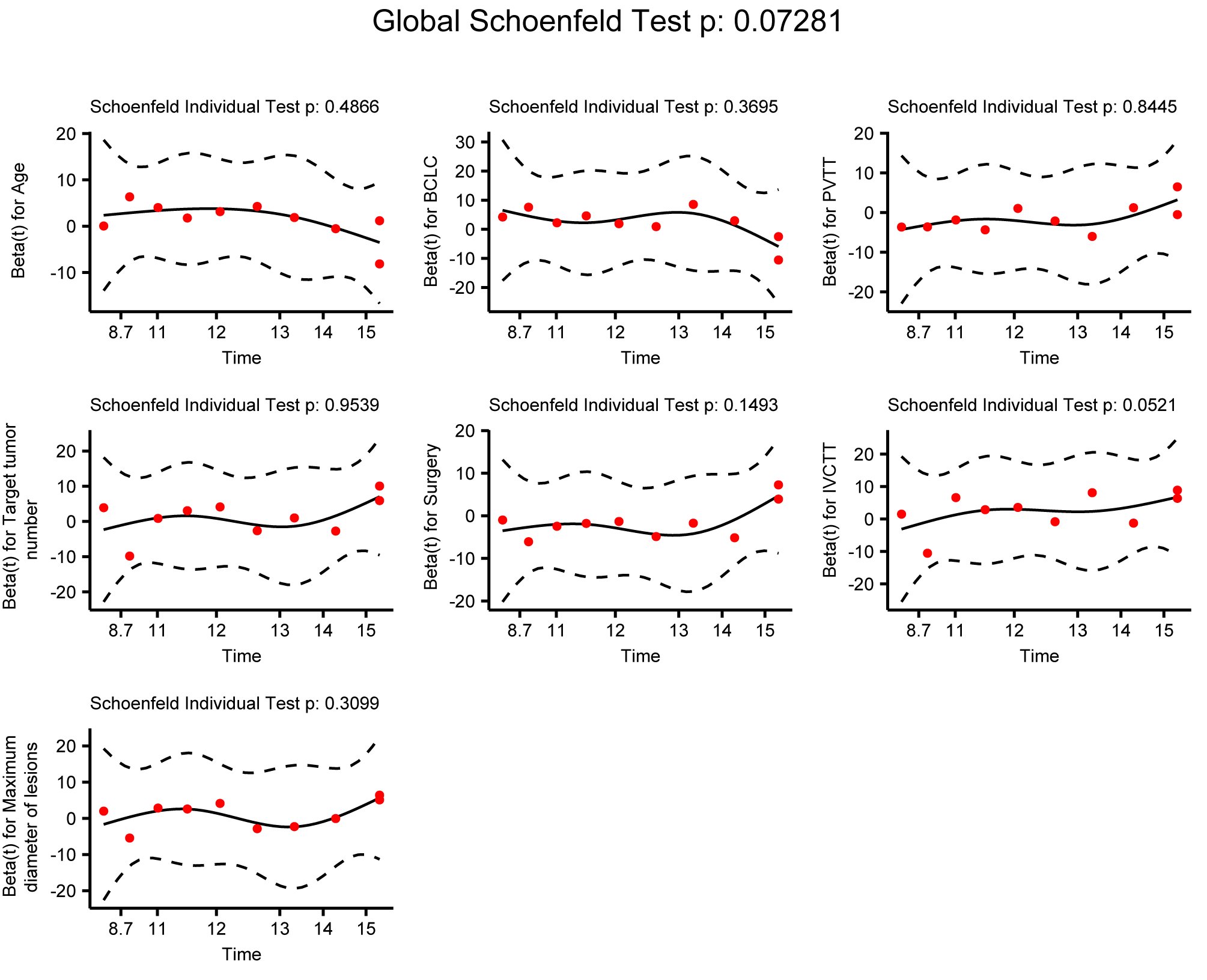


**Supplementary Figure S4** Schoenfeld residual plots for testing the proportional hazards assumption of overall survival. IVCTT, Inferior vena cava tumor thrombus; PVTT, portal vein tumor thrombus.
