# Supplementary material for: Donafenib and sintilimab combined with hepatic arterial infusion chemotherapy for unresectable hepatocellular carcinoma: a prospective, single-arm phase II trial (DoHAICs study)
Source: eClinicalMedicine. 2025 May 5;83:103217. doi: 10.1016/j.eclinm.2025.103217 (PMC12138396; doi:10.1016/j.eclinm.2025.103217)
Supplement: Protocol [file mmc2.docx]

**Protocol**

**Clinical study on the efficacy and safety of donafenib combined with hepatic arterial infusion chemotherapy and sintilimab injection as first-line treatment for unresectable hepatocellular carcinoma**

**Protocol version number:** 1.0

**Protocol version date:** June 16, 2021

**Clinical research unit:** Tianjin Medical University Cancer Institute and Hospital

**Study sponsors:** Professor Zhang Wei, Professor Gao Wei

**June 16, 2021**

**Protocol Summary**

| **Study Title** | Clinical study on the efficacy and safety of donafenib combined with hepatic arterial infusion chemotherapy and sintilimab injection as first-line treatment for unresectable hepatocellular carcinoma |
| --- | --- |
| **Protocol Number** | DPM-HCC-U10 |
| **Protocol Version/Date** | 1.0 / June 16, 2021 |
| **Study Phase** | Phase II |
| **Indication** | unresectable advanced hepatocellular carcinoma |
| **Study Objectives and Endpoints** | Primary Objectives and Endpoints:   - Evaluate the efficacy of the combination of donafenib with hepatic arterial infusion chemotherapy (HAIC) and sintilimab as first-line treatment for unresectable hepatocellular carcinoma based on objective response rate (ORR) (Note: This study will use both RECIST v1.1 and mRECIST criteria for tumor assessment, with RECIST v1.1 as the primary standard).   Secondary objectives and endpoints:   - To evaluate the efficacy and safety of the combination of donafenib with hepatic arterial infusion chemotherapy and sintilimab as first-line treatment for unresectable hepatocellular carcinoma based on overall survival (OS), time to progression (TTP), progression-free survival (PFS), disease control rate (DCR), duration of response (DOR), changes in ECOG PS scores over time, changes in AFP levels over time, as well as adverse events and serious adverse events.   (Note: This study will use both RECIST v1.1 and mRECIST criteria for tumor assessment, with RECIST v1.1 as the primary standard.) |
| **Research design** | - Single-center, open-label, single-arm, exploratory study |
| **Sample size calculation** | This study is an exploratory study with a fixed sample size of 30 cases |
| **Study duration** | 3 years |
| **Inclusion criteria** | 1. Voluntary enrollment with signed written informed consent; 2. Aged 18 to 80 years (inclusive), regardless of gender; 3. Patients with hepatocellular carcinoma clinically diagnosed according to the 'Guidelines for the Diagnosis and Treatment of Primary Liver Cancer (2019 Edition)' or confirmed by histological/cytological diagnosis; 4. Patients with unresectable or metastatic hepatocellular carcinoma; 5. No prior systemic treatment. Patients who have received adjuvant chemotherapy after local treatment may also be included if the chemotherapy ended more than 12 months ago and there has been disease progression or metastasis; 6. The last intervention, radiotherapy, and ablation treatment must have ended more than 4 weeks ago; 7. Patients who have previously undergone liver resection should have R0 resection, and tumor recurrence should occur more than 24 months after surgery; 8. There must be at least one assessable lesion (according to RECIST 1.1 criteria) ; 9. Expected survival time ≥ 3 months; 10. Eastern Cooperative Oncology Group (ECOG) performance status (PS) score of 0-1; 11. Child-Pugh score ≤ 7; 12. Able to cooperate in observing adverse events and efficacy; 13. Major organ functions are normal, meeting the following criteria:   Complete blood count (no blood transfusion or G-CSF use within 14 days prior to screening):   - 1. Hemoglobin ≥ 90 g/L;   2. Absolute neutrophil count (ANC) ≥ 1.5 × 10^9^/L;   3. Platelet count ≥ 75 × 10^9^/L;   Biochemical tests (no albumin use within 14 days prior to screening):   1. Albumin ≥ 28 g/L; 2. Total bilirubin ≤ 2 × upper limit of normal (ULN); 3. Aspartate aminotransferase (AST), alanine aminotransferase (ALT) ≤ 5 × ULN; 4. Alkaline phosphatase (ALP) ≤5×ULN; 5. Creatinine ≤1.5×ULN;   Coagulation function:   1. International normalized ratio (INR) or prothrombin time (PT) ≤1.5×ULN; 2. Activated partial thromboplastin time (APTT) ≤1.5×ULN. |
| **Exclusion criteria** | Previous or concurrent diseases:   1. Histologically/cytologically confirmed previous diagnosis of fibrolamellar hepatocellular carcinoma, sarcomatoid hepatocellular carcinoma, cholangiocarcinoma, or other components; 2. History of malignancies other than hepatocellular carcinoma, unless meeting the following criteria:    1. The patient has undergone potentially curative treatment and has no evidence of the disease for 5 years;    2. Successfully received resection for basal cell carcinoma of the skin, squamous cell carcinoma of the skin, superficial bladder cancer, cervical carcinoma in situ, and other in situ cancers; 3. Diffuse tumor lesions; 4. Tumor vascular invasion presents one or more of the following conditions:   a) Involvement of the superior mesenteric vein;  b) Involvement of the inferior vena cava;   1. History of hepatic encephalopathy, hepatorenal syndrome, or history of liver transplantation; 2. Clinical symptoms requiring drainage of pleural effusion, ascites, or pericardial effusion; 3. Central nervous system metastasis; 4. History of severe mental illness; 5. Diseases affecting the absorption, distribution, metabolism, or clearance of the study drug (such as severe vomiting, chronic diarrhea, intestinal obstruction, malabsorption, etc.);   Previous or concomitant medications/treatments:   1. Previous receipt of allogeneic stem cell or solid organ transplantation; 2. Previous treatment with targeted therapies against VEGF and/or VEGFR, RAF, MEK signaling pathways such as sorafenib, lenvatinib, regorafenib, or immune modulators such as anti-PD-1, anti-PD-L1, anti-CTLA-4; 3. Patients who have previously received other systemic anti-tumor therapies, including traditional Chinese medicine with anti-tumor indications, within less than 2 weeks prior to the study medication, or those whose adverse events from previous treatments have not recovered to ≤ CTCAE Grade 1; Toxic reactions from prior cancer treatments do not include alopecia and Grade 1/2 neurotoxicity caused by oxaliplatin; 4. Concurrent use of medications that may prolong QTc and/or induce Torsades de Pointes (TdP), or medications that affect drug metabolism; 5. A history of or currently having congenital or acquired immunodeficiency diseases; 6. Active or previously recorded autoimmune diseases or inflammatory diseases (including but not limited to: autoimmune hepatitis, interstitial pneumonia, inflammatory bowel disease, systemic lupus erythematosus, vasculitis, uveitis, pituitary inflammation, hyperthyroidism or hypothyroidism, asthma requiring bronchodilator treatment, etc.), individuals with vitiligo or asthma that has completely resolved in childhood, and those who require no intervention in adulthood may be included; 7. Previous receipt of allogeneic stem cell or solid organ transplantation; 8. Patients who have used systemic immunosuppressive therapy within 2 weeks prior to enrollment, or are expected to require systemic immunosuppressive therapy during the study, except for the following situations: 9. Intranasal, inhaled, topical, or local injection (e.g., intra-articular injection) corticosteroids; 10. Doses not exceeding 10 mg/day of prednisone or other equivalent systemic corticosteroids; 11. Corticosteroids used prophylactically for hypersensitivity reactions;   Safety:   1. Patients with known or suspected allergy to donafenib or similar drugs, or a history of hypersensitivity reactions to chimeric or humanized antibodies or fusion proteins, or those allergic to the excipients of the investigational drug; 2. Active bleeding or coagulation dysfunction, with a tendency to bleed or currently undergoing thrombolysis, anticoagulation, or antiplatelet therapy; 3. History of thrombosis or thromboembolic events within the past 6 months, such as stroke and/or transient ischemic attack, deep vein thrombosis, pulmonary embolism, etc.; 4. History of esophageal or gastric variceal bleeding events due to portal hypertension within the past 6 months, or any life-threatening bleeding events occurring within the past 3 months; 5. Significant clinically meaningful cardiovascular diseases, including but not limited to acute myocardial infarction, severe/uncontrolled angina, or coronary artery bypass surgery within the past 6 months, congestive heart failure (NYHA classification >2), poorly controlled or requiring pacemaker treatment for arrhythmias, and hypertension that is not controlled by medication (systolic blood pressure ≥140 mmHg and/or diastolic blood pressure ≥90 mmHg); 6. Other significant clinical and laboratory abnormalities that the investigator believes affect the safety assessment, such as: uncontrolled diabetes, chronic kidney disease, grade II or higher peripheral neuropathy (CTCAE V5.0), thyroid dysfunction, etc.; 7. Severe infections that are active or poorly controlled clinically; Active infections, including:   a) Positive for human immunodeficiency virus (HIV) (HIV1/2 antibodies);  b) Active hepatitis B (positive HBsAg or HBV DNA > 2000 IU/ml and abnormal liver function);  c) Active hepatitis C (HCV antibody positive or HCV RNA ≥ 10  ^3^ copies/ml and liver function abnormal);  d) Active tuberculosis;  e) Other uncontrolled active infections (CTCAE V5.0 > grade 2);   1. Not yet recovered from surgery, such as having unhealed incisions or severe postoperative complications; 2. Pregnant or breastfeeding women, as well as female or male patients of childbearing potential who are unwilling or unable to take effective contraceptive measures. |
| **Research Process** | **Screening Period:**  The investigator must fully inform the patient of the potential benefits and risks of this clinical trial. After the patient voluntarily signs the informed consent form, various screening examinations will begin, including the following items:  1) Collect medical history, smoking history, alcohol history, and demographic data  2) Vital signs, physical examination, blood pressure monitoring, ECOG score  3) Adverse events, concomitant medication  4) Laboratory tests   - Complete blood count: including red blood cell count, hemoglobin, hematocrit, white blood cell count and differential (neutrophils, lymphocytes, eosinophils, monocytes, basophils) and platelet count; - Blood biochemistry: including total protein, albumin, globulin, blood glucose, urea/urea nitrogen, creatinine, alkaline phosphatase, lactate dehydrogenase, total bilirubin, direct bilirubin, indirect bilirubin, AST, ALT, γ-glutamyl transferase, calcium, phosphorus, magnesium, potassium, sodium, chloride, amylase, uric acid, creatine kinase, troponin; - Virology tests: Hepatitis B qualitative five items and HBV-DNA, HCV-RNA, HIV; - Tumor markers: AFP; - Thyroid function: TSH, free T3, free T4; - Urinalysis: including specific gravity, pH, urine glucose, protein, casts, ketones, urine red blood cells, urine white blood cells; If there are two consecutive urine protein tests showing ++ or higher, or if the doctor determines the results to be abnormal and clinically significant, a 24-hour urine protein quantification test is required; - Fecal occult blood; - Coagulation function: TT, PT, INR, APTT; - Cardiac ultrasound examination: particularly pay attention to the evaluation of left ventricular ejection function; - 12-lead electrocardiogram (ECG); - Imaging examination: CT or MRI (Note: examinations conducted within 4 weeks prior to screening at the same institution are acceptable); - Women of childbearing age must undergo pregnancy testing to exclude pregnancy;   If all the above screening examination items meet the enrollment criteria for this study, the patient will be enrolled.  **Treatment period:**  After enrollment, subjects will first take oral donafenib tablets for 3 to 7 days. After the investigator assesses that the subjects can tolerate it, the subjects will receive intravenous infusion treatment with sintilimab injection. On the day of sintilimab infusion or the following day, they will begin approximately 1 day of HAIC treatment (the total number of subsequent HAIC treatments will be conducted as needed by the investigator based on the patient's actual condition). Afterwards, subjects will visit every 3 weeks (calculated from the day of the first infusion of sintilimab), with a time window of ±3 days. Every 6 weeks, imaging assessments will evaluate efficacy (using both RECIST v1.1 and mRECIST criteria for tumor assessment), until the subject first shows imaging-confirmed disease progression (meeting both RECIST v1.1 and mRECIST criteria for imaging progression) or intolerable toxicity.  **Follow-up after treatment ends:**  Patients need to undergo safety follow-up and survival follow-up after treatment ends.  In addition, for patients who have not progressed but discontinued treatment due to intolerance, follow-up is required every 12 weeks (within 2 years after the first administration) or every 24 weeks (after 2 years of the first administration) until definitive imaging evidence of progression or death is obtained.  For specific follow-up times and related examination items, please refer to the flowchart. |
| **Administration dose** | 1. **Donafenib** : 200 mg, Bid, to be taken orally starting 3-7 days before the first HAIC treatment; 2. **Sintilimab**: 200 mg, Q3W, intravenous infusion 0-1 days before each HAIC treatment; 3. **HAIC:** FOLFOX regimen hepatic arterial infusion, Q3W, the total number of HAIC treatments will be determined by the investigator based on the patient's actual condition as needed (chemotherapy dose: Oxaliplatin 85 mg/m^2^ infusion over 2 hours, Leucovorin 400 mg/m^2^ infusion over 2 hours, 5-Fu 400 mg/m^2^ bolus over 10 minutes, followed by continuous infusion of 5-Fu 1200 mg/m^2^ over 23 hours);   **Note:**  This study sets a safety lead-in period, during which the first 6 subjects enrolled in the study will undergo gradual safety monitoring. Each patient will receive oral administration of donafenib [200 mg, Bid] in combination with intravenous infusion of sintilimab injection [200 mg, Q3W], along with HAIC treatment. The monitoring window is from the first administration of donafenib treatment to 21±3 days after the first HAIC treatment (i.e., from D1 of the donafenib monotherapy period to C2D1 of the combination therapy period). If ≥2 cases of dose-limiting toxicity (DLT) are observed among the first 6 subjects, and the research team assesses that the toxicity is solely related to donafenib treatment, the initial dose for subsequent enrolled patients receiving donafenib treatment will be adjusted to 100 mg Bid.  During the safety induction period, if a subject does not complete the tolerance observation period (approximately 21±3 days after the first HAIC treatment) due to non-dose tolerance reasons, they will be replaced by a new subject.  After the safety induction period, any enrolled subjects who withdraw from the trial early are not allowed to have additional subjects enrolled as replacements. Furthermore, the corresponding number of the withdrawn subject is not allowed to be reused by other new subjects. |
| **DLT Assessment Principles** | DLT is defined as any of the following adverse events, unless the investigator determines that it is definitely related to the study progression or caused by other external factors, it will be considered as DLT:  Hematologic Toxicity:  1. Grade 4 neutropenia lasting more than 7 days.  2. Febrile neutropenia [defined as an absolute neutrophil count (ANC) < 1×10^9^ /L, with a single temperature reaching 38.3℃ or a temperature of 38℃ persisting for more than one hour].  3. Grade 3 neutropenia with infection.  4. Grade 3 thrombocytopenia with severe uncontrolled bleeding events.  5. Grade 4 thrombocytopenia.  6. Grade 4 anemia (life-threatening).  7. Grade 3 or 4 lymphopenia with opportunistic infections.  Non-hematologic toxicity:  1. Skin reactions: Severe hand-foot skin reactions (HFSR) or other skin adverse reactions that persist for more than 2 weeks despite appropriate intervention measures or occur a second time.  2. Blood pressure: Persistent/recurrent systolic blood pressure ≥160 mmHg or diastolic blood pressure ≥100 mmHg, and cannot be controlled despite aggressive antihypertensive treatment. (Persistent/recurrent blood pressure ≥160/100 mmHg refers to measurements of ≥160/100 mmHg taken at least twice with an interval of 24 hours, with each measurement taken after the subject has been seated and resting quietly for more than 5 minutes.) At least 1 week of antihypertensive medication is allowed before the second recorded hypertension event occurs.  3. Cardiac contractile function: The first occurrence of LVEF decrease >10%, or LVEF value <40%.  4. Gastrointestinal system: Grade 4 diarrhea, or grade 3 diarrhea lasting >24 hours despite active antidiarrheal treatment, or occurrence of two episodes of grade 3 diarrhea regardless of duration; Occurrence of ≥ grade 3 nausea and vomiting despite adequate/maximum medical intervention and/or preventive measures.  5. Absolute QTcB ≥ 500 msec (at two consecutive time points).  6. Other grade 4 or higher adverse events.  7. Grade 3 or higher immune-related adverse events (irAE).  8. Other grade 3 toxic effects, except for laboratory abnormalities that recover to grade 2 (inclusive) or below within 3 days (asymptomatic and do not require medical intervention).  In addition to the above events, DLT events also include any level of toxic reaction that the investigator considers necessitating the subject's withdrawal from the study. |
| **Dose adjustment scheme** | 1) Donafenib dose adjustment scheme: If grade 4 hematologic toxicity or grade 3 non-hematologic toxicity occurs, temporarily discontinue the drug (according to NCI CTCAE V5.0 standards). After the first suspension of medication, if the adverse reactions recover to ≤ Grade 1 within 1 week, continue taking the original dose; If recovery to ≤ Grade 1 occurs within 2 weeks of suspension, then reduce the dose to 200 mg qd; If the dose is adjusted and medication is suspended again, if recovery to ≤ Grade 1 occurs within 2 weeks, then reduce the dose to 200 mg qod, with a maximum of 2 dose adjustments allowed. If recovery to normal or CTCAE Grade 1 cannot be achieved within 2 weeks after stopping the medication, then permanently discontinue the medication. If Grade 4 hematologic toxicity or Grade 3 non-hematologic toxicity occurs again (the 3rd occurrence), then permanently discontinue the medication.  At an initial dose of 200 mg bid, the dose adjustment scheme is as follows:   \| Initial dose \| First dose reduction \| Second dose reduction \| \| --- \| --- \| --- \| \| 200mg bid \| 200mg qd \| 200mg qod \|   If the initial dose of donafenib is adjusted to 100 mg bid due to the observation of ≥2 cases of DLT in the first 6 patients, the dose adjustment principles will be consistent with those at 200 mg bid, and the specific dose adjustment scheme is as follows:   \| Initial dose \| First dose reduction \| Second dose reduction \| \| --- \| --- \| --- \| \| 100mg bid \| 100mg qd \| 100mg qod \|  1. **Sintilimab dose adjustment scheme**: Dose adjustment of sintilimab is not permitted throughout the study; when intolerable toxicity related to sintilimab occurs, the investigator may decide to suspend or permanently discontinue sintilimab administration.   If a subject experiences a grade 2 drug-related irAE or a grade 3 or higher other AE (refer to the ESMO guidelines for the management of toxicity related to immune checkpoint inhibitors), the subject must suspend the administration of Sintilimab, with a maximum delay of 7 days; the subsequent administration schedule will be adjusted accordingly. If the administration is delayed for more than 7 days, it will be considered a missed dose, and the subject will receive the next cycle of administration according to the original schedule (calculated from the date of the first administration of Sintilimab). If treatment-related adverse reactions have not recovered to grade 0-1 or baseline levels within 6 weeks after the last dose of Sintilimab injection, the administration of Sintilimab injection should be permanently discontinued. Additionally, if the subject discontinues the medication for more than 6 weeks and the investigator determines that the risks of continuing treatment outweigh the benefits, the subject must permanently discontinue Sintilimab.  3) HAIC: In the HAIC chemotherapy regimen, only the dose of Oxaliplatin will be adjusted. That is: when the tumor diameter > 10 cm and there is abundant blood supply, the dose of Oxaliplatin selected is 130 mg/m^2^. For tumors ≤ 10 cm and with less abundant blood supply, the dose of Oxaliplatin selected is 85 mg/m^2^. Additionally, when a large tumor significantly shrinks after several courses of HAIC treatment, a dose reduction may be appropriate, even down to 60 mg/m^2^. Subsequent researchers can make appropriate dose adjustments based on the patient's actual treatment situation and tolerance, and ensure proper documentation.  ***Note**: If a subject permanently discontinues one of the investigational drugs (Donafenib or Sintilimab) due to adverse reactions, and the investigator assesses that the subject may still benefit from continuing the other drug in combination with HAIC treatment, the subject may receive treatment with the other drug in combination with HAIC in this study. If a subject permanently discontinues both drugs due to adverse reactions, the subject must permanently withdraw from the study treatment. |
| **Termination Criteria** | Early Termination of the Study The investigator may terminate this study at any time, with reasons for early termination including but not limited to:   1. The discovery of unexpected, significant, or unacceptable risks to subjects enrolled in the study during the study period; 2. The discovery of significant errors in the clinical research protocol during the study period, making it difficult to evaluate the investigational drug; 3. Request for termination by health/regulatory authorities.  Subject Early Termination (Dropout or Withdrawal) Subjects have the right to withdraw from the investigational drug treatment or to withdraw their informed consent to participate in the study at any stage of the study for any reason, without losing any benefits or facing any penalties.  The investigator may terminate a subject's investigational drug treatment at any stage of the study for the following reasons:   1. The first occurrence of radiologically confirmed disease progression (concurrently meeting both RECIST v1.1 and mRECIST criteria for radiological progression) or a rapid deterioration of liver function to Child-Pugh Class C after treatment, and the subject has not recovered to a level that allows for continued study treatment within 3 weeks as assessed by the investigator; 2. Death from any cause; 3. If an intolerable adverse event (AE) occurs and does not resolve after dose adjustment or suspension (if the AE can be determined to be an adverse drug reaction (ADR) related to one of the investigational drugs, and the investigator believes that the subject would benefit from continuing the use of the other investigational drug in combination with HAIC treatment, then the subject may continue to use the other investigational drug in combination with HAIC treatment until the completion of the study treatment or the occurrence of other circumstances that necessitate early withdrawal/termination of the study); 4. The use of drugs or other substances that may provoke toxicity or lead to bias in study results, or if the investigator determines that continued treatment is detrimental to the subject's health; 5. Clinical signs or laboratory test results suggest pregnancy; 6. Subject compliance serious adverse ; 7. Subject withdraws informed consent; 8. Occurrence of concomitant diseases that prohibit the continuation of the study, or the subject requires treatment excluded by the study protocol; 9. Development of another type of cancer, excluding non-melanoma skin cancers (such as basal cell carcinoma, squamous cell carcinoma), if deemed curable and not life-threatening by the investigator; 10. Initiation of other antitumor therapy before confirmed disease progression; 11. Loss to follow-up;   12) Any other reasons confirmed by the investigator |
| **Statistical analysis** | The statistical analysis plan will be developed after the protocol is finalized and will be finalized before database lock. The statistical analysis plan will specify and describe in detail all statistical analyses to be conducted based on the main features of the protocol. All statistical analyses will be computed using SAS statistical analysis software.  This study will provide statistical descriptions of all variables obtained at each observation time point, grouped by dose, unless the protocol specifies that statistical descriptions are not required at specific time points. Overall, continuous variables (such as age) will be statistically described using the number of observations, mean, median, standard deviation, minimum, and maximum; Categorical variables will be statistically described using the frequency and percentage of each category. The final analysis of the study will be based on data collected from subjects throughout the study period. Statistical methods will be detailed in the statistical analysis plan.  The results of this trial will primarily use descriptive statistical analysis. For continuous data, the mean, standard deviation, median, maximum, and minimum will be listed; for count data and ordinal data, the frequency (composition ratio), rate, and 95% confidence interval (CI) will be provided. Describe the number of subjects enrolled in each dose group, the cases of dropout and exclusion, and perform descriptive statistical analysis on the baseline characteristics of the enrolled cases.  **Evaluation of efficacy:**  **Primary efficacy endpoints:** Efficacy analysis is based on the number of subjects achieving the primary endpoint indicators after treatment.  The number of subjects, percentage, and 95% confidence interval for ORR are estimated using the Clopper-Pearson method.  **Secondary efficacy endpoints:** Efficacy analysis is based on the number of subjects achieving the secondary endpoint indicators after treatment.  The median and 95% confidence intervals for PFS, OS, TTP, and DOR are estimated using the Kaplan-Meier method (PROC LIFETEST), and survival curves are plotted.  DCR is summarized, including the number of subjects with DCR, percentage, and 95% confidence interval (calculated using the Clopper-Pearson method).  The comparison of pre- and post-treatment changes in ECOG PS score and AFP values will be conducted using paired t-test.  **Safety Evaluation:**  Descriptive statistical analysis will be primarily used to list and describe the adverse events (AEs) and adverse reactions that occurred in each dose group during this trial (where adverse reactions are defined as AEs that are 'definitely related, probably related, or possibly related to the investigational drug'). Laboratory test results will describe situations where values were normal before the trial but abnormal after treatment, as well as the relationship between these abnormal changes and the investigational drug. |

# Abbreviation List

| ACEI | Angiotensin-converting enzyme inhibitors |
| --- | --- |
| ADR | Drug adverse reactions |
| AE | adverse events |
| AFP | Alpha-fetoprotein |
| APTT | Activated Partial Thromboplastin Time |
| ALP | Alkaline Phosphatase |
| ALT | Alanine Aminotransferase |
| Ames | Mutagenicity Testing of Contaminants |
| ANC | Neutrophil Count |
| ARDS | Acute Respiratory Distress Syndrome |
| AST | Aspartate Aminotransferase |
| AT1 | Angiotensin II Receptor |
| AUC _0-t_ | Area Under the Concentration-Time Curve from the First Dose to the Set Time |
| AUC _0-t,ss_ | Area Under the Concentration-Time Curve from the Last Dose to the Set Time |
| AUCss | Area Under the Steady-State Concentration-Time Curve |
| Bid | Twice Daily |
| NMPA | National Medical Products Administration |
| NYHA | New York Heart Association |
| CHL | Chinese Hamster Lung Cells |
| CI | Confidence Interval |
| C _max_ | Peak Concentration |
| CR | Complete Remission |
| CRF | Case Report Form |
| CRO | Contract Research Organization |
| CT | Computed Tomography |
| CTCAE | Common Adverse Event Evaluation Criteria |
| CTL | Cytotoxic T Lymphocytes |
| CTLA-4 | Cytotoxic T Lymphocyte Antigen-4 |
| DCR | Disease control rate |
| DLT | Dose-Limiting Toxicity |
| dMMR | Mismatch Repair Deficiency |
| DOR | Duration of Response |
| EC | Ethics Committee |
| ECG | Electrocardiogram |
| ECOG | Eastern Oncology Cooperative Group |
| ERK | Extracellular Regulating Protein Kinase |
| FAAN | Food Allergies and Allergy Networks |
| FACT-Hep | Liver and Gallbladder Scale of the Cancer Treatment Function Evaluation System |
| FAS | Full Analysis Set |
| FDA | U.S. Food and Drug Administration |
| FDG-PET | Fluorodeoxyglucose Positron Emission Tomography |
| FT3 | Free Triiodothyronine |
| FT4 | Free Thyroxine |
| GCP | Good Clinical Practice for Drug Trials |
| G-CSF | Granulocyte Colony-Stimulating Factor |
| HAIC | Hepatic Arterial Infusion Chemotherapy |
| HBcAb | Hepatitis B Virus Core Antibody |
| HBeAb | Hepatitis B Virus e Antibody |
| HBeAg | Hepatitis B Virus e Antigen |
| HBsAb | Hepatitis B Virus Surface Antibody |
| HBsAg | Hepatitis B Virus Surface Antigen |
| HBV | Hepatitis B Virus |
| HCC | Hepatocellular Carcinoma |
| HCV | Hepatitis C Virus |
| HFSR | Hand-Foot Syndrome |
| HIV | Human Immunodeficiency Virus |
| HNSTD | Maximum Tolerated Dose |
| HR | Risk Ratio |
| IB | Investigator's Brochure |
| ICC | Intrahepatic Cholangiocarcinoma |
| ICF | Informed Consent Form |
| ICH | International Conference on Harmonization of Technical Requirements for Registration of Pharmaceuticals for Human Use |
| IFN | interferon |
| IL-6R | interleukin-6 receptor |
| IMRT | intensity-modulated radiotherapy |
| INR | international normalized ratio |
| irAE | immune-related adverse events |
| irRECIST | immune-related solid tumor efficacy evaluation criteria |
| JAK | Janus kinase |
| LVEF | left ventricular ejection fraction |
| MDSC | bone marrow-derived suppressor cells |
| MedDRA | International Medical Terminology Dictionary |
| MEK | mitogen-activated protein kinase |
| MRI | magnetic resonance imaging |
| MSI-H | microsatellite instability high |
| MTD | maximum tolerated dose |
| MVI | microvascular invasion |
| NIAID | National Institute of Allergy and Infectious Diseases |
| NOAEL | No adverse reaction dose level observed |
| NSAID | Non-steroidal anti-inflammatory drugs |
| ORR | Objective response rate |
| OS | Overall survival |
| PDGFR | Platelet-derived growth factor receptor |
| PD-1 | Programmed cell death receptor-1 |
| PD-L1 | Programmed cell death ligand-1 |
| PEF | Peak expiratory flow rate |
| PEG | Polyethylene glycol |
| PET | Positron emission tomography |
| PFS | Progression-free survival |
| PK | Pharmacokinetics |
| PPS | Protocol compliance |
| PR | Partial response |
| PS | Performance status |
| PT | Prothrombin time |
| PT | Preferred terminology |
| PVTT | Portal vein tumor thrombus |
| Qd | Once daily |
| Qod | Once every other day |
| Q3W | Once every 3 weeks |
| RECIST | Response Evaluation Criteria in Solid Tumors |
| RFS | Progression-free survival |
| SAE | serious adverse events |
| SBRT | Stereotactic radiotherapy |
| SD | Disease stability |
| SOC | System organ classification |
| SS | Safety analysis set |
| t _1/2_ | Elimination half-life |
| TACE | Transarterial chemoembolization |
| TEAE | Adverse events occurring during treatment |
| TKI | Tyrosine kinase inhibitors |
| T _max_ | Time to peak |
| TNF | Tumor necrosis factor |
| Treg | Regulatory T cells |
| TSH | Thyroid-stimulating hormone |
| T4 | Total Thyroxine |
| TT | Thrombin Time |
| TTP | Time to Disease Progression |
| TTR | Time to Tumor Recurrence |
| TTUP | Time to Unresectable Progression |
| ULN | Upper Limit of Normal Values |
| VEGF | Vascular Endothelial Growth Factor |
| VEGFR | Vascular Endothelial Growth Factor Receptor |

**Table of Contents**

[Protocol Summary 3](#_Toc6461)

[List of Abbreviations 11](#_Toc8364)

[1 Research Background 18](#_Toc6650)

[1.1 Hepatocellular Carcinoma 18](#_Toc6991)

[1.2 Hepatic Arterial Infusion Chemotherapy (HAIC) 18](#_Toc263)

[1.3 Donafenib (referred to as Donafenib) 19](#_Toc15976)

[1.3.1 Preclinical Studies of donafenib 20](#_Toc30616)

[1.3.2 Clinical research on donafenib 21](#_Toc326)

[1.4 Recombinant humanized anti-PD-1/PD-L1 monoclonal antibody 24](#_Toc26865)

[1.5 Research basis 24](#_Toc30337)

[1.6 Benefit-risk assessment 25](#_Toc18445)

[2 Research objectives 27](#_Toc19683)

[2.1 Primary objectives 27](#_Toc22881)

[2.2 Secondary objectives 27](#_Toc3226)

[3 Research design 28](#_Toc11203)

[3.1 Overview of research design 28](#_Toc6909)

[3.2 Basis for sample size calculation 28](#_Toc16802)

[3.3 Basis for dose selection 28](#_Toc8549)

[3.3.1 Donafenib tablets 28](#_Toc13557)

[3.3.2 Sintilimab injection 28](#_Toc6124)

[3.3.3 Hepatic Arterial Infusion Chemotherapy (HAIC) 28](#_Toc13183)

[3.4 DLT Assessment and Criteria 29](#_Toc31508)

[4 Study Population 31](#_Toc24157)

[4.1 Inclusion Criteria 31](#_Toc11163)

[4.2 Exclusion Criteria 32](#_Toc12035)

[4.3 Termination Criteria 34](#_Toc12816)

[4.3.1 Early Termination of Study 34](#_Toc21772)

[4.3.2 Early Termination of Subjects (Dropout or Withdrawal) 34](#_Toc11231)

[4.3.3 Handling of Early Withdrawn Subjects 35](#_Toc23427)

[4.4 Exclusion Criteria 35](#_Toc6553)

[5 Investigational Drug 36](#_Toc2311)

[5.1 Investigational Drug Information 36](#_Toc20959)

[5.2 Treatment Protocol 36](#_Toc24223)

[5.3 Dose Adjustment/Suspension of Medication 37](#_Toc15485)

[5.4 Management of Investigational Drug Error! Bookmark not defined.](#_Toc10421)

[5.4.1 Support for Investigational Drug 38](#_Toc23556)

[5.4.2 Packaging and Labeling of Investigational Drug 41](#_Toc32230)

[5.4.3 Receipt and Storage of Investigational Drug 41](#_Toc14782)

[5.4.4 Dispensing and Retrieval of Investigational Drug 41](#_Toc25324)

[5.4.5 Treatment/Medication Adherence 42](#_Toc19893)

[5.5 Concomitant Medication and Concomitant Treatment 43](#_Toc9487)

[5.6 Prohibited Medications and Treatments 43](#_Toc14283)

[5.7 Drug Overdose 44](#_Toc21865)

[6 Research Process 45](#_Toc23180)

[6.1 Screening Period 45](#_Toc21215)

[6.2 Donafenib Monotherapy Period 47](#_Toc11687)

[6.3 Combination Therapy Period 47](#_Toc311)

[6.4 End of Treatment Visit 49](#_Toc3387)

[6.5 Follow-up Period 49](#_Toc32199)

[6.5.1 Safety Follow-up 49](#_Toc13386)

[6.5.2 Efficacy Follow-up 50](#_Toc26238)

[6.5.3 Survival Follow-up 50](#_Toc17432)

[7 Evaluation Indicators 51](#_Toc11868)

[7.1 Primary Efficacy Evaluation Indicators 51](#_Toc1861)

[7.2 Secondary Efficacy Evaluation Indicators 51](#_Toc4764)

[7.3 Safety Evaluation Indicators 51](#_Toc22233)

[7.4 Efficacy Endpoint Definitions 51](#_Toc7692)

[7.4.1 Progression-Free Survival (PFS) 51](#_Toc30884)

[7.4.2 Overall Survival (OS) 52](#_Toc21622)

[7.4.3 Time to Progression (TTP) 52](#_Toc18843)

[7.4.4 Objective Response Rate (ORR) 52](#_Toc11785)

[7.4.5 Disease Control Rate (DCR) 52](#_Toc23576)

[8 Safety Monitoring and Reporting 53](#_Toc2832)

[8.1 Adverse Events (AE) 53](#_Toc12586)

[8.2 Serious Adverse Events (SAE) 53](#_Toc19082)

[8.3 Special Management of Adverse Events for Donafenib Tablets 55](#_Toc11606)

[8.3.1 Hand-foot syndrome (HFSR) 55](#_Toc16988)

[8.3.2 Special Management of Targeted Drug-Related Hypertension 55](#_Toc11388)

[8.3.3 Gastrointestinal Bleeding 58](#_Toc3635)

[8.3.4 Proteinuria 58](#_Toc30138)

[8.3.5 Diarrhea 58](#_Toc4688)

[8.3.6 Elevated Bilirubin 58](#_Toc6014)

[8.3.7 Elevated Transaminases 59](#_Toc14801)

[8.4 Management of Special Adverse Events for Recombinant Humanized Anti-PD-1 Monoclonal Antibody 59](#_Toc13616)

[8.4.1 Infusion-related Reactions 59](#_Toc29755)

[8.4.2 Severe Allergic Reactions 60](#_Toc23316)

[8.4.3 Immune-related Adverse Events (irAE) 61](#_Toc21579)

[8.5 Assessment of Causality of Adverse Events 61](#_Toc21076)

[8.5.1 Principles for Determining the Relationship Between Adverse Events and Investigational Drug 61](#_Toc601)

[8.5.2 Criteria for Determining the Relationship Between Adverse Events and Investigational Drug 62](#_Toc32346)

[8.6 Assessment of Severity of Adverse Events 63](#_Toc1750)

[8.7 Recording and Reporting of Adverse Events and Serious Adverse Events 63](#_Toc21976)

[8.7.1 Recording and Reporting of Adverse Events 63](#_Toc21925)

[8.7.2 Reporting of Serious Adverse Events 64](#_Toc23857)

[8.7.3 Reporting and Follow-up of Pregnancy 65](#_Toc2407)

[8.8 Recording Procedures for Adverse Events 65](#_Toc29990)

[8.8.1 Infusion-related Reactions 66](#_Toc30190)

[8.8.2 Diagnosis and Signs and Symptoms 66](#_Toc10231)

[8.8.3 Adverse events secondary to other events 66](#_Toc13166)

[8.8.4 Persistent or recurrent adverse events 66](#_Toc12359)

[8.8.5 Abnormal laboratory test values 67](#_Toc4141)

[8.8.6 Abnormal vital signs 67](#_Toc1217)

[8.8.7 Abnormal liver function test results 68](#_Toc1796)

[8.8.8 Death 68](#_Toc26390)

[8.8.9 Pre-existing disease conditions 69](#_Toc18623)

[8.8.10 Adverse events related to drug overdose or administration errors 69](#_Toc15062)

[9 Data Collection and Management 70](#_Toc2379)

[9.1 Data Confidentiality 70](#_Toc12781)

[9.2 Central Monitoring 70](#_Toc13687)

[9.3 Data Collection 70](#_Toc17974)

[9.4 Database Management and Quality Control 70](#_Toc18132)

[9.5 Database Locking 71](#_Toc23963)

[10 Statistical Analysis 72](#_Toc31699)

[10.1 Data Analysis Set 72](#_Toc13358)

[10.2 Demographic and Baseline Disease Characteristics 72](#_Toc25091)

[10.3 Efficacy Analysis 73](#_Toc25601)

[10.3.1 Primary Efficacy Endpoints 73](#_Toc19201)

[10.3.2 Secondary Efficacy Endpoints 73](#_Toc24776)

[10.4 Safety Analysis 73](#_Toc18961)

[11 Research Management 74](#_Toc11660)

[11.1 Ethical Considerations 74](#_Toc1928)

[11.2 Informed Consent 74](#_Toc1274)

[11.3 Compensation for Harm to Subjects 74](#_Toc24679)

[11.4 Preservation of Research Documents, Records, and Files 75](#_Toc12554)

[11.5 Confidentiality of Research Documents and Patient Records 75](#_Toc15312)

[11.6 Return or Destruction of Study Drugs/Treatment Supplies 75](#_Toc29980)

[11.7 Monitoring and Auditing 7](#_Toc3402)5

[11.8 Protocol Deviation 76](#_Toc17852)

[11.9 Study Summary Report 76](#_Toc29157)

[12 References 77](#_Toc9119)

[13 Appendices 79](#_Toc1981)

[Appendix 1 Trial Flowchart 79](#_Toc23468)

[14 Appendix 84](#_Toc23160)

[Appendix One Common Toxicity Criteria (CTC) Version 5.0 by the National Cancer Institute (NCI) 84](#_Toc15357)

[Appendix Two Criteria for Evaluation of Solid Tumor Efficacy 85](#_Toc21667)

[Appendix Three: Modified Criteria for Evaluation of Solid Tumor Efficacy 100](#_Toc3648)

[Appendix IV: Child-Pugh Classification for Liver Function 111](#_Toc25895)

[Appendix V: Eastern Cooperative Oncology Group (ECOG) Performance Status 112](#_Toc27116)

[Appendix VI: Classification of Portal Vein Tumor Thrombus (PVTT) 113](#_Toc16677)

[Appendix VII: Management Measures for Allergic Reactions 114](#_Toc17680)

[Appendix VIII: Recommendations for Replacement Therapy in Hypothyroidism 115](#_Toc11774)

# Research Background

## Hepatocellular Carcinoma

Hepatocellular carcinoma (HCC) is one of the most common malignant tumors clinically worldwide, with over 700,000 new cases reported annually, ranking fifth among malignant tumors; Approximately 680,000 deaths per year, ranking third in cancer-related deaths, only behind lung cancer and gastric cancer ^[1]^. Globally, the incidence of hepatocellular carcinoma is also increasing year by year. 90% of primary liver cancer cases are hepatocellular carcinoma, which is a cancer with distinctive characteristics in China. In our country, liver cancer ranks 5th in incidence and 2nd in mortality among tumors, accounting for 55% of the global incidence and 45% of the global mortality each year. China is the country with the highest incidence and mortality of liver cancer in the world ^[2]^. Hepatocellular carcinoma is one of the malignant tumors that severely threaten the life and health of the people in our country.

Currently, surgical resection remains one of the most effective treatment methods for hepatocellular carcinoma. However, due to the lack of typical clinical manifestations in early-stage liver cancer, once symptoms and signs appear, the disease has often progressed to the middle or late stages. Most patients are unable to undergo radical surgical resection due to reasons such as high tumor burden, tumor metastasis, or the patient's physical condition or cardiopulmonary function being unable to withstand major traumatic surgery. Only 20% to 30% of patients meet the requirements for surgical treatment, and the recurrence rate of tumors after liver cancer resection is still very high, with a treatment effect that is not satisfactory; the 5-year recurrence rate reaches 60% to 70%, or even higher ^[3,4]^. Due to the complexity of tumor biological behavior in liver cancer and the limitations of various single treatment methods, the current consensus in the field of liver cancer treatment is a multidisciplinary collaboration and the coexistence of multiple treatment methods, which often involves a comprehensive approach in clinical practice that combines intervention, ablation, targeted therapy, immunotherapy, and radiochemotherapy to control tumor progression.

## Hepatic Arterial Infusion Chemotherapy (HAIC)

The liver receives dual blood supply from the hepatic artery and the portal vein, with 70% of the normal liver's blood supply coming from the portal vein and the remaining 30% from the hepatic artery, while the blood supply to liver cancer is primarily provided by the hepatic artery. Therefore, by administering through the hepatic artery, it is possible to maintain continuous exposure of the tumor to high concentrations of chemotherapeutic agents, maximizing the destruction of tumor cells while minimizing the impact on normal liver tissue. Additionally, due to the first-pass effect of the liver, the systemic dose of most chemotherapeutic agents is relatively low, resulting in reduced systemic toxic side effects. Hepatic Arterial Infusion Chemotherapy (HAIC) involves puncturing the right femoral artery (or other arteries such as the radial artery, subclavian artery, etc.), inserting a catheter, and performing angiography of the celiac trunk and mesenteric artery. Then, based on the arterial blood supply to the tumor, the catheter is selectively placed into the artery supplying the tumor. If the tumor receives blood supply from both the celiac trunk and the superior mesenteric artery simultaneously, or has other sources of supplying arteries, a microcatheter will be placed into the largest supplying artery of the tumor. Subsequently, chemotherapeutic agents will be pumped into the liver and tumor tissue for a prolonged and stable duration through the hepatic artery catheter. HAIC is routinely repeated every 3 weeks, with each session involving re-angiography, catheterization, and fixation; if there are changes in the tumor's blood supply, the catheter should be repositioned in the supplying vessel of the tumor each time. As one of the treatment strategies for interventional therapy, HAIC has undergone 30 years of development and is now commonly performed using the FOLFOX regimen for chemotherapy, with the technical aspects having become very mature ^[5-7]^.

Compared with TACE, HAIC can sustain administration for several days, significantly increasing the total dose of chemotherapeutic agents, prolonging the action time of high-concentration chemotherapeutic drugs, and avoiding the use of any embolic agents, thereby eliminating the occurrence of adverse events such as embolic syndrome and ectopic embolism, demonstrating better safety and efficacy, especially suitable for patients with portal vein tumor thrombus and large liver cancer, providing unresectable liver cancer patients with a higher objective tumor response rate and surgical conversion rate ^[8]^.

## Donafenib tablets (referred to as donafenib)

Tyrosine kinase-mediated signaling is directly related to tumor occurrence and development. The development of drugs targeting this pathway has become a current hotspot in antitumor therapy. The successive successful market launch of various small molecule tyrosine kinase inhibitors (TKIs) strongly supports the antitumor pathway targeting tyrosine kinases. Receptor tyrosine kinase inhibitors include both single-target and multi-target TKIs. The early marketed protein kinase inhibitors were mainly specific inhibitors targeting a single target. Although they achieved remarkable success in tumor treatment upon their initial launch, the cellular signaling process is extremely complex. Attempting to block all signaling pathways of tumor cells through single-target drugs may not yield objective efficacy and could potentially lead to resistance, thus giving rise to the concept of multi-target drugs. Currently, research on multi-target receptor tyrosine kinase inhibitors is very active, among which sorafenib (abbreviated as: sorafenib) and malate sunitinib (abbreviated as: sunitinib) have been approved for marketing in the United States, becoming representatives of broad-spectrum TKIs.

Donafenib tablets are an oral multi-target multi-kinase inhibitor class of small molecule anti-tumor drugs developed by Suzhou Zelgen Biopharmaceutical Co., Ltd. Donafenib employs a novel and unique drug chemistry technique, forming a new structure by replacing a methyl group on the sorafenib molecule with a tri-deuterated methyl group. The deuteration rate is not less than 99.0%, and the deuterated compounds retain the basic physicochemical properties of the parent drug. At the same time, its metabolic stability may enhance pharmacological effects and will not lead to new toxic target organs and toxic effects caused by traditional chemical modifications. Donafenib has multiple targets and dual anti-tumor effects; it can directly inhibit the proliferation and growth of tumor cells by blocking the cell signaling pathway mediated by serine-threonine kinase (Raf/MEK/ERK), and it can also indirectly inhibit tumor cell growth by blocking the formation of new blood vessels in tumors through the inhibition of the activity of various tyrosine kinases such as vascular endothelial growth factor receptor (VEGFR) and platelet-derived growth factor receptor (PDGFR), becoming the first domestically produced targeted new drug for first-line treatment of advanced liver cancer to be marketed in China. In addition, Donafenib can also improve the tumor immune microenvironment through various mechanisms. Therefore, the combination of PD-1/PD-L1 antibodies with donafenib not only produces an additive effect but can also create a synergistic effect where 1+1>2, enhancing efficacy. For details, see the donafenib investigator's brochure (IB).

### 1.3.1 Preclinical studies of donafenib

#### 1.3.3.1 Pharmacokinetics

Beagle dogs were simultaneously administered 5.0 mg/kg of sorafenib and donafenib raw material via gavage [50% polyethylene glycol (PEG) 400/50% propylene glycol formulation], and both exhibited similar pharmacokinetic (PK) characteristics in beagle dogs, with peak plasma concentration time (T _max_ ) of approximately 1.7 hours; The peak concentration (C _max_ ) and AUC _0-t_ of donafenib are 101.9% and 112.5% of those of sorafenib, respectively, and the elimination half-lives (t _1/2_ ) of sorafenib and donafenib are 4.08 and 4.29 hours, respectively, with donafenib being 105% of sorafenib.

#### 1.3.3.2 Toxicology

1.3.3.2.1 Acute toxicity test

Wistar rats were administered a single oral dose of donafenib, with a maximum tolerated dose (MTD) greater than 2000 mg/kg. Beagle dogs were administered a single oral dose of donafenib, with an MTD greater than 1000 mg/kg;

1.3.3.2.2 Long-term toxicity study

Wistar rats were administered donafenib by oral gavage for 4 weeks. The target organs/systems for donafenib toxicity were the kidneys, hematopoietic system, immune system, digestive system, endocrine system (adrenal gland, thyroid), reproductive system, vasculature, and skeleton. No adverse reaction dose levels (NOAEL) were observed below 3 mg/kg, with the maximum tolerated dose (MTD) being 10 mg/kg. Beagle dogs were administered donafenib by oral gavage for 4 weeks. The target organs/systems for donafenib toxicity were the liver, adrenal gland, reproductive system (testes, epididymis, ovaries, uterus), and mesenteric lymph nodes, and it could cause damage to teeth and gums. Moreover, this trial failed to identify the NOAEL for donafenib , with the highest no severe toxicity dose (HNSTD) being 10 mg/kg.

The results of the 6-month long-term toxicity study showed that Wistar rats administered donafenib via oral gavage for 6 months had the toxicity target organs/systems including the kidneys, hematopoietic system, skeleton and teeth, adrenal glands, and male reproductive system (testes, epididymis). The HNSTD was 1.0 mg/kg. Beagle dogs administered donafenib via oral gavage for 6 months had the toxicity target organs including the liver, skin, teeth, and thymus, and could also induce anemia in the body. The HNSTD was 3 mg/kg;

1.3.3.2.3 Fertility and Early Embryonic Development Toxicity Test

Wistar rats were administered donafenib via oral gavage, with the NOAEL for male fertility being 3.0 mg/kg, and the NOAEL for female fertility and early embryonic development being 0.8 mg/kg;

New Zealand rabbits were administered toluenesulfonic acid donafenib via oral gavage, with the NOAEL for parental pregnant rabbits being less than 0.15 mg/kg; the NOAEL for embryos and fetuses was 0.15 mg/kg.

1.3.3.2.4 Mutagenicity Test

The Ames test result for donafenib was negative. The in vitro CHL cell chromosome aberration test result for donafenib was positive. The mouse bone marrow micronucleus test result was negative.

Detailed preclinical study results of donafenib can be found in the donafenib investigator's brochure.

### 1.3.2 Clinical Research of Donafenib

#### 1.3.2.1 Study TG1219DTT

The study TG1219DTT has been completed, with the objective of observing the safety and tolerability characteristics of different oral doses of donafenib tablets in humans, as well as monitoring the potential occurrence of DLT and maximum tolerated dose (MTD), pharmacokinetic parameters in the set dose groups, and providing a basis for the dosing regimen in the Phase II clinical trial. The trial includes three parts: a dose-escalation tolerance study, a pharmacokinetics study, and a study on the effect of food on drug metabolism. A total of 25 subjects with advanced malignant solid tumors were enrolled. Three patients in the 50 mg group participated only in the single-dose tolerance trial. In the single/multiple dose study, 22 patients participated, with sample sizes for each dose group being 3 for the 0.1 g group, 6 for the 0.2 g group, 7 for the 0.3 g group, and 6 for the 0.4 g group. Among the 22 patients in the single/multiple dose study group, 4 patients did not complete the planned 28-day dosing period due to adverse events, including 1 in the 0.2 g group, 2 in the 0.3 g group, and 1 in the 0.4 g group.

Safety study results: A total of 5 patients experienced DLT in this study, including 1 case in the 0.2g dose group, 1 case in the 0.3g dose group, and 3 cases in the 0.4g dose group. During the study period of single and multiple administrations, a total of 20 cases (80.0%) experienced adverse reactions (ADR). The incidence of ADR during the single administration phase was 36.0% (9/25), while the incidence of ADR during the multiple administration phase was 86.4% (19/22). Ranked by incidence, the most common adverse reactions were hand-foot syndrome (12 cases, 48.0%), diarrhea (8 cases, 32.0%), rash (6 cases, 24.0%), alopecia (5 cases, 20.0%), nail pigmentation (5 cases, 20.0%), and hypertension (5 cases, 20.0%). According to the classification by system organ, the 'gastrointestinal system' is the most common adverse reaction, with a total of 16 out of 25 patients (64.0%) experiencing 'gastrointestinal system' related adverse reactions. The main manifestations include diarrhea (8 cases, 32.0%), stomatitis (4 cases, 16.0%), reduced food intake (3 cases, 12.0%), nausea (2 cases, 8.0%), abdominal distension (2 cases, 8.0%), constipation (2 cases, 8.0%), and abdominal pain (2 cases, 8.0%). Fifteen patients (60.0%) experienced 'skin and subcutaneous tissue' related adverse reactions. The main 'skin and subcutaneous tissue' adverse reactions were hand-foot syndrome (12 cases, 48.0%), rash (6 cases, 24.0%), alopecia (5 cases, 20.0%), and nail pigmentation (5 cases, 20.0%), among others.

PK studies indicate that a single administration of donafenib , within the dose range of 0.1 to 0.4 g, results in an increase in the AUC _0-t_ and C _max_ of the parent drug in subjects as the administration dose increases, with the increase of the parent drug being generally consistent with the proportional increase in dose; the PK parameters of donafenib exhibit considerable inter-subject variability. donafenib was administered continuously, twice daily (Bid), for 28 days. During days 7 to 14 of administration, the plasma concentrations of donafenib and its metabolites reached a steady state. The ratio of the AUC _0-12h_ of donafenib on day 28 to that on day 1 (accumulation ratio) was 2.94 to 7.75, indicating a degree of accumulation. Within the dose range of 0.1 to 0.4 g, the AUC _0-12h,ss_ and C _max_ of donafenib did not show significant changes with increasing doses. A high-fat diet had little effect on the PK parameters of donafenib .

21 subjects completed at least one efficacy evaluation [according to the Response Evaluation Criteria in Solid Tumors (RECIST) v1.1]. 13 subjects entered the extended treatment period. There were 2 cases of subjects (9.5%) who achieved partial response (PR) [1 case of colorectal adenocarcinoma (0.3 g group), and 1 case of hepatocellular carcinoma (0.2 g group)], 10 cases of stable disease (47.6%) [3 cases of liver cancer (1 case each from the 0.1, 0.3, and 0.4 g groups), 4 cases of lung cancer (2 cases from the 0.2 g group, and 1 case each from the 0.1 g and 0.4 g groups), 2 cases of renal cancer (1 case each from the 0.1 g and 0.2 g groups), and 1 case of colorectal cancer (0.4 g group)], and 9 cases of disease progression (42.9%). Additionally, the tumor tissues of the tumor subjects exhibited significant cavitary necrosis, indicating that donafenib has a notable anti-angiogenic effect. Among the subjects in the extended trial period, the longest duration of medication was 17.8 months, the shortest was 3.0 months, and the average duration of medication was 7.9 months, with a 95% confidence interval (CI) of (5.0, 10.9). Among them, 3 cases (2 cases of renal cancer and 1 case of hepatocellular carcinoma) had a duration exceeding 12 months.

#### 1.3.2.2 Study ZGDH1B

Study ZGDH1B has been completed, with the primary objective of observing the safety and tolerability of donafenib tablets for liver cancer treatment at a clinically recommended dose in a larger sample. The study employed a randomized, open-label, parallel-group controlled design. A total of 106 subjects were planned for enrollment, with 107 subjects actually enrolled. The trial is designed with two groups: the donafenib tablet 0.2g group, with a total of 53 subjects; and the donafenib tablet 0.3g group, with a total of 54 subjects. Subjects will take the medication on an empty stomach, twice daily, with each treatment cycle lasting 4 weeks (28 days), until disease progression or intolerability occurs. Follow-up will occur once every cycle (4 weeks), and imaging efficacy evaluations will be conducted every two cycles (8 weeks).

Donafenib tablets demonstrated preliminary efficacy, with an objective response rate (ORR) of 7.8% (4 cases of PR) in the donafenib 0.2 g group, and a 95% confidence interval (CI) of 2.0% to 19.0%; The ORR for the 0.3 g group was 5.7% (3 cases of PR), with a 95% CI of 1.0% to 16.0%. In the 0.2 g group, a total of 33 cases (64.7%) experienced tumor progression, with a median time of 3.8 months (3.61-5.65); In the 0.3 g group, a total of 39 cases (73.6%) experienced tumor progression, with a median time of 3.7 months (2.04-5.52). The median overall survival in the 0.2 g group was 12.2 months (8.11-14.00); The median overall survival in the 0.3 g group was 10.3 months (7.10-15.90).

In terms of PK, the 0.2 g group received Bid continuous administration for 28 days, with a peak blood concentration of donafenib reached at 2.00 hours (median), C_max_ was 6.81 µg/mL, C_min_ was 2.20 µg/mL, and the plasma exposure AUCss was 46.7 h*µg/mL. C_max_ is 4.74 times that of Day 1 administration, and AUCss is 4.99 times that of Day 1 administration. In the 0.3g donafenib group, Bid continuous administration for 28 days resulted in a peak plasma concentration (median) of 9.13µg/mL at 2.00 hours, with C_max_ of 9.13µg/mL, C_min_ of 2.69µg/mL, and plasma exposure AUCss of 58.9h*µg/mL. C_max_ is 2.96 times that of Day 1 administration, and AUCss is 3.10 times that of Day 1 administration.

Safety profile, the incidence of TEAE in the 0.2g group is 96.2%, and in the 0.3g group is 98.1%; The incidence of SAE in the 0.2g group is 39.6%, and in the 0.3g group is 22.2%; The incidence of TEAE leading to death in the 0.2g group was 15.1%, and in the 0.3g group was 13.0%; The most frequently occurring SOC of TEAE in both groups was 'Skin and Subcutaneous Tissue Disorders' (incidence in the 0.2g group was 75.5%, and in the 0.3g group was 83.3%), with the PT being Palmoplantar Erythrodysesthesia Syndrome (incidence in the 0.2g group was 62.3%, and in the 0.3g group was 61.1%). The overall safety and tolerability of donafenib 0.2g and 0.3g were good.

**1.3.2.3 Study ZGDH3**

The open, randomized, parallel-controlled, multicenter phase II/III clinical study (ZGDH3) of donafenib as first-line treatment for advanced hepatocellular carcinoma included a total of 668 unresectable advanced HCC patients, randomized 1:1, receiving donafenib 0.2g BID or Sorafenib 0.4g BID treatment. The results showed that the OS in the donafenib group was significantly prolonged compared to the Sorafenib group (12.1 months vs 10.3 months; HR=0.831; 95% CI, 0.699~0.988). donafenib demonstrated better tolerability compared to Sorafenib: the incidence of ≥3 grade adverse events (AE) (57.4% vs 67.5%, *p* =0.0082), ≥3 grade drug adverse reactions (ADR) (37.5% vs 49.7%, *p* =0.0018), and the incidence of ADR leading to drug suspension and dose reduction (30.3% vs 42.5%, *p* =0.0013) were all significantly lower than those of Sorafenib; The incidence and severity of hand-foot skin reactions, liver function abnormalities, and diarrhea were all lower than those in the sorafenib group. In the treatment with a single anti-angiogenic targeted drug, donafenib is the only targeted drug to date that has shown superior overall survival (OS) compared to sorafenib in head-to-head studies for advanced HCC ^[10]^.

**1.4 Recombinant humanized anti PD-1/PD-L1 monoclonal antibody**

PD-1 is an immunosuppressive receptor primarily expressed on the surface of T cells, B cells, monocytes, and NK cells, with its corresponding ligands being programmed death ligand-1 (PD-L1) and programmed death ligand-2 (PD-L2). Among them, PD-L1 is widely expressed in various tumor cells and immune cells, and its expression level can be upregulated under the action of various cytokines such as interferon (IFN)-γ in the tumor microenvironment. The PD-L1 on the surface of tumor cells and antigen-presenting cells binds to PD-1, continuously activating the PD-1 pathway, which can inhibit tumor antigen-specific T cell activation and weaken T cell anti-tumor activity. PD-1/PD-L1 antibodies interfere with the binding of PD-1 to PD-L1, relieving the immune suppression of this pathway and restoring T cell anti-tumor immunity ^[9]^.

The durability of the efficacy of PD-1 inhibitors is due to the memory function of the immune system. Therefore, once PD-1 inhibitors take effect, some patients achieve clinical cure, with no recurrence or progression for an extended period, leading to long-term survival ^[11]^.

As of now, the FDA has approved two PD-1 targeted immune inhibitors for second-line treatment of advanced liver cancer (namely, Nivolumab and Pembrolizumab), and one PD-L1 targeted immune inhibitor in combination with Bevacizumab for first-line treatment of advanced liver cancer (Atezolizumab). In China, Nivolumab, Pembrolizumab, and Atezolizumab have also successively received marketing approval. Among the three major domestic anti-PD-1 drugs, Sintilimab and Toripalimab have also been approved for marketing in China, with Sintilimab having been approved by the NMPA for use in combination with Bevacizumab for first-line treatment of HCC.

**1.5 Research Basis**

Liver cancer is a hypervascular solid tumor, and angiogenesis plays an important role in its growth, development, and prognosis. Angiogenic factors such as VEGF can promote tumor angiogenesis, while the disordered neovascular system can form a physical barrier, causing recruitment and infiltration obstacles for T cells; On the other hand, VEGF can induce tumor-associated immune suppression through various mechanisms, such as inhibiting the maturation of dendritic cells and promoting the activity of Tregs, MDSCs, and suppressing CTLs ^[12]^. Therefore, drugs that antagonize VEGF have a positive enhancing effect on immune responses. Moreover, the activation of immune cells can, in turn, affect tumor blood vessels, leading to their normalization. The two processes coordinate with each other, creating a mutually enhancing positive feedback loop by improving the tumor microenvironment. In addition, targeted therapies have advantages such as high specificity, rapid onset, a relatively high overall response rate, and minimal damage to normal tissues; however, the emergence of resistance limits the duration of the antitumor efficacy. Although immunotherapy has a slow onset, the duration of relief is prolonged due to the generation of memory cells ^[13]^. Tumor cell death induced by targeted drugs can enhance anti-tumor immune responses by releasing neoantigens. These provide a solid theoretical basis for the synergistic anti-tumor effects of immune checkpoint inhibitors combined with targeted drugs.

In the field of liver cancer, combination therapies of anti-angiogenic drugs and immunotherapy have shown significant survival improvement and good tolerability in advanced unresectable HCC. Currently, the combination regimen of Atezolizumab + Bevacizumab has been approved by the NMPA and FDA for first-line treatment of unresectable HCC.

Many liver cancer patients with poor HAIC outcomes often have extrahepatic metastasis and progression. Therefore, a combination treatment at this time should be a more reasonable choice. By relying on HAIC combined with systemic therapy, it can both eliminate local tumor burden and increase the concentration of antitumor drugs in the systemic circulation to delay extrahepatic progression, thereby comprehensively improving treatment efficacy.

Recently, the 2021 ASCO meeting reported a triplet therapy of Toripalimab combined with Lenvatinib and HAIC for first-line treatment of advanced hepatocellular carcinoma (NCT04044313). This study is a prospective single-arm Phase II trial that included 36 patients with advanced liver cancer who had not received systemic treatment, receiving lenvatinib in combination with toripalimab and HAIC treatment. The study results showed that after a median follow-up of 11.2 months, the median progression-free survival was 10.5 months, and the median overall survival has not yet been reached. The objective response rate (ORR) assessed according to RECIST criteria was 63.9%, and the ORR assessed according to mRECIST criteria was 66.7%, with 5 patients achieving radiological complete response. The median duration of response was 12.1 months. In addition, 8 patients were downstaged to resectable liver cancer, of which 1 underwent liver transplantation and 4 underwent curative surgical resection, with one achieving a pathological complete response. 72.2% of patients experienced grade 3-4 treatment-related adverse events (AEs), the most common being thrombocytopenia (13.9%), elevated aspartate aminotransferase (13.9%), and hypertension (11.1%) ^[14]^. Additionally, a retrospective study on lenvatinib combined with toripalimab and HAIC for first-line treatment of advanced hepatocellular carcinoma showed (Abstract e16124) that among 34 patients receiving treatment, the ORR was 84.0% (RECIST 1.1), with a CR rate of 8%. The incidence of grade 3-4 TRAEs was 28.0%. It can be seen that the triple therapy has significant therapeutic effects, and the adverse events caused by the treatment are acceptable, which may become the trend for future treatment of advanced liver cancer ^[15]^.

**1.6 Benefit-Risk Assessment**

The target population enrolled in this study consists of patients with unresectable intermediate to advanced hepatocellular carcinoma. For this type of patient, current single treatment methods have certain limitations and are difficult to meet clinical treatment needs; combination therapy is the direction for future development and breakthroughs.

Both donafenib and Sintilimab have been approved by the NMPA for the treatment of HCC. The treatment of HAIC has undergone long-term development, accumulating a wealth of clinical experience, and the technical aspects have become very mature. The combination of the three may synergistically enhance efficacy, providing greater clinical benefits to patients.

Additionally, this study will develop a detailed risk management plan to ensure that the potential harms of anticipated adverse events (AEs) to subjects are minimized. At the same time, during the treatment with the investigational drug, adverse events (AEs) and immune-related adverse events (irAEs) experienced by subjects will be closely monitored, and once relevant adverse reactions occur, the study physician will take immediate corresponding measures to ensure the safety of the subjects.

# 2 Research Objectives

## 2.1 Primary Objective

To evaluate the efficacy of donafenib combined with Hepatic Arterial Infusion Chemotherapy (HAIC) and Sintilimab as first-line treatment for unresectable hepatocellular carcinoma based on the objective response rate (ORR) (tumor assessment will be conducted using both RECIST v1.1 and mRECIST criteria, with RECIST v1.1 as the primary standard).

**2.2 Secondary Objectives**

To evaluate the efficacy and safety of donafenib combined with Hepatic Arterial Infusion Chemotherapy (HAIC) and Sintilimab as first-line treatment for unresectable hepatocellular carcinoma based on overall survival (OS), time to progression (TTP), progression-free survival (PFS), disease control rate (DCR), duration of response (DOR), changes in ECOG PS scores over time, changes in AFP levels over time, as well as adverse events and serious adverse events (tumor assessment will be conducted using both RECIST v1.1 and mRECIST criteria, with RECIST v1.1 as the primary standard).

# Research design

## Overview of the research design

This study is a single-center, open-label, single-arm, exploratory study, planning to enroll 30 cases of unresectable advanced hepatocellular carcinoma patients (see sections 4.1 and 4.2 for details).

## Sample size calculation basis

This study is an exploratory study, using a fixed sample size of 30 cases.

## Dose selection basis

### 3.3.1 Donafenib tablets

In this study, the dose of donafenib is 200mg Bid. 200mg Bid is the standard dosing regimen for donafenib in phase III clinical trials for first-line treatment of advanced hepatocellular carcinoma. An exploratory study of 6 patients with advanced gastrointestinal tumors (including 4 cases of liver cancer) treated with 100 mg Bid of donafenib combined with 240 mg Q2W of Sintilimab showed that the overall safety profile of the combination therapy was similar to that of either Sintilimab or donafenib monotherapy, with no unexpected adverse reactions occurring (data not yet published).

This study includes a safety lead-in phase, where the first 6 subjects enrolled in the study will undergo gradual safety monitoring. Each patient will receive oral administration of donafenib tablets [200 mg, Bid] combined with intravenous infusion of Sintilimab injection [200 mg, Q3W], and will receive HAIC treatment on the same day or the following day after Sintilimab infusion. The monitoring window is from the first administration of donafenib treatment to 21±3 days after the first HAIC treatment (i.e., from D1 of the donafenib monotherapy period to C2D1 of the combination therapy period). If ≥2 cases of dose-limiting toxicity (DLT) are observed among the first 6 subjects, and the research team assesses that the toxicity is solely related to donafenib treatment, the initial dose for subsequent enrolled patients receiving donafenib treatment will be adjusted to 100 mg Bid.

### 3.3.2 Sintilimab Injection

Administer according to the administration method and dosage in the Sintilimab instructions, 200 mg, intravenous infusion, Q3W.

### 3.3.3 Hepatic Arterial Infusion Chemotherapy (HAIC)

Based on the clinical routine experience of this research center, the FOLFOX regimen was selected for HAIC, with the following doses: Oxaliplatin 85 mg/m^2^ infusion for 2 hours, Leucovorin 400 mg/m^2^ infusion for 2 hours, 5-FU 400 mg/m^2^ bolus for 10 minutes, followed by continuous infusion of 5-FU 1200 mg/m^2^ for 23 hours. The first HAIC will be implemented on the date predetermined by the investigator, followed by once every 3 weeks (Q3W). The total number of HAIC treatments will be determined by the investigator based on the actual condition of the patient as needed.

## 3.4 DLT Assessment and Judgment Criteria

DLT is defined as any of the following adverse events, unless the investigator determines that it is definitely related to the study progression or caused by other external factors, it will be considered as DLT:

Hematologic Toxicity:

- - - 1. Grade 4 neutropenia lasting more than 7 days.
      2. Febrile neutropenia [defined as an absolute neutrophil count (ANC) < 1×10^9^ /L, accompanied by a single temperature reaching 38.3℃ or a temperature of 38℃ persisting for more than one hour].
      3. Grade 3 neutropenia with infection.
      4. Grade 3 thrombocytopenia with severe uncontrollable bleeding events.
      5. Grade 4 thrombocytopenia.
      6. Grade 4 anemia (life-threatening).
      7. Grade 3 or 4 lymphopenia with opportunistic infections.

Non-hematologic toxicity:

Skin reactions: severe hand-foot skin reaction (HFSR) or other skin adverse reactions that persist for >2 weeks or occur a second time despite appropriate intervention.

Blood pressure: persistent/recurrent systolic blood pressure ≥160 mmHg or diastolic pressure ≥100 mmHg on the second occurrence, and remains uncontrolled despite aggressive antihypertensive treatment. (Persistent/recurrent blood pressure ≥160/100 mmHg refers to measurements of ≥160/100 mmHg taken at least twice with an interval of 24 hours, with each measurement taken after the subject has been seated and resting quietly for more than 5 minutes.) At least 1 week of antihypertensive medication is allowed before the second recorded hypertension event occurs.

Cardiac contractile function: First occurrence of LVEF decrease >10%, or LVEF value <40%.

Gastrointestinal system: Grade 4 diarrhea, or Grade 3 diarrhea lasting >24 hours despite active antidiarrheal treatment, or occurrence of two episodes of Grade 3 diarrhea regardless of duration; Occurrence of ≥ grade 3 nausea and vomiting despite adequate/maximum medical intervention and/or preventive measures.

Absolute QTcB ≥500 msec (at two consecutive time points).

Other Grade 4 or higher adverse events.

Grade 3 or higher immune-related adverse events (irAE).

Other Grade 3 toxic effects, except for laboratory abnormalities that recover to Grade 2 (inclusive) or below within 3 days (asymptomatic and not requiring medical intervention).

In addition to the above events, DLT events also include any level of toxic reaction that the investigator considers necessitating the subject's withdrawal from the study.

# Study Population

## Inclusion criteria

Patients who meet all of the following criteria may be included in this study:

1. Voluntary enrollment with signed written informed consent;
2. Aged 18 to 80 years (inclusive), regardless of gender;
3. Patients with hepatocellular carcinoma clinically diagnosed according to the 'Guidelines for the Diagnosis and Treatment of Primary Liver Cancer (2019 Edition)' or confirmed by histological/cytological diagnosis;
4. Patients with unresectable or metastatic hepatocellular carcinoma;
5. No prior systemic treatment. Patients who have received adjuvant chemotherapy after local treatment may also be included if the chemotherapy ended more than 12 months ago and there has been disease progression or metastasis;
6. The last intervention, radiotherapy, and ablation treatment must have ended more than 4 weeks ago;
7. Patients who have previously undergone liver resection should have R0 resection, and tumor recurrence should occur more than 24 months after surgery;
8. There must be at least one assessable lesion (according to RECIST 1.1 criteria) ;
9. Expected survival time ≥ 3 months;
10. Eastern Cooperative Oncology Group (ECOG) performance status (PS) score of 0-1;
11. Child-Pugh score ≤ 7;
12. Able to cooperate in observing adverse events and efficacy;
13. Major organ functions are normal, meeting the following criteria:

**Complete blood count (no blood transfusion or G-CSF use within 14 days prior to screening):**

1. Hemoglobin ≥ 90 g/L;
2. Absolute Neutrophil Count (ANC) ≥ 1.5×10^9^ /L;
3. Platelet count ≥ 75 × 10^9^ /L;

**Biochemical tests (no albumin use within 14 days prior to screening):**

1. Albumin ≥ 28 g/L;
2. Total bilirubin ≤ 2 × upper limit of normal (ULN);
3. Aspartate aminotransferase (AST), alanine aminotransferase (ALT) ≤ 5 × ULN;

g) Alkaline Phosphatase (ALP) ≤ 5×ULN;

h) Creatinine ≤ 1.5×ULN;

**Coagulation function:**

1. International normalized ratio (INR) or prothrombin time (PT) ≤1.5×ULN;

J) Activated Partial Thromboplastin Time (APTT) ≤ 1.5×ULN.

## Exclusion criteria

Previous or concurrent diseases:

1. Histologically/cytologically confirmed previous diagnosis of fibrolamellar hepatocellular carcinoma, sarcomatoid hepatocellular carcinoma, cholangiocarcinoma, or other components;
2. History of malignancies other than hepatocellular carcinoma, unless meeting the following criteria:
   1. The patient has undergone potentially curative treatment and has no evidence of the disease for 5 years;
   2. Successfully received resection for basal cell carcinoma of the skin, squamous cell carcinoma of the skin, superficial bladder cancer, cervical carcinoma in situ, and other in situ cancers;
3. Diffuse tumor lesions;
4. Tumor vascular invasion presents one or more of the following conditions:

a) Involvement of the superior mesenteric vein;

b) Involvement of the inferior vena cava;

1. History of hepatic encephalopathy, hepatorenal syndrome, or history of liver transplantation;
2. Clinical symptoms requiring drainage of pleural effusion, ascites, or pericardial effusion;
3. Central nervous system metastasis;
4. History of severe mental illness;
5. Diseases affecting the absorption, distribution, metabolism, or clearance of the study drug (such as severe vomiting, chronic diarrhea, intestinal obstruction, malabsorption, etc.);

Previous or concomitant medications/treatments:

1. Previous receipt of allogeneic stem cell or solid organ transplantation;
2. Previous treatment with targeted therapies against VEGF and/or VEGFR, RAF, MEK signaling pathways such as sorafenib, lenvatinib, regorafenib, or immune modulators such as anti-PD-1, anti-PD-L1, anti-CTLA-4;
3. Patients who have previously received other systemic anti-tumor therapies, including traditional Chinese medicine with anti-tumor indications, within less than 2 weeks prior to the study medication, or those whose adverse events from previous treatments have not recovered to ≤ CTCAE Grade 1; Toxic reactions from prior cancer treatments do not include alopecia and Grade 1/2 neurotoxicity caused by oxaliplatin;
4. Concurrent use of medications that may prolong QTc and/or induce Torsades de Pointes (TdP), or medications that affect drug metabolism;
5. A history of or currently having congenital or acquired immunodeficiency diseases;
6. Active or previously recorded autoimmune diseases or inflammatory diseases (including but not limited to: autoimmune hepatitis, interstitial pneumonia, inflammatory bowel disease, systemic lupus erythematosus, vasculitis, uveitis, pituitary inflammation, hyperthyroidism or hypothyroidism, asthma requiring bronchodilator treatment, etc.), individuals with vitiligo or asthma that has completely resolved in childhood, and those who require no intervention in adulthood may be included;
7. Previous receipt of allogeneic stem cell or solid organ transplantation;
8. Patients who have used systemic immunosuppressive therapy within 2 weeks prior to enrollment, or are expected to require systemic immunosuppressive therapy during the study, except for the following situations:
9. Intranasal, inhaled, topical, or local injection (e.g., intra-articular injection) corticosteroids;
10. Doses not exceeding 10 mg/day of prednisone or other equivalent systemic corticosteroids;
11. Corticosteroids used prophylactically for hypersensitivity reactions;

Safety:

1. Patients with known or suspected allergy to donafenib or similar drugs, or a history of hypersensitivity reactions to chimeric or humanized antibodies or fusion proteins, or those allergic to the excipients of the investigational drug;
2. Active bleeding or coagulation dysfunction, with a tendency to bleed or currently undergoing thrombolysis, anticoagulation, or antiplatelet therapy;
3. History of thrombosis or thromboembolic events within the past 6 months, such as stroke and/or transient ischemic attack, deep vein thrombosis, pulmonary embolism, etc.;
4. History of esophageal or gastric variceal bleeding events due to portal hypertension within the past 6 months, or any life-threatening bleeding events occurring within the past 3 months;
5. Significant clinically meaningful cardiovascular diseases, including but not limited to acute myocardial infarction, severe/uncontrolled angina, or coronary artery bypass surgery within the past 6 months, congestive heart failure (NYHA classification >2), poorly controlled or requiring pacemaker treatment for arrhythmias, and hypertension that is not controlled by medication (systolic blood pressure ≥140 mmHg and/or diastolic blood pressure ≥90 mmHg);
6. Other significant clinical and laboratory abnormalities that the investigator believes affect the safety assessment, such as: uncontrolled diabetes, chronic kidney disease, grade II or higher peripheral neuropathy (CTCAE V5.0), thyroid dysfunction, etc.;
7. Severe infections that are active or poorly controlled clinically; Active infections, including:

a) Positive for human immunodeficiency virus (HIV) (HIV1/2 antibodies);

b) Active hepatitis B (positive HBsAg or HBV DNA > 2000 IU/ml and abnormal liver function);

c) Active hepatitis C (HCV antibody positive or HCV RNA ≥ 10^3^ copies/ml and liver function abnormal);

d) Active tuberculosis;

e) Other uncontrolled active infections (CTCAE V5.0 > grade 2);

1. Not yet recovered from surgery, such as having unhealed incisions or severe postoperative complications;
2. Pregnant or breastfeeding women, as well as female or male patients of childbearing potential who are unwilling or unable to take effective contraceptive measures.

## Criteria for Termination of Study

### Early Termination of the Study

The investigator may terminate this study at any time, with reasons for early termination including but not limited to:

1. The discovery of unexpected, significant, or unacceptable risks to subjects enrolled in the study during the study period;
2. The discovery of significant errors in the clinical research protocol during the study period, making it difficult to evaluate the investigational drug;
3. Request for termination by health/regulatory authorities.

### Subject Early Termination (Dropout or Withdrawal)

Subjects have the right to withdraw from the investigational drug treatment or to withdraw their informed consent to participate in the study at any stage of the study for any reason, without losing any benefits or facing any penalties.

The investigator may terminate a subject's investigational drug treatment at any stage of the study for the following reasons:

1. The first occurrence of radiologically confirmed disease progression (concurrently meeting both RECIST v1.1 and mRECIST criteria for radiological progression) or a rapid deterioration of liver function to Child-Pugh Class C after treatment, and the subject has not recovered to a level that allows for continued study treatment within 3 weeks as assessed by the investigator;
2. Death from any cause;
3. Occurrence of intolerable AE that does not resolve after dose adjustment or suspension (if the AE can be determined to be related to one of the investigational drugs, and the investigator believes that the subject would benefit from continuing treatment with the other investigational drug in combination with HAIC, then the subject may continue to receive the other investigational drug in combination with HAIC until the completion of study treatment or the emergence of other conditions requiring early withdrawal/termination of the study);
4. The use of drugs or other substances that may provoke toxicity or lead to bias in study results, or if the investigator determines that continued treatment is detrimental to the subject's health;
5. Clinical signs or laboratory test results suggest pregnancy;
6. Subject compliance serious adverse ;
7. Subject withdraws informed consent;
8. Occurrence of concomitant diseases that prohibit the continuation of the study, or the subject requires treatment excluded by the study protocol;
9. Development of another type of cancer, excluding non-melanoma skin cancers (such as basal cell carcinoma, squamous cell carcinoma), if deemed curable and not life-threatening by the investigator;
10. Initiation of other antitumor therapy before confirmed disease progression;
11. Loss to follow-up;
12. Any other reasons confirmed by the investigator.

### Early withdrawal of subjects

If a subject withdraws early from the investigational drug treatment, the reasons for withdrawal should be understood and recorded as much as possible. Additionally, arrangements should be made for the subject to undergo all examinations listed in Appendix 1 for the end-of-treatment visit, and to record all adverse events and concomitant medications within 30 days after the last administration or before starting new antitumor therapy (whichever occurs first).

Subjects who withdraw early from investigational drug treatment due to disease progression (with imaging progression meeting both RECIST v1.1 and mRECIST criteria), starting new antitumor therapy, or reasons other than pregnancy will continue to be followed for efficacy until disease progression occurs, new antitumor therapy is initiated, informed consent is withdrawn, loss to follow-up occurs, death, or the study is terminated early (whichever occurs first).

Subjects who discontinue investigational drug treatment due to disease progression (with imaging progression meeting both RECIST v1.1 and mRECIST criteria) or initiation of new antitumor therapy or pregnancy will continue to be followed for survival until the subject withdraws informed consent, is lost to follow-up, dies, or the study is terminated early (whichever occurs first).

If a subject refuses or is unable to return for visits, every effort should be made to contact the patient or an informed contact by phone to determine survival status.

## Exclusion Criteria

Before statistical analysis of the data, the principal investigator and the statistical unit will determine whether individual cases should be excluded. In the event of any of the following situations, the principal investigator should comprehensively assess whether to exclude the subject based on the extent of the subject's completion of the study and the reasons for early withdrawal, and provide relevant explanations. The CRF form should be retained for reference.

1. The subject violated important inclusion/exclusion criteria;
2. During the study period, the subject did not comply with the study protocol, such as not using the investigational drug, being unable to collect safety evaluation samples as required by the study protocol, or having no data at all;
3. During the study period, the investigator believes that the subject has other factors that prevent continued participation in the study, such as the concurrent use of other antitumor therapies during the study period, and actually terminates the subject's participation in the study.

# Investigational Drug

## Investigational Drug Information

**Donafenib tablets:**

Dosage form: Tablet

Specification: 100 mg/tablet

Ingredients: 4-(4-{3-[4-chloro-3-(trifluoromethyl)phenyl]-ureido}-phenoxy)-2-(N-1′,1′,1′-trideuteromethyl)pyridine-3-carboxamide salt

Storage conditions: Store sealed below 25°C

Shelf life: 36 months

Manufacturer: Suzhou Zelgen Biopharmaceutical Co., Ltd.

**Sintilimab injection:**

Dosage form: Injection

Specification: 10 ml: 100 mg, 1 vial/box

Component: Humanized anti-PD-1 monoclonal antibody

Storage conditions: Store and transport protected from light at 2-8℃

Shelf life: 24 months

Manufacturer: Innovent Biologics (Suzhou) Co., Ltd.

The preparation of the drugs used in the study complies with the requirements of the 'Good Manufacturing Practice for Pharmaceuticals'. The quality meets the standards for clinical research drugs.

## Treatment regimen

**Donafenib tablets:**

Subjects will start taking donafenib 3-7 days prior to receiving HAIC treatment, with a starting dose of 200 mg, twice daily (200 mg bid). Subjects will take the medication orally twice a day on each administration day, in the morning and evening, on an empty stomach (1 hour before meals or more than 2 hours after meals), with a 12±1 hour interval between doses, and continue taking it.

- - Bid administration on each dosing day, fasting in the morning and evening (1 hour before meals or 2 hours after meals), taken orally once, with an interval of approximately 12 hours between the two doses. Qd administration (occurring during dose adjustment), taken orally once in the morning on each dosing day while fasting (1 hour before meals or 2 hours after meals). It is acceptable for the medication to be taken within 1 hour before or after the scheduled dosing time.
  - If the subject vomits after taking the medication and expels the original drug or part of it, in principle, the medication should not be supplemented, and it should be recorded as a missed dose.
  - If the medication has not been taken 1 hour after the scheduled dosing time, there is no need to supplement the medication, and it should be recorded as a missed dose.
  - donafenib will be administered continuously until the subjects meet the criteria for study termination.

**Sintilimab injection:**

On days 0-1 prior to each HAIC treatment, subjects will receive intravenous infusion of Sintilimab at a dose of 200 mg, administered once every 3 weeks (Q3W), until the subjects meet the criteria for study termination.

**HAIC：**

Based on the clinical routine experience of this research center, the FOLFOX regimen will be selected for HAIC, with the following doses: Oxaliplatin 85 mg/m^2^ infused over 2 hours, Leucovorin 400 mg/m^2^ infused over 2 hours, 5-Fu 400 mg/m^2^ bolus over 10 minutes, followed by continuous infusion of 5-Fu 1200 mg/m ^2^ over 23 hours. The first HAIC will be implemented on the date predetermined by the investigator, followed by once every 3 weeks, and the number of subsequent HAIC treatments will be conducted as needed based on the actual condition of the patient as determined by the investigator.

## Dose adjustment/suspension of medication

**donafenib tablets:**

1) The initial dose adjustment scheme is 200 mg bid

During the treatment period, if the subject experiences grade 4 hematologic toxicity or grade 3 non-hematologic toxicity related to the investigational drug, the medication will be temporarily suspended (according to NCI CTCAE V5.0 standards), only allowing the subject to undergo a maximum of 2 dose adjustments. If grade 4 hematologic toxicity or grade 3 non-hematologic toxicity cannot return to normal or CTCAE grade 1 within 2 weeks after suspension, the medication will be permanently discontinued. (Grade 3 or higher adverse reactions do not include alopecia, uncontrolled nausea/vomiting, uncontrolled allergic reactions, and asymptomatic laboratory test abnormalities)

After the first suspension of medication, if the adverse reactions recover to ≤ Grade 1 within 1 week, continue taking the original dose; If recovery to ≤ Grade 1 occurs within 2 weeks of drug suspension, the dose is reduced to 200 mg qd to continue treatment; After dose adjustment, if drug suspension occurs again, and recovery to ≤ Grade 1 occurs within 2 weeks, the dose is reduced to 200 mg qod to continue treatment. If grade 4 hematologic toxicity or grade 3 non-hematologic toxicity occurs again (the 3rd occurrence), the drug will be permanently discontinued. That is Dose adjustment plan:

| Initial dose | First dose reduction | Second dose reduction |
| --- | --- | --- |
| 200 mg bid | 200 mg qd | 200 mg qod |

2) Dose adjustment scheme when the initial dose is adjusted to 100 mg bid due to DLT

If ≥2 cases of dose-limiting toxicity (DLT) are observed among the first 6 subjects, and the research team assesses that the toxicity is solely related to donafenib treatment, then the initial dose adjustment for subsequent enrolled patients receiving donafenib treatment will be 100 mg bid. At this time, the dose adjustment follows the same principles and processes as normal dose adjustments, with specific dose changes as follows:

| Initial dose | First dose reduction | Second dose reduction |
| --- | --- | --- |
| 100mg bid | 100mg qd | 100mg qod |

**Sintilimab injection:**

Throughout the study, dose adjustments for sintilimab are not permitted; the principles for pausing and permanently discontinuing sintilimab administration are outlined in Table 1.

If a subject experiences a grade 2 drug-related irAE or a grade 3 or higher other AE (refer to the ESMO guidelines for the management of toxicity related to immune checkpoint inhibitors), the subject must suspend administration, with a maximum delay of 7 days; subsequent administration schedules will be adjusted accordingly. If the administration is delayed for more than 7 days, it will be considered a missed dose, and the subject will receive the next cycle of administration according to the original schedule (calculated from the date of the first administration). If treatment-related adverse reactions have not recovered to grade 0-1 or baseline levels within 6 weeks after the last dose of Sintilimab, then Sintilimab should be permanently discontinued. If a subject discontinues the medication for more than 6 weeks and the investigator determines that the risks of continuing administration outweigh the benefits, they may permanently withdraw from the study treatment.

If a subject requires a gradual reduction in dosage of steroids due to an adverse event, the recombinant humanized anti-PD-1 monoclonal antibody may be discontinued for a longer period until the steroid tapering is completed or reduced to a prednisone dose of ≤10 mg/day (or equivalent dose). If the discontinuation exceeds 6 weeks (with the maximum discontinuation time for sintilimab due to steroid tapering not exceeding 12 weeks), the investigator may reassess the overall risk-benefit evaluation before resuming the use of the recombinant humanized anti-PD-1 monoclonal antibody.

Subjects may interrupt administration for reasons other than toxic reactions (such as surgical procedures), with the interruption not exceeding 6 weeks. The investigator may restart the use of recombinant humanized anti-PD-1 monoclonal antibody *based on an overall risk-benefit assessment.*

**Table 1 Sintilimab Dose Adjustment Scheme**

| Sintilimab-related adverse events | Severity | Dose adjustment |
| --- | --- | --- |
| Pneumonia | Grade 2 | Suspend administration ^a^ |
|  | Grade 3 or 4 or recurrent Grade 2 | Permanent discontinuation |
| Diarrhea/Colitis | Grade 2 or 3 | Suspend administration ^a^ |
|  | Grade 4 | Permanent discontinuation |
| Hepatitis | For subjects with baseline normal ALT, AST, or TBIL, a 2nd grade increase in AST, ALT (3-5 times ULN) or TBIL (1.5-3 times ULN); For subjects with baseline AST, ALT, or TBIL > ULN, an increase in AST, ALT, or TBIL ≥ 50% (meeting the 2nd grade requirement) and a duration of < 7 days. | Suspend administration ^a^ |
|  | For subjects with baseline normal ALT, AST, or TBIL, a 3rd or 4th grade increase in AST, ALT (> 5 times ULN) or TBIL (> 3 times ULN); For subjects with baseline AST, ALT, or TBIL > ULN, an increase in AST, ALT, or TBIL ≥ 50% (meeting the 3rd or 4th grade requirement) and a duration of ≥ 7 days. | Permanent discontinuation |
| Nephritis | Grade 2 or 3 elevation of serum creatinine | Suspend administration ^a^ |
|  | Grade 4 elevation of serum creatinine | Permanent discontinuation |
| Endocrine diseases | Symptomatic Grade 2 or 3 hypothyroidism  Grade 2 or 3 hyperthyroidism  Grade 2 or 3 pituitary inflammation  Grade 2 adrenal insufficiency  Grade 3 hyperglycemia or Type 1 diabetes | Suspend administration ^b^ |
|  | Grade 4 hypothyroidism  Grade 4 hyperthyroidism  Grade 4 pituitary inflammation  Grade 3 or 4 adrenal insufficiency  Grade 4 hyperglycemia or type 1 diabetes | Permanent discontinuation |
| Dermatological adverse reactions | Grade 3 | Suspend administration ^a^ |
|  | Grade 4, Stevens-Johnson syndrome (SJS) or toxic epidermal necrolysis (TEN) | Permanent discontinuation |
| Thrombocytopenia | Grade 3 | Suspend administration ^a^ |
|  | Grade 4 | Permanent discontinuation |
| Other immune-related adverse reactions | Grade 3 or 4 elevated amylase or lipase  Grade 2 or 3 pancreatitis  Grade 2 myocarditis ^c^  Other immune-related adverse reactions of grade 2 or 3 occurring for the first time | Suspend administration ^a^ |
|  | Grade 4 pancreatitis or any grade of recurrent pancreatitis  Grade 3 or 4 myocarditis  Grade 3 or 4 encephalitis  Other immune-related adverse reactions of grade 4 occurring for the first time ^d^ | Permanent discontinuation |
| Recurrent or persistent adverse reactions | Recurrent grade 3 or 4 (excluding endocrine disorders)  Grade 2 or 3 adverse reactions within 12 weeks after the last administration  Not improved to grade 0-1 (excluding endocrine disorders)  Corticosteroids failed to reduce to ≤ 10 mg/day of prednisone equivalent within 12 weeks after the last administration. | Permanent discontinuation |

a: Resume administration after symptom improvement to grade 0-1 or baseline level.

*The safety of reinitiating Sintilimab treatment after myocarditis improves to grade 0-1 is still unclear.

b: Pituitary inflammation, adrenal cortical insufficiency, hypothyroidism/thyroid dysfunction, and type 1 diabetes can be re-administered when adequately controlled and requiring only physiological hormone replacement therapy.

c: The safety of resuming treatment with this product after myocarditis improves to grade 0-1 is still unclear.

d: For grade 4 laboratory abnormalities, the decision to discontinue medication should be based on accompanying clinical symptoms/signs and according to the clinical judgment of the investigator.

**Hepatic Arterial Infusion Chemotherapy**

In the chemotherapy regimen of HAIC, only the dose of oxaliplatin is adjusted. That is: when the tumor diameter > 10 cm and there is abundant blood supply, the dose of Oxaliplatin selected is 130 mg/m ^2^. For tumors ≤ 10 cm and with less abundant blood supply, the dose of Oxaliplatin selected is 85 mg/m ^2^. Additionally, when a large tumor significantly shrinks after several courses of HAIC treatment, a dose reduction may be appropriate, even down to 60 mg/m ^2^. Subsequent researchers can make appropriate dose adjustments based on the patient's actual treatment situation and tolerance, and ensure proper documentation.

***Note: If the AE can be determined to be an ADR related to one of the investigational drugs, and the investigator believes that the subject will benefit from continuing to use the other investigational drug in combination with HAIC treatment, the subject may use the other investigational drug in combination with HAIC treatment until the completion of the study treatment or the occurrence of other circumstances requiring early withdrawal/termination of the study.**

## Management of Investigational Drug

**5.4.1 Support for Investigational Drug**

Donafenib was approved for first-line treatment of advanced liver cancer in June 2021. Currently, among the recombinant humanized anti-PD-1 monoclonal antibodies marketed in China, Sintilimab has also been approved for liver cancer indications.

### 5.4.2 Packaging and Labeling of Investigational Drug

The drug is labeled with clinical trial information and includes the following details: manufacturer, protocol number, name of the investigational drug (indicating it is for clinical trial use), specifications, storage conditions, batch number, drug code, expiration date, usage instructions, etc. The label content will ensure compliance with regulatory requirements.

### 5.4.3 Receipt and Storage of Investigational Drug

The drugs will be provided by Innovent Biologics (Suzhou) Co., Ltd. and Suzhou Zelgen Biopharmaceuticals Co., Ltd. according to the enrollment plan anticipated by the research center. The investigational drugs will be transported to the research center by a third-party logistics company with transportation qualifications. Upon receipt of the investigational drugs, the designated receiving personnel at the research center will check the transportation condition, confirm the quantity and status of the drug bottles, complete the inventory and drug counting records, and finally fax the signed delivery note to Suzhou Zelgen or an authorized third party to confirm receipt of the drugs. The clinical trial medication can only be used for this study and must be stored according to the corresponding drug storage conditions, and it can only be managed by personnel authorized by the investigator. To fully control the distribution and use of the investigational drug, inventory registration must be conducted during each subject's administration visit.

### 5.4.4 Distribution and Retrieval of Investigational Drug

**Sintilimab injection:**

Subjects must receive intravenous infusion of Sintilimab injection in the presence of emergency medical facilities and personnel, and the personnel must have received training in emergency monitoring and management. The Sintilimab infusion solution will be prepared by the research center staff, and the corresponding empty bottles and packaging will be collected and destroyed by the research center staff according to the research center's SOP.

**Distribution and collection of donafenib tablets:**

On the first day of each cycle, the required donafenib tablets for that cycle will be distributed. If the patient purchases the medication, it will be dispensed by a qualified pharmacy according to the investigator's prescription. If it is a clinical trial medication, it will be dispensed by the research center's drug administrator according to the investigator's prescription.

All unused investigational drugs will be stored at the designated storage location of the clinical pharmacology institution at the research center according to the specified storage conditions. In the event of loss or damage to the drug, the drug administrator should document the occurrence in detail. At the end of the study, any remaining unused investigational drugs must be returned to Suzhou Zelgen Biopharmaceutical Co., Ltd.

### 5.4.5 Treatment/Medication Compliance

Subjects' compliance with treatment/medication and the research protocol includes their willingness to adhere to the various examinations, sampling, and other requirements specified in the research protocol. According to the investigator's decision, subjects may withdraw from the study due to non-compliance with visit schedules or investigational drug usage requirements.

**Donafenib tablets:**

The research nurse or authorized drug administrator must inquire about the quantity of drug taken/remaining unused drug quantity during the subject's next cycle visit to assess the subject's medication adherence.

Adherence = Actual dosage taken (tablets) / Planned dosage (tablets) × 100%.

The definition of planned dosage is as follows:

1. If the investigator has not recommended a dose reduction and/or discontinuation: Recommended dosage (tablets/day) × Days of medication.
2. The investigator recommends dose reduction: pre-reduction dosage (tablets/day) × pre-reduction days + post-reduction dosage (tablets/day) × post-reduction days;
3. The investigator recommends discontinuation of treatment with a duration not exceeding 2 weeks: pre-discontinuation dosage (tablets/day) × pre-discontinuation days + post-discontinuation dosage (tablets/day) × post-discontinuation days;
4. The investigator recommends discontinuation of treatment but the duration exceeds 2 weeks: compliance will not be calculated, and the subject must withdraw from this study;
5. Subjects who discontinue or reduce dosage on their own: handled as per 1).

Except for the above-mentioned item 4, if the subject's medication adherence is between 80% and 120%, it is considered good adherence. If adherence is below 80%, it is considered non-compliant. During the DLT observation period, if adherence is non-compliant, it will be regarded as the subject voluntarily withdrawing from the study. In other study phases, when a subject's adherence is deemed non-compliant, the research nurse or authorized medication management personnel must provide education on medication administration methods to improve the subject's adherence. If a subject experiences two or more instances of non-compliance consecutively, the investigator has the right to terminate that subject's medication treatment.

Research nurses or authorized drug administrators must closely monitor treatment compliance, and the dose and administration schedule of donafenib received by each subject must be recorded at each cycle. Reasons for delayed dosing, dose reduction, or missed doses must be documented in the CRF. Medication compliance should be assessed at each visit.

**Sintilimab injection:**

Subjects must receive intravenous infusion of sintilimab at the research center. The administration dose and timing of the investigational drug infused during each treatment cycle should be recorded in the CRF, and reasons for delayed administration, dose adjustments, or missed doses should also be documented in the CRF.

## Concomitant medication and concomitant treatment

Unless there is an absolute medical necessity, subjects should, in principle, refrain from using drugs or non-drug treatments not mentioned in the study protocol. If other medications must be used during the study due to treatment needs, this must be agreed upon by the investigator after assessment, and used under the investigator's guidance. It should be thoroughly documented in the original records and the concomitant treatment section of the CRF, including the reasons, methods, and start and end dates of the treatment.

Symptomatic treatments given due to adverse events, including transfusions of whole blood and blood products, antibiotic treatment, allergy treatment, and anti-diarrheal treatment, must be recorded, with detailed documentation of the treatment date, reasons, and medication dosage.

During the study period, subjects are allowed to receive comprehensive supportive care, such as antibiotics, nutritional support, correction of metabolic disorders, pain management, etc.

Subjects may use topical, ophthalmic, intra-articular, intranasal medications, as well as inhaled corticosteroids (with minimal systemic absorption). Short-term use of corticosteroids is permitted for prevention (e.g., contrast agent allergy) or treatment of non-autoimmune diseases (e.g., delayed allergic reactions caused by contact allergens), or to manage adverse events caused by the investigational drug.

Patients with inactive hepatitis B are allowed to take antiviral medications concurrently during the study.

Subjects are allowed to receive inactivated vaccines during the study period.

## Prohibited Medications and Treatments

During the treatment period with the investigational drug, the following medications or treatments are not allowed:

1. Other anti-tumor chemotherapy, radiotherapy, interventional/ablation therapy, targeted therapy, immunotherapy, hormone therapy (except for medications for allergic reactions), traditional Chinese medicine and traditional Chinese medicine preparations, any medications explicitly stated in the instructions to have anti-tumor effects or anti-tumor activity, surgery, bone marrow transplantation, stem cell rescue, or other clinical trial drugs; If the investigator determines that the subject requires any other specific anticancer therapy, the subject must discontinue the investigational drug treatment prior to receiving the new antitumor therapy;
2. Systemic immunosuppressive drugs, including but not limited to methotrexate, azathioprine, TNF-α inhibitors, IL-6R inhibitors, JAK inhibitors, doses exceeding 10 mg/day of prednisone or other systemic corticosteroids with equivalent effects, and traditional Chinese medicine and formulations that may have immunomodulatory effects;
3. Warfarin and antiplatelet therapy;
4. Any other clinical investigational treatment;
5. Live attenuated vaccine.

In addition, during the study treatment, concomitant use of CYP3A4 inducers (such as phenytoin, carbamazepine, rifampicin, rifabutin, phenobarbital, etc.) should be avoided as much as possible; Caution should be exercised when concomitantly using CYP2B6 and CYP2C8 substrates (CYP2B6 substrates: amifampridine, cyclophosphamide, ifosfamide, chlorpromazine, methadone, etc.; CYP2C8 substrates: Paclitaxel, Tosemide, Amodiaquine, Rosuvastatin, Repaglinide, etc.).

## Drug Overdose

Drug overdose is defined as the subject receiving more than 20% of the planned dose for any reason.

Adverse events (AEs) caused by drug overdose need to be classified as serious adverse events (SAEs) and reported and managed symptomatically according to Section 8.7.2.

# Research Process

The research flowchart is detailed in Appendix 1.

## Screening Period (-14 days to 0 days)

1. The examinations in the screening items of this phase are part of the routine clinical treatment process. The results of examinations conducted at this research center prior to the patient's signing of the informed consent form (within 14 days before screening assessment) can be used to evaluate the eligibility of subjects for inclusion and serve as the baseline for enrolled subjects;
2. Demographic data: including date of birth, gender, race/ethnicity;
3. History of tumor and treatment: date of tumor diagnosis, start/end dates of previous treatment regimens, best treatment assessment, date of disease progression; history of radiotherapy must include start/end dates and the site of radiotherapy; Any significant previous procedures (such as gastroscopy, puncture biopsy, and other diagnostic or therapeutic invasive procedures) must be recorded in the CRF, including start and end dates, name of the procedure, and site; Date of liver cancer radical surgery, maximum diameter of the tumor before and during surgery, number of nodules, presence of PVTT and its classification (for PVTT classification, see [Appendix Six](#附录九) ), as well as postoperative pathological results, including pathological diagnosis, MVI, satellite lesions, etc.;
4. Medical history/treatment history: Collect all past medical history and treatment history, excluding this indication, that began before signing the ICF and is considered relevant to this study;
5. Alcohol history, smoking history;
6. ECOG PS score: It is recommended that the ECOG PS evaluation be conducted by the same investigator throughout the study period, see [Appendix Five](#附录七) ;
7. Child-Pugh score and BCLC staging (Child-Pugh score see [Appendix Four](#附录六) );
8. Safety data:

- Physical examination: including head, eyes, ears, nose, throat, neck, heart, chest (including lungs), abdomen, limbs, skin, lymph nodes, nervous system, and general condition of the subjects, as well as height and weight measurements; subjects should wear indoor clothing and remove shoes when measuring weight;
- Vital signs examination: temperature, respiration, blood pressure, and heart rate; Blood pressure and heart rate should be measured after the patient rests in a sitting position for 5 minutes;
- Complete blood count: red blood cell count, hemoglobin, hematocrit, white blood cell count and differential (neutrophils, lymphocytes, eosinophils, monocytes, basophils), and platelet count;
- Urinalysis: specific gravity, pH, urine glucose, protein, casts, ketones, urine red blood cells, urine white blood cells; If there are two consecutive urine protein tests showing ++ or higher, or if the doctor determines the results to be abnormal and clinically significant, a 24-hour urine protein quantification test is required;
- Routine stool examination: fecal occult blood;
- Blood biochemistry examination: total protein, albumin, globulin, blood glucose, urea/urea nitrogen, creatinine, alkaline phosphatase, lactate dehydrogenase, total bilirubin, direct bilirubin, indirect bilirubin, AST, ALT, γ-glutamyl transferase, calcium, phosphorus, magnesium, potassium, sodium, chloride, amylase, uric acid, creatine kinase, troponin;
- Coagulation function tests: prothrombin time (PT), activated partial thromboplastin time (APTT), thrombin time (TT), international normalized ratio (INR);
- Thyroid function tests: Thyroid-stimulating hormone (TSH), serum free triiodothyronine (FT3), serum free thyroxine (FT4);
- Virology tests: Hepatitis B surface antibody (HBsAb), Hepatitis B surface antigen (HBsAg), Hepatitis B e antigen (HBeAg), Hepatitis B e antibody (HBeAb), and Hepatitis B core antibody (HBcAb), HBV DNA, HCV antibody or RNA, HIV antibody; If the subject has hepatitis B test results within 2 weeks prior to the date of signing the informed consent form, the subject does not need to repeat the examination. If the subject has HCV antibody or RNA, or HIV antibody test results conducted at this research center within 3 months prior to the date of signing the informed consent form, the subject does not need to repeat the examination.
- Pregnancy test (for female patients of childbearing potential, conducted on days -3 to 0): If the urine pregnancy test result cannot be confirmed as negative, a serum pregnancy test must be conducted, and the serum pregnancy result shall prevail.
- 12-lead electrocardiogram examination;
- Cardiac color Doppler ultrasound examination, with particular attention to the evaluation of left ventricular ejection function;

1. AFP testing;
2. CT/MRI examinations, with sites including the chest and abdomen; if clinical indications or suspicion of metastasis arise in other areas, CT/MRI examinations will be conducted; Chest imaging examinations should primarily use CT scans, while abdominal imaging examinations should primarily use enhanced MRI; if the subject is allergic to CT contrast agents, has poor renal function, or has other reasons that make CT examinations unsuitable, MRI will be used instead of CT; For CT/MRI examinations conducted as part of routine diagnosis prior to the subject signing the ICF, if they were performed within 4 weeks before enrollment and at this research center, they do not need to be repeated;
3. Review inclusion and exclusion criteria, and assess the eligibility of subjects for inclusion;
4. Record adverse events: Adverse events occurring from the signing of the informed consent form (ICF) until the first administration of the treatment should be recorded on the medical history page of the case report form (CRF);
5. Record concomitant medication/treatment: All medications/treatments received by subjects must be recorded in the CRF starting from 28 days prior to enrollment, including the generic name of the drug, dosage, frequency, route of administration, the reason for using the drug/treatment, and the start and end dates. Any new changes in drug treatment must be continuously updated.

Patients may retest laboratory parameters that do not meet the inclusion criteria within the screening window. Laboratory assessments are conducted as part of the screening evaluation and do not need to be repeated prior to medication administration.

## Donafenib monotherapy phase (Day 1 to Day 3~7)

After subjects are screened and qualified, they will first enter the donafenib monotherapy phase and will begin taking donafenib within 24 hours of enrollment, with the day of taking donafenib recorded as D1. The administration dose is 200 mg bid daily, continuing for 3~7 days.

All data obtained from these evaluations must be supported by the patient's original records. The CRF does not serve as original records.

1. Safety checks must be completed before each medication administration, and blood collection must not occur earlier than 3 days prior to medication administration. Results must be determined by the investigator to meet the criteria for continuing medication before treatment can begin. The items collected are the same as those in the screening phase, except for the following items:

- A limited physical examination based on symptoms should be conducted; height measurement is not necessary.
- If blood routine, blood biochemistry, urine routine, stool routine, coagulation function, electrocardiogram examination, and vital signs are performed within 3 days prior to the first administration, there is no need to repeat the corresponding examinations on Day 1 of the donafenib monotherapy period.
- Virology testing does not need to be repeated on Day 1 of the donafenib monotherapy period; thereafter, HBV DNA should only be tested in known HBV-positive patients and HCV RNA in known HCV-positive patients.
- Thyroid function does not need to be rechecked on Day 1 of the donafenib monotherapy period; if there are clinically significant changes in thyroid function during treatment, it is recommended to consult endocrinology and rule out pituitary dysfunction.
- Cardiac color Doppler ultrasound does not need to be repeated on day 1 of the donafenib monotherapy period; thereafter, it should only be performed if the subject has clinical indications (such as ECG abnormalities, chest tightness, cyanosis, dyspnea, etc.) or if the investigator deems it necessary.
- Pregnancy tests do not need to be repeated on day 1 of the donafenib monotherapy period; thereafter, a urine pregnancy test can be conducted, and if the result is positive, a blood pregnancy test should be performed.

1. AFP testing: does not need to be repeated on day 1 of the donafenib monotherapy period.
2. ECOG PS score collection. If it is conducted within 3 days prior to the first administration, there is no need to repeat the collection on day 1 of the monotherapy period with donafenib ;
3. Investigational drug treatment (donafenib monotherapy);
4. Dispense the treatment drug for the donafenib monotherapy period;
5. Conduct DLT observation after administration: applicable only to patients entering the safety induction phase, i.e., the first 6 patients enrolled;
6. Collect concomitant medication/treatment;
7. Collect adverse events: the adverse events of subjects must be closely monitored throughout the entire research process.
   1. Combination treatment period (the first use of Sintilimab is designated as CIDI, with visits every 3 weeks, time window ±3 days)

After 3-7 days of monotherapy with donafenib , if the subjects are well-tolerated to donafenib during the monotherapy period as assessed by the investigator, subsequent subjects may receive intravenous infusion treatment with Sintilimab injection, and begin approximately 1 day of HAIC treatment on the same day or the day after the infusion of Sintilimab.

All visits during the combination treatment period will be scheduled according to the appropriate calendar days starting from the combination treatment period C1D1 (the day of first use of Sintilimab), with a treatment cycle of every 3 weeks (the length of the 3-week cycle is fixed, regardless of whether the investigational drug is paused). An allowable visit time window of ±3 days is permitted. If at any time during the study the treatment with the investigational drug is paused, all study visits, safety, and efficacy assessments should continue according to the evaluation schedule based on the appropriate calendar days from the combination treatment period C1D1. All data obtained from these assessments must be supported by the original records of the patient. The CRF does not serve as the original record.

1. CT/MRI Examination: CT/MRI examination once every 6 weeks (relative to the combination treatment period C1D1, time window ±3 days). Chest and abdomen must be examined; other sites should only undergo CT/MRI examination if there are clinical indications of metastasis. During the study period, the same imaging techniques (scanning equipment and methods as well as imaging parameters) used at baseline must be employed, and the measurement methods must remain consistent with baseline, ideally assessed by the same investigator. If the investigator suspects that the subject may have progression, an unscheduled CT/MRI examination may be conducted; if the unscheduled examination does not reveal progression, subsequent evaluations should be conducted as close as possible to the originally scheduled examination time. If the subject confirms disease progression, subsequent CT/MRI examinations will no longer be required.
2. Safety data: **CIDI does not require repeated safety checks, only when the subject has corresponding clinical indications or the investigator deems it necessary.** Safety checks from C2D1 onwards must be completed before each administration of Sintilimab, and the blood collection time cannot be earlier than 3 days before administration. The results must be determined by the investigator to meet the criteria for continued medication before treatment can begin. The collection items are the same as during the screening period, except for the following items:

- A limited physical examination based on symptoms should be conducted; height measurement is not necessary.
- Virological testing: HBV DNA testing is only performed on known HBV-positive patients, and HCV RNA testing is only performed on known HCV-positive patients;
- Thyroid function tests: If there are clinically significant changes in thyroid function during treatment, it is recommended to consult the endocrinology department and rule out pituitary dysfunction;
- Cardiac ultrasound: Only performed if the subject has clinical indications (such as abnormal ECG, chest tightness, cyanosis, dyspnea, etc.) or if deemed necessary by the investigator;

1. AFP testing: **No need for repeat testing during combination therapy period C1D1;**
2. ECOG PS score collection: **No need for repeat testing during combination therapy period C1D1;**
3. Investigational drug treatment: donafenib + Sintilimab;
4. HAIC treatment: Infusion of sintilimab starts on the same day or the second day
5. Child-Pugh assessment: Once every 3 weeks, with a time window of ±3 days relative to the start of combination therapy on C1D1 ;
6. Evaluate medication adherence from the previous cycle and supplement the necessary donafenib treatment drugs for the next treatment cycle;
7. Conduct DLT observation after administration: applicable only to patients entering the safety induction phase, i.e., the first 6 patients enrolled; The observation and assessment period is: from the first use of donafenib to 21±3 days after the first HAIC treatment;
8. Collect concomitant medication/treatment;
9. Collect adverse events: the adverse events of subjects must be closely monitored throughout the entire research process.

## End of treatment visit (confirmation of end/discontinuation of treatment within 7 days)

1. Safety data: Collection items are the same as during the treatment period; if the urine pregnancy test result cannot be confirmed as negative, a serum pregnancy test must be conducted, with the serum pregnancy result being definitive;
2. AFP testing;
3. CT/MRI examination;
4. ECOG PS score collection.
5. Evaluate medication adherence from the previous cycle;
6. Collect concomitant medication/treatment;
7. Collect adverse events.

If a patient stops treatment during a study visit, there is no need to repeat the treatment termination visit assessment.

## Follow-up period (every 12 weeks, with a window of ±7 days)

### Safety follow-up

Subjects who complete treatment with the investigational drug will enter the follow-up period, recording all adverse events and concomitant medications for 30 days (±7 days) after the last administration or before receiving new antitumor therapy (whichever occurs first), as well as all new antitumor therapies after the last administration. For all adverse events or subjects who discontinue study treatment due to laboratory abnormalities, follow-up must be conducted until the event resolves or returns to baseline or better, the investigator confirms the event is stable, the subject withdraws consent, or the subject is lost to follow-up, and the results must be recorded in the CRF. Every effort should be made to follow up on all serious adverse events deemed related to the investigational drug or study-related procedures until a final outcome can be reported.

### Efficacy Follow-up

Subjects who do not experience disease progression or initiate new antitumor therapy upon discontinuation of the investigational drug will continue to undergo CT/MRI scans, ECOG PS assessments, and FACT-Hep scale collections every 12 weeks (within 2 years of the first administration) or every 24 weeks (after 2 years of the first administration) until disease progression occurs, new antitumor therapy is initiated, the subject withdraws consent, is lost to follow-up, dies, or the study is terminated early (whichever occurs first). During this period, if the investigator suspects that the subject has experienced progression, an unscheduled CT/MRI examination may be conducted. Once the subject stops efficacy follow-up, the reason for termination should be recorded in the CRF form. For patients who become pregnant, all tumor assessments must be halted, regardless of whether progression has occurred.

### Survival follow-up

Survival follow-up is required after the investigator determines there is evidence of disease progression or the initiation of new antitumor therapy. Survival follow-up is conducted every 12 weeks, with telephone follow-ups to the subject or their relatives to record the subject's survival status, until the subject withdraws the ICF, is lost to follow-up, dies, or the study is terminated early (whichever occurs first).

# Evaluation indicators

## Primary efficacy evaluation indicators

- Objective response rate (ORR)

(Tumor assessment using both RECIST v1.1 and mRECIST criteria, with RECIST v1.1 as the primary criterion)

## Secondary efficacy evaluation indicators

- Based on overall survival (OS)
- Time to disease progression (TTP)
- Progression-free survival (PFS)
- Disease control rate (DCR)
- Duration of response (DOR)
- ECOG PS score temporal changes
- AFP temporal changes

(Tumor assessment using both RECIST v1.1 and mRECIST criteria, with RECIST v1.1 as the primary criterion)

## Safety evaluation indicators

- Analysis of adverse events and serious adverse events

## Definition of efficacy indicators

### Progression-free survival (PFS)

Progression-free survival time refers to the duration from randomization to tumor progression or death from any cause (with tumor progression assessed using both RECIST v1.1 and mRECIST criteria, primarily based on RECIST v1.1). If a subject is lost to follow-up during the study and no imaging evidence of progression or death is obtained, it will be recorded as censored, with the censoring time being the last follow-up date confirming no progression. For subjects whose tumor progression has not been confirmed by the database cutoff date, censoring will be based on the date of the last imaging tumor assessment. The tumor progression date is defined as the date of the first imaging-confirmed tumor progression.

### Overall survival （overall survival, OS ）

The time from the date of enrollment to the date of death from any cause. If the subject is still alive at the database cutoff date, censoring will be performed at the last survival follow-up date in the database.

### Time to progression (time to progress, TTP)

Time to progression refers to the time from randomization to the occurrence of objective tumor progression (tumor assessment will be conducted using both RECIST v1.1 and mRECIST criteria, with RECIST v1.1 as the primary standard). If imaging progression evidence is not obtained due to loss to follow-up during the follow-up period, it will be recorded as censored, with the censoring time being the last follow-up time confirming that there was no progression.

### Objective response rate (objective response rate, ORR)

The objective response rate is defined as the proportion of subjects with the best overall response confirmed as CR or PR (using both RECIST v1.1 and mRECIST criteria for tumor assessment, with RECIST v1.1 as the primary criterion). In calculating ORR, any partial or complete responses confirmed prior to any other antitumor therapy are counted as responses, regardless of how many assessments were missed prior to the response. When the best overall response of subjects is 'unknown' or 'not assessed', it is considered as non-response when estimating ORR.

### Disease control rate ( disease control rate, DCR)

The disease control rate is defined as the proportion of subjects achieving the best clinical efficacy of CR, PR, or SD (with tumor assessment using both RECIST v1.1 and mRECIST criteria, primarily based on RECIST v1.1).

- - 1. **Duration of response (DOR)**

Duration of response refers to the time from the first assessment of complete response (CR) or partial response (PR) to the first assessment of disease progression (PD) or death from any cause (with tumor assessment using both RECIST v1.1 and mRECIST criteria, primarily based on RECIST v1.1).

。

# Safety Monitoring and Reporting

## Adverse Events (AE)

According to the International Conference on Harmonisation (ICH) Technical Requirements for Registration of Pharmaceuticals for Human Use, 'Good Clinical Practice' (GCP), an AE refers to any unfavorable sign, symptom, or medical event that occurs after obtaining the patient's signed informed consent, regardless of whether there is a causal relationship with the treatment. Therefore, an AE may be any of the following:

1. An AE may be any unfavorable and unintended sign (including abnormal laboratory findings), symptom, or disease (new or exacerbated) that occurs after the use of a certain therapeutic drug and has a temporal relationship with the drug use;
2. Any new disease or exacerbation of pre-existing conditions (manifestations, frequency, or severity of existing conditions worsening);
3. Recurrence of intermittent disease conditions (such as headaches) that were not present during the baseline period;
4. Any deterioration in laboratory test results or other clinical examinations (e.g., ECG) that is accompanied by clinical symptoms, or that leads to changes in the study treatment or concomitant medication, or that results in the termination of the investigational drug treatment;
5. Adverse events (AEs) related to the interventions specified in the study protocol;
6. Signs, symptoms, or clinical sequelae suspected to be caused by an overdose of the investigational drug or concomitant medication (overdose itself is not reported as an AE/SAE).

**Events that do not meet the definition of an AE include:**

1. Medical or surgical procedures (e.g., endoscopy, appendectomy); the conditions leading to these procedures should be recorded as AEs;
2. Conditions of hospitalization that did not result in adverse medical events (due to social insurance and/or convenience hospitalization);
3. Planned hospitalization required by the protocol (e.g., for the use of the investigational drug);
4. Expected day-to-day fluctuations of pre-existing diseases or conditions present or detected at the start of the study, but without exacerbation;
5. Expected progression, signs, or symptoms of the disease/disorder being studied, unless the severity of the subject's condition exceeds expectations or progresses more rapidly.

Adverse signs, symptoms, or medical conditions that existed at the time of signing the informed consent form should be recorded as medical history.

## Serious Adverse Events (SAE)

Meets any of the following criteria:

1. Results in death (i.e., AE causes or leads to death);
2. Is life-threatening (i.e., the investigator believes that the AE places the patient at immediate risk of death);

Note: The term 'life-threatening' refers to the risk of death at the time of the event, and does not imply that a more severe event would lead to death.

1. Results in permanent or significant disability or functional impairment;

Note: The term 'disability' refers to a severe impairment of an individual's ability to perform normal life functions. This definition does not include clinically insignificant events, such as simple headaches, nausea, vomiting, diarrhea, influenza, and accidental injuries (e.g., ankle sprains), which may affect daily functioning but do not result in a significant loss of function.

1. Causing congenital malformations/birth defects;
2. Requires hospitalization or prolongation of current hospitalization ;

Note: Generally speaking, hospitalization refers to a patient being observed in a hospital or emergency department (typically at least overnight) and/or receiving treatment that is not suitable for a doctor's office or outpatient department.

Hospitalization for the following reasons is not reported as an AE, but the reason for hospitalization must be clearly documented in the medical record:

1. Planned hospitalization as specified in the study protocol (e.g., for administration of the investigational drug, or for efficacy assessment of this study);
2. Routine treatment or monitoring for the study indication;
3. Hospitalization solely due to related tumor progression;
4. Hospitalization due to pre-existing conditions, but must meet all of the following criteria:
   - - Hospitalization planned prior to the study, or hospitalization for elective surgery due to predictable disease progression during the study;
     - Subjects did not experience any AEs or disease progression.
5. Social reasons and treatment delays in the absence of deterioration in the patient's general condition.

The following hospitalization situations will not be considered SAEs but should be reported as AEs:

1. Subjects hospitalized due to the need for outpatient medical care outside of normal clinic hours.
2. Other important medical events that require medical and scientific judgment to determine whether expedited reporting is necessary, such as important medical events that may not immediately threaten life, result in death, or require hospitalization, but if medical measures are needed to prevent one of the above situations from occurring, are typically considered serious. For example, important treatments in the emergency room or allergic bronchospasm occurring at home, cachexia or seizures not requiring hospitalization, and the development of drug dependence or addiction, etc.
3. SAE as defined in the protocol:
4. Potential drug-induced liver injury, including elevated ALT or AST levels, accompanied by elevated bilirubin levels or clinical jaundice, as specifically defined in the Hais' criteria (see Section 8.8.7 for details).
5. AE caused by drug overdose, regardless of the CTCAE grading level.

If tumor progression (including fatal outcomes) is recorded using appropriate methods (such as according to the mRECIST criteria for solid tumors), it is not reported as a serious adverse event.

In cases where treatment is based on outpatient emergency visits that do not result in hospitalization and the events do not meet any of the SAE definitions provided above, they are not reported as serious adverse events.

The terms 'serious' and 'severe' are not synonymous; 'severity' and 'seriousness' are also not synonymous. Severity refers to the intensity of the AE (as determined by CTCAE v5.0; see [Section 8.6](#_不良事件严重程度评价) ); The medical significance of the event itself may be relatively minor (for example, severe headache with no other findings).

## Special adverse event management for donafenib tablets

### Hand-foot syndrome ( HFSR)

**Definition of HFSR:** It refers to the numbness or erythema of the palms and soles, which is a type of skin toxicity that is more pronounced in areas under pressure or stress. It can occur in cancer patients undergoing chemotherapy or molecular targeted therapy. The characteristics of HFSR include numbness, sensory loss, abnormal sensations, tingling, absence of pain or pain, skin swelling, erythema, desquamation, fissures, indurated blisters, and severe pain, among others.

**HFSR grading:**

- Grade 1: Numbness/tingling/abnormal sensations in the hands and/or feet, painless swelling or erythema, and/or discomfort that does not affect normal activities;
- Grade 2: Painful erythema and swelling in the hands and/or feet and/or discomfort that affects the patient's daily activities;
- Grade 3: Moist desquamation, ulcers, blisters, or severe pain in the hands and/or feet and/or severe discomfort that prevents the patient from working or performing daily activities. Pain is intense, with loss of skin function, and is relatively rare;

**Symptomatic treatment and management of HFSR:** Implement necessary supportive care, including: enhancing skin care, maintaining skin cleanliness, avoiding secondary infections; avoiding pressure or friction; Use of emollients or lubricants, vitamin ointments, and topically applied lotions or lubricants containing urea and corticosteroids; antifungal or antibiotic treatment topically as needed.

### Special management of hypertension related to targeted therapy

**Situations of hypertension caused by targeted drug therapy:** Vascular endothelial growth factor (VEGF) signaling pathway inhibitors such as Bevacizumab and Sorafenib can cause hypertension or exacerbate pre-existing hypertension. Its main mechanisms of action may include: decreased secretion of NO/PGI2 from endothelial cells/platelets, abnormal vascular density (small vessels and capillaries), vascular stiffness, and dysfunction of endothelin. Sunitinib may also lead to a decrease in left ventricular ejection fraction (LVEF).

**Monitoring and management of this type of hypertension:** Blood pressure should be monitored weekly for 6 weeks prior to the initiation of targeted drug therapy. Once hypertension occurs, the following standard treatment medications may be administered: angiotensin II receptor (AT1) blockers, β-blockers, diuretics, angiotensin-converting enzyme inhibitors (ACEI), or a combination of the aforementioned medications.

**Reference for the treatment of hypertension caused by similar drugs:** Hypertension after sorafenib treatment generally occurs 1 to 2 weeks after medication initiation, and can often be managed with routine antihypertensive treatment. For hypertension that is difficult to control, it can generally be alleviated by reducing the dosage of the targeted drug or discontinuing it.

**Optimized choice of drugs for hypertension caused by targeted therapy (non-hepatic metabolism):**

- Valsartan (Diovan), 80~320 mg once daily (Qd);
- Atenolol (Tenormin), 50~100 mg Qd;
- HCTZ (Hydrochlorothiazide), 12.5~100 mg Qd;
- Telmisartan (Micardis), 20~80 mg Qd;
- For patients with difficult-to-control blood pressure: Amlodipine (Norvasc), 2.5~10 mg Qd.

**Clinical staging of hypertension and conventional management methods for each stage**

- **Prehypertension:** (120~139/80~89 mmHg, or systolic blood pressure of 120~139 mmHg) no indication for antihypertensive medication, only monitor blood pressure.
- **Stage 1 hypertension:** (140~159/90~99 mmHg, or systolic blood pressure of 140~159 mmHg) initiate antihypertensive medication while monitoring blood pressure; Most commonly, thiazide diuretics are used; consideration may also be given to ACE inhibitors, angiotensin receptor blockers, β-blockers, and calcium channel blockers. If blood pressure control is inadequate, the combination of two antihypertensive agents may be considered.
- **Stage 2 hypertension:** (160~179/100~109 mmHg, or systolic blood pressure ≥ 160~179 mmHg) requires the combination of two medications (usually a thiazide diuretic with either an ACE inhibitor or a β-blocker or a calcium channel blocker); monitor blood pressure.
- **Stage 3 hypertension:** (≥ 180/110 mmHg, or systolic blood pressure ≥ 180 mmHg) requires the combination of two medications (usually a thiazide diuretic with either an ACE inhibitor or a β-blocker or a calcium channel blocker); Closely monitor blood pressure, assess other risk factors (such as target organ damage, diabetes, and accompanying clinical symptoms), and take appropriate measures.
- **Hypertensive crisis:** Refers to a severe clinical condition where blood pressure is excessively elevated, with diastolic pressure exceeding 16.0~17.3 kPa (120~130 mmHg). There is currently no unified classification method domestically and internationally. Recently, from a clinical treatment perspective, it has been divided into two types:
- Hypertensive emergency with diastolic pressure > 16.0 kPa (120 mmHg), accompanied by acute or progressive target organ damage, such as cerebral infarction, intracranial or subarachnoid hemorrhage, hypertensive encephalopathy, etc., among which progressive or acute hypertension based on chronic primary hypertension is the most common (approximately 40%~50%).
- Hypertensive emergency, diastolic pressure > 16.0 kPa (120 mmHg) without or only mild organ damage. Use sodium nitroprusside or nifedipine for rapid blood pressure reduction, diazepam and phenobarbital to stop seizures, furosemide and mannitol for dehydration, sodium excretion, and lowering intracranial pressure.

**Principles of using conventional antihypertensive drugs:** Currently, the common combinations for hypertension treatment involve ACE inhibitors (or AT1 antagonists) with diuretics; calcium channel blockers with β-blockers; ACE inhibitors with calcium channel blockers; diuretics with β-blockers; α-blockers with β-blockers. The following is an overview of the commonly used antihypertensive medications in clinical practice.

- **Diuretics:** Diuretics are primarily used for mild to moderate hypertension, especially in elderly patients with hypertension or concurrent heart failure. They are contraindicated in patients with gout and should be used with caution in patients with diabetes and hyperlipidemia. Low doses can avoid adverse reactions such as hypokalemia, impaired glucose tolerance, and arrhythmias. Hydrochlorothiazide 12.5 mg can be chosen, 1-2 times daily; indapamide 1.25-2.5 mg, once daily. Furosemide is only used in cases of concurrent renal failure.
- **Beta-blockers:** Beta-blockers are primarily used for mild to moderate hypertension, especially in young and middle-aged patients with a resting heart rate greater than 80 beats per minute or those with concomitant angina. Contraindicated in patients with cardiac conduction block, asthma, chronic obstructive pulmonary disease, and peripheral vascular disease. Use with caution in insulin-dependent diabetes mellitus patients. Options include Metoprolol 50 mg, 1-2 times daily; Atenolol 25 mg, 1-2 times daily; Bisoprolol 2.5-5 mg, once daily; and Betaloc 5-10 mg, once daily. Beta-blockers can be used for heart failure, but their usage differs completely from that for lowering blood pressure, and caution should be exercised.
- **Calcium channel blockers:** Calcium channel blockers can be used for various degrees of hypertension, especially in elderly patients with hypertension or stable angina. Non-dihydropyridine calcium channel blockers are contraindicated in patients with cardiac conduction block and heart failure. Short-acting dihydropyridine calcium channel blockers are contraindicated in unstable angina and acute myocardial infarction. Long-acting formulations are preferred, such as felodipine sustained-release tablets 5~10 mg, once daily; nifedipine controlled-release tablets 30 mg, once daily; amlodipine 5~10 mg, once daily; lercanidipine 4~6 mg, once daily; verapamil sustained-release tablets 120~240 mg, once daily. Under normal circumstances, nifedipine or nicardipine ordinary tablets 10 mg may also be used, 2 to 3 times daily. Use nifedipine rapid-release capsules with caution.
- **Angiotensin-converting enzyme inhibitors:** Angiotensin-converting enzyme inhibitors are mainly used for patients with hypertension combined with diabetes, or those with heart failure or renal impairment with proteinuria. Contraindicated in patients with pregnancy, renal artery stenosis, or renal failure (serum creatinine > 265 μmol/L or 3 mg/dL). The following formulations may be chosen: Captopril 12.5~25 mg, 2~3 times daily; Enalapril 10~20 mg, 1~2 times daily; Perindopril 4~8 mg, once daily; Silapril 2.5~5 mg, once daily; Benazepril 10~20 mg, once daily; Ramipril 2.5~5 mg, once daily; Lisinopril 20~40 mg, once daily.
- **Angiotensin II receptor antagonists:** AT1 antagonists, such as Losartan 50~100 mg, once daily, Valsartan 80~160 mg, once daily. The applicable and contraindicated populations are the same as for ACEI, currently mainly used for patients who develop a dry cough after ACEI treatment.

### Gastrointestinal bleeding

If gastrointestinal bleeding occurs, with stool occult blood (++ or higher), hematemesis, or fresh blood in stool, close observation should be strengthened. For those diagnosed with upper gastrointestinal bleeding, fasting should be enforced, and acid suppression, gastric mucosal protection, and hemostasis (such as tranexamic acid, Lysteda, etc.) should be administered; octreotide may be used if necessary. For those with lower gastrointestinal bleeding, active hemostatic measures and supportive symptomatic treatment should be provided; if bleeding cannot be controlled, surgical intervention may be necessary.

### Proteinuria

If there are two consecutive instances of urine protein (++), a 24-hour urine protein quantification should be performed.

- Grade 1: Urinary protein 1+, or < 0.3 g%, or < 3 g/L.
- Grade 2: Urinary protein 2~3+, or 0.3~1.0 g%, or 3~10 g/L.
- Grade 3: Urinary protein 4+, or > 1.0 g%, or > 10 g/L.
- Grade 4: Nephrotic syndrome.

### Diarrhea

When subjects experience diarrhea of grade 2 or higher, appropriate measures such as antidiarrheal treatment and rehydration should be taken.

- Grade 1: Daily bowel movements exceed pre-treatment < 4 times;
- Grade 2: Daily bowel movements exceed pre-treatment 4~6 times, intravenous rehydration < 24 hours;
- Grade 3: Increase of ≥ 7 times/day or incontinence; intravenous treatment ≥ 24 hours; hospitalization;
- Grade 4: Life-threatening secondary symptoms.

### Elevated bilirubin

If grade 2 or higher elevation of bilirubin occurs, the physician may determine appropriate management measures based on the patient's condition.

- Grade 1: > 1.5 × ULN;
- Grade 2: > 1.5~3.0 × ULN;
- Grade 3: 3.0~10.0 × ULN;
- Grade 4: > 10.0 × ULN.

### Elevated transaminases

If grade 2 or higher elevation of transaminases occurs, the investigator may determine appropriate management measures such as liver treatment based on the subject's condition.

- Grade 1: > 3.0 × ULN;
- Grade 2: > 3.0~5.0 × ULN;
- Grade 3: > 5.0~20.0 × ULN;
- Grade 4: > 20 × ULN.

## Management of special adverse events for recombinant humanized anti-PD-1 monoclonal antibody

### Infusion-related reactions

Clinical symptoms of infusion-related reactions include fever, chills, nausea, itching, vascular edema, hypotension, headache, bronchospasm, urticaria, rash, vomiting, muscle pain, drowsiness, or hypertension. Severe reactions may include acute respiratory distress syndrome (ARDS), myocardial infarction, ventricular fibrillation, and cardiogenic shock. Therefore, subjects in this study must be closely monitored for related clinical symptoms. Guidelines for the management of infusion-related reactions are shown in Table 2, and the management of allergic reactions can be referred to in [Appendix Seven](#_附录).

In the event of a severe reaction, continuous electrocardiographic monitoring and the administration of rescue medications (including but not limited to adrenaline, corticosteroids, antihistamines, bronchodilators, and oxygen) are required for resuscitation. If infusion-related reactions or suspected events of CTCAE grade 2 or higher occur, infusion of recombinant humanized anti-PD-1 monoclonal antibody must be immediately stopped. During the first intravenous infusion of recombinant humanized anti-PD-1 monoclonal antibody, subjects' vital signs must also be monitored within 30 minutes after the end of the infusion. Subjects will be informed of the possible delayed symptoms following infusion and will be asked to contact their study physician if such symptoms occur.

If a severe allergic reaction occurs, subjects must be treated according to local best medical practices. If a subject experiences a CTCAE grade 2 or higher infusion-related reaction again, the subject must immediately discontinue the treatment permanently and withdraw from the study.

**Table 2: Treatment Adjustment Guidelines for Infusion-related Reactions**

| **CTCAE Grade** | **Adjustment Measures** |
| --- | --- |
| Grade 1: Mild | - Transient mild reaction; interruption of infusion and clinical intervention are not recommended. Reduce the infusion rate by 50% and closely monitor for any worsening symptoms. - Clinical intervention as necessary |
| Grade 2: Moderate | - Suspend Recombinant Humanized Anti-PD-1 Monoclonal Antibody Treatment, and immediately provide systemic treatment (e.g., antihistamines, NSAIDs, analgesics, intravenous fluids); When infusion-related reactions are relieved to grade 0-1, re-administer the treatment and reduce the infusion rate by 50%. - Closely monitor for any worsening symptoms during this period. - Take appropriate therapeutic intervention measures according to local medical practices. |
| Grade 3: Severe | - Immediately stop the infusion and remove the infusion line; the investigator will decide whether to re-administer based on the subject's actual condition. - If re-administering, during subsequent treatments, the infusion time should be at least 2 hours, and relevant prophylactic medications (such as diphenhydramine and NSAIDs) should be given, while closely monitoring for clinical symptoms related to infusion-related reactions. - Take appropriate therapeutic intervention measures according to local medical practices. |
| Grade 4: Life-threatening and requires urgent clinical intervention | - Subjects who experience grade 4 infusion-related reactions must immediately discontinue the medication permanently and withdraw from the study. - Take appropriate therapeutic intervention measures according to local medical practices. |

Abbreviation: NSAID = Non-Steroidal Anti-Inflammatory Drug.

### Severe allergic reactions

Antibody administration may cause allergic reactions. Therefore, it is crucial to provide appropriate medications and medical equipment to treat acute allergic reactions immediately, and researchers must be trained to recognize and manage allergic reactions. Research sites must be equipped with emergency rescue teams and equipment, as well as the capability to admit subjects to intensive care units if necessary. In the event of a severe allergic reaction, subjects must receive emergency treatment immediately according to local relevant diagnostic and therapeutic protocols. Subjects must be administered epinephrine and dexamethasone immediately, and continuous cardiac monitoring should be initiated; consideration may be given to obtaining serum IgE specimens. Subjects must permanently discontinue the study treatment and withdraw from the research. Subjects experiencing such symptoms must immediately inform the investigator.

**Allergic Reaction**

The National Institute of Allergy and Infectious Diseases (NIAID) and the Food Allergy and Anaphylaxis Network (FAAN) define an allergic reaction as a severe allergic reaction that occurs rapidly and may lead to death. These three types of allergic reactions cover 80% (Type 1) to 95% of cases (all three types).

1. Acute allergic reactions (ranging from minutes to hours) involve the skin, mucous membranes, or both (for example, widespread urticaria, itching or flushing, swollen lips or tongue), and at least one of the following conditions is present:
2. Difficulty breathing [for example, dyspnea, wheezing - bronchospasm, wheezing, decreased peak expiratory flow (PEF), hypoxemia];
3. Decreased blood pressure or symptoms of related end-organ dysfunction (for example, hypotensive shock, syncope, incontinence).
4. After exposure to allergens (for a few minutes to several hours), the patient may experience two or more of the following conditions:
5. Involvement of skin and mucosal tissues (e.g., extensive urticaria, itching, swelling of the lips and tongue);
6. Difficulty breathing (e.g., dyspnea, wheezing - bronchospasm, wheezing, decreased PEF, hypoxemia);
7. Decreased blood pressure or related symptoms (e.g., hypotensive shock, syncope, incontinence);
8. Persistent gastrointestinal symptoms (e.g., abdominal pain, vomiting).
9. Blood pressure decreases in patients after exposure to known allergens (within minutes to hours):
10. Infants and children: systolic blood pressure low (age-specific) or systolic blood pressure decreases below 30%;
11. Adults: systolic blood pressure below 90 mmHg or a reduction of more than 30% from baseline.

### Immune-related adverse events (irAE)

Recombinant humanized anti-PD-1 monoclonal antibody may be associated with the following significant irAEs: immune-related liver dysfunction, interstitial lung disease, pancreatitis, endocrine disorders [hypothyroidism (for replacement therapy of hypothyroidism, see [Appendix 8](#附录十) ), hyperthyroidism], and immune-related hyperglycemia.

In addition, irAEs also include the following clinically significant potential events: exfoliative dermatitis, pigmentary retinopathy, arthritis, myocarditis, hemolytic anemia, partial seizures that may occur in subjects with inflammation in the cerebral hemispheres, adrenal insufficiency, myasthenia gravis, optic neuritis, and rhabdomyolysis.

For suspected irAEs, close monitoring of the relevant system functions is required, and a thorough evaluation should be conducted to determine the cause and exclude other reasons. Overall, based on the severity of the events, it may be necessary to suspend or permanently discontinue the recombinant humanized anti-PD-1 monoclonal antibody and/or provide symptomatic treatment, such as corticosteroids. When administering 1-2 mg/kg of prednisone or a glucocorticoid with equivalent efficacy, a gradual dose reduction should begin after the event has been alleviated to grade 0-1. If the irAE remains at grade 0-1 at this time, re-administration of recombinant humanized anti-PD-1 monoclonal antibody therapy should be considered. In the event of an irAE of grade 3 or higher (excluding endocrine system diseases), immediate permanent discontinuation of the drug and withdrawal from the study is required.

## Assessment of the causal relationship of adverse events

The investigator analyzes the correlation between adverse events and the investigational drug based on the established criteria, categorizing them into five classes: 'definitely related, probably related, possibly unrelated, definitely unrelated, and unable to determine.' Adverse events classified as 'definitely related, probably related, and unable to determine' are counted as related to the investigational drug.

### Criteria for determining the relationship between adverse events and investigational drug

- Is there a reasonable temporal relationship?
- Does it conform to known types of adverse reactions?
- Do adverse events disappear or lessen after discontinuation or dose reduction?
- Do adverse events recur after re-administration?
- Is there a possibility of other causes for the adverse events?

### Criteria for determining the relationship between adverse events and investigational drug

1. Definitely related:

- Reasonable temporal relationship+
- Conforms to known types of adverse reactions+
- Adverse events disappear or lessen after discontinuation or dose reduction+
- Adverse events recur after re-administration+
- There are other reasonable explanations -

1. Possibly related:

- Reasonable temporal relationship+
- Does it conform to known types of adverse reactions ±?
- Do adverse events disappear or lessen after discontinuation or dose reduction ±?
- Do adverse events reappear after re-administration of the drug?
- Is there a possibility that other reasons caused the adverse events ±?

1. Possibly unrelated:

- Reasonable time relationship -
- Does it conform to known types of adverse reactions -
- Do adverse events disappear or lessen after discontinuation or dose reduction ±?
- Do adverse events reappear after re-administration of the drug?
- Is there a possibility that other reasons caused the adverse events ±?

1. Definitely unrelated:

- Reasonable time relationship -
- Does it conform to known types of adverse reactions -
- Do adverse events disappear or lessen after discontinuation or dose reduction -
- Do adverse events reappear after re-administration of the drug -
- Is there a possibility that other reasons caused the adverse events +

1. Unable to determine: necessary evaluation data is unavailable

## Assessment of the severity of adverse events

The severity of adverse events (AEs) will be evaluated according to CTCAE v5.0 (see [Appendix 1](#附录一) ). If an AE occurs that is not within the range specified by this standard, the severity will be assessed using the following Table 3:

**Table 3 Adverse Event Severity Rating Scale for Events Not Specifically Listed in CTCAE**

| Level | Severity |
| --- | --- |
| 1 | Mild; asymptomatic or mild symptoms; clinical or diagnostic observation only; or no intervention required |
| 2 | Moderate; requires minimal, local, or non-invasive intervention; or age-appropriate instrumental activities of daily living are limited ^a^ |
| 3 | Severe or medically significant but not immediately life-threatening; requires hospitalization or prolongation of existing hospitalization; disability; or limitations in self-care activities of daily living ^b, c^ |
| 4 | Life-threatening consequences, or requiring emergency treatment ^d^ |
| 5 | Deaths related to adverse events ^d^ |

CTCAE Common Terminology Criteria for Adverse Events.=

a. Instrumental activities of daily living refer to activities such as cooking, shopping for groceries or clothes, making phone calls, and managing finances;

b. Examples of activities of daily living include bathing, dressing, eating, using the toilet, and taking medication, which are activities that non-bedridden patients can engage in;

c. If an event is classified as a 'serious medical event,' it must be reported as an SAE as described in section 8.7.2;

d. Grade 4 and 5 events must be reported as SAEs (please refer to Section [8.7.2](#_严重不良事件的报告) for reporting guidelines).

## Recording and reporting of adverse events and serious adverse events

### Recording and reporting of adverse events

During the study period, all AEs will be recorded until 60 days after the last administration of the investigational drug or the start of a new antitumor therapy, whichever occurs first. Adverse events should be described using a single diagnosis (rather than specific underlying symptoms and signs) whenever possible, including laboratory abnormalities that constitute AEs. When a definitive diagnosis cannot be made, each sign or symptom should be recorded as an independent adverse event.

After signing the informed consent form, adverse events should be investigated through non-leading questions directed at the subjects during the screening process and at each visit during the study. Methods for identifying adverse events also include active reporting by patients between the screening process or visits, or obtaining information through physical examinations, laboratory tests, or other assessments. Each adverse event should be evaluated as thoroughly as possible to determine:

- Severity grading (CTCAE v5.0) (see Section 8.6)
- Duration (start and end dates)
- Relationship to study treatment (the reasonable possibility that the AE is related to the study treatment) (see Section 8.5)
- Measures taken regarding the study or study treatment (none, dose adjustment, temporary interruption of treatment, permanent discontinuation of treatment, unknown, not applicable)
- Whether medication or treatment was administered (no concomitant medication/non-drug treatment, concomitant medication/non-drug treatment)
- Outcomes (not recovered/not relieved, recovered/relieved, in recovery/in relief, recovery/relief with sequelae, death, unknown)
- Whether it is a serious adverse event

In accordance with Chinese GCP regulations, all AEs occurring during the clinical research process, regardless of whether they are suspected to be related to the investigational drug, should be addressed with the following measures:

1. The investigator should immediately take appropriate protective measures for the subjects to ensure their safety; If concomitant medications or non-drug treatments are administered, this measure should be recorded in the adverse event CRF;
2. If the study is terminated, the investigator should also regularly check the subjects and fill in the termination date (the date of stopping the administration of the investigational drug), the reason for termination, and the detailed process in the CRF;
3. The investigator should follow up on all occurring AEs until any of the following situations occur:

- AE resolves or improves to baseline level or better;
- The investigator confirms that the event is stable and is not expected to improve further;
- Subject death;
- The subject has lost contact or withdrawn consent;
- The investigator confirms that the AE is unrelated to the study treatment;
- The subject has started a new antitumor therapy.

Any AE should be documented in detail on the CRF, and any changes in severity, suspected relationship to the study treatment, required therapeutic interventions, and outcomes should be assessed at each visit.

### Reporting of serious adverse events

Collect information on all SAEs and record it on the serious adverse event report form; To provide a comprehensive clinical report, all applicable sections of the form must be completed. Investigators must assess and document the relationship between each SAE and each specific study treatment (if more than one study treatment is involved), and submit the completed and signed form to the relevant national regulatory authorities and ethics committee within 24 hours. The original SAE report form and fax confirmation must be kept in the investigator's files at the research center.

Follow-up information should be sent to the same contact who received the original SAE report form, using a new SAE report form, indicating that this is follow-up information regarding the previously reported SAE, and providing the date of the original report. Regardless of when it occurs, the recurrence, complications, or progression of each initial event should be reported as follow-up information for that event. The follow-up information should describe whether there was relief or continuation of symptoms, whether treatment was received, how the treatment was administered, and whether to continue or withdraw from the study.

### Reporting and tracking of pregnancy

#### Pregnancy in female subjects

Fertile female patients who refuse to take effective contraceptive measures are prohibited from enrolling in this trial. If a female patient becomes pregnant during the study or within 60 days after the last dose of the investigational drug, the investigator should be notified immediately. The investigator should immediately (i.e., within 24 hours of learning of the pregnancy) complete the pregnancy report form and submit it to the relevant national regulatory authorities and the ethics committee. The investigator should immediately discontinue the investigational drug and discuss the risks of pregnancy and potential effects on the fetus with the subject. Follow-up for the pregnancy will continue until three months after the estimated date of delivery to determine outcomes, including spontaneous or voluntary termination of pregnancy, details of birth, any occurrence of birth defects, congenital anomalies, or maternal and/or neonatal complications, with follow-up for the newborn lasting at least three months. Any SAE related to pregnancy (such as events in the fetus/newborn or events occurring in the mother during or after pregnancy) should be recorded on the AE page of the CRF and reported promptly.

#### Pregnancy of female partners of male subjects

If the spouse of a male subject becomes pregnant during the study or within 60 days after the last dose of the investigational drug, the investigator should be notified immediately, and the investigator must report to the relevant national regulatory authorities and ethics committee within 24 hours of learning about the pregnancy. The investigator will provide information regarding the risks of pregnancy and potential effects on the fetus, and follow-up on the pregnancy status must be conducted as much as possible.

#### Miscarriage

All spontaneous abortions should be classified as SAEs, recorded on the AE page of the CRF, and reported immediately (i.e., within 24 hours of knowledge of the event).

#### Congenital Anomalies/Birth Defects

Any congenital anomalies/birth defects in infants born to female patients or female partners of male subjects who have been exposed to the investigational drug should be classified as SAEs, recorded on the AE page of the CRF, and reported immediately (i.e., within 24 hours of knowledge of the event).

## Adverse Event Recording Procedures

When recording AEs on the AE page of the CRF, investigators should use accurate medical terminology/concepts and avoid colloquialisms and abbreviations.

In the event field of the AE page in the CRF, only one AE term can be filled in.

### Infusion-related reactions

Adverse events (AEs) that occur during the administration of the investigational drug or within 24 hours after administration and are judged to be related to the infusion of the investigational drug should be recorded as diagnostic results on the AE page of the CRF (e.g., infusion-related reactions). Ambiguous terms, such as 'systemic reactions,' should be avoided whenever possible. If a subject experiences both local and systemic reactions after administration of the same dose of the investigational drug, each reaction should be recorded separately on the AE page of the CRF.

### Diagnosis and signs and symptoms

In addition to individual signs and symptoms, the AE page of the CRF should also record diagnostic results (if known), such as recording liver failure or hepatitis, rather than jaundice, asterixis, and elevated transaminases. However, if a single diagnosis cannot be made for a set of signs and/or symptoms at the time of reporting, each event should be recorded individually on the AE page of the CRF. If a diagnosis is subsequently clarified, all events previously based on the symptoms and signs of that diagnosis should be retracted and replaced with a single AE report based on the confirmed diagnosis, with the event start date being the start date of the initial symptom of the final diagnosis.

### adverse events secondary to other events

In general, AEs that are secondary to other events (such as cascade events or clinical sequelae) should have their primary cause determined, except for severe or serious secondary events. If the secondary adverse event (AE) is clinically significant and both events occur independently in terms of timing, the secondary event should be recorded as a separate event on the AE page of the Case Report Form (CRF). For example:

- In healthy adults, if vomiting leads to mild dehydration and no other treatment is required, only vomiting should be reported on the CRF;
- If vomiting leads to severe dehydration, these two events should be reported separately on the CRF;
- If severe gastrointestinal bleeding leads to renal failure, these two events should be reported separately on the CRF;
- If dizziness leads to a fall, resulting in a fracture, it should be reported as three separate events on the CRF;
- If neutropenia leads to infection, these two events should be reported separately in the CRF.

If it is unclear whether there is a relationship between the events, all AEs should be recorded separately on the AE page of the CRF.

### Persistent or recurrent adverse events

A persistent AE refers to an AE that continues without relief between assessment time points for each subject. Such events need to be recorded only once on the AE page of the CRF. At the time of the initial report of the event, the initial severity (grade or level) of the event should be recorded. If the persistent AE worsens, the maximum severity should be recorded on the AE page of the CRF. If the AE meets the criteria for SAE, the investigator must report it immediately (within 24 hours of becoming aware of the worsening event). Update the AE page of the CRF by changing the severity of the event from 'non-serious' to 'serious', providing the date of event escalation, and filling in all data related to the SAE.

Recurrent AEs refer to AEs that have resolved at the current assessment point compared to the previous assessment point but then recur. Each recurrence of an AE should be recorded separately on the AE page of the CRF.

### Abnormal laboratory test values

Not every abnormal laboratory test value qualifies as an AE. Abnormal laboratory test results must be reported as AEs when they meet any of the following criteria:

- Accompanied by clinical symptoms;
- Leading to changes in study treatment (e.g., dose adjustment, treatment interruption, or termination of treatment);
- Requires medical intervention (e.g., potassium supplementation for hypokalemia) or changes in concomitant medication;
- The investigator considers it clinically significant.

If a clinically significant laboratory abnormality is a sign of a disease or syndrome (e.g., ALP and bilirubin > 5 ULN related to cholestasis), only the diagnosis (i.e., cholestasis) needs to be recorded on the AE page of the CRF.×

If a clinically significant laboratory test result abnormality is not indicative of a specific disease or syndrome, the abnormal result itself should be recorded on the AE page of the CRF, accompanied by a description indicating whether the result is above or below the normal range (e.g., 'hyperkalemia' instead of 'potassium abnormality'). If an abnormal laboratory test result is defined as per the standard and can be accurately described using a clinical term, that clinical term should be recorded as an AE. For example, if serum potassium rises to 7.0 mEq/L, it should be recorded as 'hyperkalemia.'

If the same clinically significant abnormal laboratory test result is observed between visits, it should only be counted once on the AE page of the CRF (for detailed information on recording persistent AEs, please refer to Section 8.8.4 ).

The investigator is responsible for reviewing all laboratory test results. For laboratory abnormalities that meet the criteria for adverse events, follow-up should continue until normalization or baseline levels are achieved and/or there is a reasonable and sufficient explanation.

Laboratory abnormalities that do not meet the definition of adverse events should not be reported as AEs. CTCAE graded events of grade 3 or 4 (severe) do not automatically qualify as SAEs unless they meet the definition of serious adverse events and/or are determined by the investigator to be serious adverse events.

### Abnormal vital sign values

Not every abnormal vital sign value qualifies as an AE; when the results of vital sign examinations meet any of the following criteria, they must be reported as AEs:

- Accompanied by clinical symptoms;
- Leading to changes in study treatment (e.g., dose adjustment, treatment interruption, or termination of treatment);
- Requiring medical intervention or changes in concomitant medication;
- The investigator considers it clinically significant.

The investigator is responsible for reviewing all vital sign examination results.

If an abnormal vital sign examination result of clinical significance indicates a certain disease or syndrome (e.g., hypertension), only the diagnosis (i.e., hypertension) needs to be recorded on the AE page of the CRF.

If the same clinically significant abnormal vital sign examination result is observed at each visit, it should not be repeatedly recorded on the AE page of the CRF unless there is a change in the underlying cause (for details on recording persistent AE, please refer to Section 8.8.4 ).

### Abnormal liver function test results

Elevated ALT or AST (3ULN) and elevated total bilirubin (2ULN) or clinical jaundice, but without cholestasis or other causes of hyperbilirubinemia, should be considered indicative of severe liver injury (as defined by the Haisch criteria). Therefore, when any of the following situations occur, the investigator must report it as an SAE:>×>×

- During treatment, if ALT or AST is 3 ULN, accompanied by total bilirubin 2 ULN;>×>×
- ALT or AST of 3 ULN occurring during treatment, accompanied by clinical jaundice.>×

The most appropriate diagnosis or laboratory abnormality (if a clear diagnosis cannot be made) will be recorded as an SAE in the AE page of the CRF and reported immediately (i.e., reported within 24 hours of becoming aware of the event).

### Death

In this study protocol, death is considered an efficacy endpoint. Deaths occurring within the AE reporting period specified in the study protocol, if the investigator determines the cause of death to be tumor progression, should be recorded in the CRF under the section for deaths attributed to disease progression. All other study-related deaths, regardless of whether they are related to the investigational drug, must be recorded on the AE page of the CRF and reported immediately.

Death should be considered an outcome rather than a separate event. Events or conditions leading to a fatal outcome should be recorded as a single medical concept on the AE page of the CRF. Generally, only one such event should be reported. The term 'sudden death' is used only for deaths that are abrupt and unexpected, with a presumed cardiac cause. If the cause of death is unknown, or cannot be determined at the time of reporting, it should be recorded as 'cause of death unknown' on the AE page of the CRF. If the cause of death is clarified later (e.g., after autopsy), 'cause of death unknown' should be changed to the clarified cause of death.

Deaths attributed to tumor progression that occur during the survival follow-up period should be recorded in the death CRF attributed to disease progression.

### Pre-existing disease condition

A pre-existing disease condition refers to a disease state that was present at the time of the screening visit for this study. This disease state should be recorded in the general medical history and baseline physical condition CRF.

During the study, an adverse event (AE) should only be recorded if the frequency, severity, or nature of a pre-existing medical condition worsens. When recording such events on the AE page of the CRF, care should be taken to use appropriate descriptions to convey the concept that an existing medical condition has changed (e.g., 'more frequent headaches').

### Adverse events related to drug overdose or administration errors

Drug overdose refers to the unintentional or intentional administration of a dose higher than the level currently being studied. Neither drug overdose nor incorrect administration of the investigational drug itself should be considered an AE, but they may lead to AEs. AEs related to drug overdose or incorrect administration of the investigational drug will be classified as SAEs, recorded on the AE page of the CRF, and reported immediately (i.e., within 24 hours of becoming aware of the event).

# Data Collection and Management

## Data Confidentiality

Information regarding research subjects will be kept confidential and managed in accordance with applicable laws and regulations.

## Central Monitoring

Before the study begins, at the central initiation meeting, the investigator and their team review the protocol, informed consent form, and CRF, among other documents. During the study, monitors will regularly visit the center to verify records and reports of SAEs, the existence of informed consent forms, compliance with inclusion/exclusion criteria, the completeness of patient records, the accuracy of CRF entries, adherence to the protocol and Good Clinical Practice guidelines, as well as the progress of patient recruitment, ensuring that the investigational drug is stored, distributed, and accounted for according to regulations. During these visits, key research personnel must be present to assist the monitors.

## Data Collection

This trial uses Case Report Forms (CRF) to collect data in clinical research, and designated researchers will enter the data into the CRF according to the protocol requirements. Researchers must retain the source documents for each subject, including original medical records and all other paper data or records. The data entered into the CRF must be traceable to the source documents in the subject files. The principal investigator is responsible for ensuring that the data entered into the CRF is complete, accurate, and updated in a timely manner.

Radiological and imaging data will be obtained by the center and interpreted at the center.

## Database Management and Quality Control

Data Entry: During the data entry process, the investigator or authorized research personnel may contact the data department at any time to communicate and resolve any questions encountered.

Data verification: The methods of data verification during the data management process include logical data verification, manual verification, medical verification, and statistical pre-analysis verification stages. Data inquiries will be generated for discrepancies and missing values, and the designated research center staff must respond to the inquiries immediately and make necessary modifications to the data. If the responses meet the requirements, the inquiry will be closed. If the data queries remain unresolved or if new queries arise after updating the database based on the previous data query responses, the researcher or their authorized personnel must provide new answers. The above process will be repeated until all data in the database is confirmed to be accurate.

The terminology from the International Medical Dictionary (MedDRA) will be used to code medical history/current medical conditions and adverse events.

## Database Lock

At the end of the study, it will be determined whether any protocol deviations occurred. After completing the above steps and confirming that the data is complete and accurate, the database will be locked, clearly defining the data available for analysis. Before the database lock, the project manager will convene a data review meeting with the principal investigator, statistician, and data manager to determine the analysis population. After the lock, the DM will submit the locked data to the statistician for statistical analysis.

The data can be locked once the following conditions are met:

- All data has been collected and entered into the database.
- All coding has been verified and confirmed.
- All data queries have been resolved (including those raised during data review).
- Database QC has passed.
- Data review has been completed.
- Verification of original documents has been completed.
- The verification of SAEs has been completed.
- All investigators' signatures have been obtained.
- The analyzable cases have been defined and saved in the final analysis database.
- The statistical analysis plan has been signed.

Prior to any database modifications on the locked data, joint written approval from the biostatistics and data management director and the clinical research director is required.

# Statistical analysis

The statistical analysis plan will be developed after the protocol is finalized and will be finalized before database lock. The statistical analysis plan will specify and describe in detail all statistical analyses to be conducted based on the main features of the protocol. All statistical analyses will be computed using SAS statistical analysis software.

This study provides statistical descriptions of all variables obtained at each observation time point, unless the protocol specifies that statistical descriptions are not required at specific time points. The results of this study primarily utilize descriptive statistical analysis. For continuous variables, standard descriptive statistical results include mean, standard deviation, median, maximum, minimum, while count data and ordinal data list frequencies (proportions), rates, and 95% confidence intervals (CI). The number of subjects enrolled, as well as dropout and exclusion cases, are described. The final analysis of the study will be based on data collected from subjects throughout the study period. Statistical methods will be detailed in the statistical analysis plan.

## Data Analysis Set

All observational indicators of the subjects are included in the statistical analysis. The dataset includes the Full Analysis Set (FAS), Safety Set (SS), and Per Protocol Set (PPS). Efficacy analysis will utilize both FAS and PPS, with FAS as the primary analysis set. Safety analysis will use SS.

**FAS:** Refers to the collection of all cases that have used the investigational drug at least once according to the protocol, excluding cases that have been removed; Cases that did not experience tumor progression during the observation and follow-up periods will have their tumor progression time treated as censored, with the censoring time being the most recent confirmation of no tumor progression.

**SS:** Refers to the population of subjects who have signed informed consent and have used the investigational drug at least once.

**PPS:** A subset of the FAS population, referring to all cases that have used the investigational drug according to the protocol for at least 2 weeks and meet the inclusion criteria for the FAS. Cases that have taken the drug for less than 2 weeks but have experienced radiologically confirmed disease progression within 12 weeks after starting the drug, and meet the inclusion criteria for the FAS, will also be included in the PPS. Cases in which tumor progression was not observed during the observation and follow-up periods will have their tumor progression time treated as censored, with the censoring time being the most recent confirmation of tumor non-progression.

Cases that are excluded should be retained for reference; except for cases that have previously used the drug being classified as SS, other cases (those that have not used the drug at all) will not be included in the statistical analysis.

## Demographic and baseline disease characteristics

Summarize the demographic and baseline disease characteristic data according to the analysis methods stated above.

## Efficacy analysis

### primary efficacy endpoints

Summarize the ORR, including the number of subjects with ORR, the percentage, and the 95% confidence interval (calculated using the Clopper-Pearson method).

### Secondary efficacy endpoints

Calculate the median and its 95% confidence interval (95% CI) for PFS, OS, TTP, and DOR using the Kaplan-Meier method.

DCR is summarized, including the number of subjects with DCR, percentage, and 95% confidence interval (calculated using the Clopper-Pearson method).

The comparison of pre- and post-treatment changes in ECOG PS score and AFP values will be conducted using paired t-test.

## Safety Analysis

A descriptive summary table will be provided for all safety parameters. Summarize adverse events and adverse reactions (defined as 'definitely related, possibly related, or indeterminate relationship to the investigational drug' AEs) that occurred during treatment according to CTCAE V5.0 grading, calculate the incidence of AEs, list the frequency and count of AEs by system, and calculate percentages, along with a detailed list of various AE cases. Analyze the occurrence of AEs, and evaluate the safety of the investigational drug in conjunction with vital signs and laboratory test results during the treatment process.

# Research management

The design, implementation, and reporting of this study will strictly adhere to the relevant laws and regulations of clinical research in China, including GCP, the Declaration of Helsinki, and other regulations, as well as the execution of this research protocol.

## Ethical considerations

The research protocol, ICF, CRF, and all other documents requiring review must be submitted to the ethics committee for approval before the study begins. The study can only commence after approval from the ethics committee. During the research process, any modifications to the protocol must be reviewed and approved by the ethics committee before they can be implemented.

Before the start of the study, the investigator is required to sign the signature page of the protocol, confirming their agreement to conduct the study in accordance with all guidelines and procedures outlined in these documents and the protocol.

## Informed Consent

Before each subject enters this study (prior to the screening examination), the investigator is responsible for providing a complete and comprehensive introduction to the subject or their legal representative regarding the background, purpose, pharmacological characteristics of the study medication, study protocol, procedures, as well as the benefits and potential risks of participating in the study, in a manner and language that the subject can understand. Written informed consent must be obtained, with the signature and date from the subject or their legal representative and the investigator conducting the informed consent process, and subjects should be made aware that they have the right to withdraw from the study at any time. The original informed consent form should be retained by both the investigator and the subject, with each keeping one copy, and it should be preserved as part of the clinical research documentation. The informed consent form should be obtained with written approval from the relevant regulatory authority and written in a language that is understandable to the subject. If important new data regarding the investigational drug is discovered during the research process, the informed consent form must be modified in writing and submitted to the ethics committee for re-approval, and informed consent from the subject must be obtained again before continuing the research.

For women of childbearing potential, it should be communicated that if pregnancy occurs during the study, the investigational drug may pose unknown risks to the fetus. Furthermore, patients must agree to adhere to contraceptive requirements during the study in order to participate. Patients who cannot reliably comply with any requirements should not enter this study.

## Compensation for Health Damage to Subjects

Subjects who suffer research-related accidental injury or death due to participation in the study will be compensated in accordance with Chinese laws and regulations. This study has obtained clinical trial liability insurance for the investigational drug. From the start of participation in this study until its conclusion, subjects will receive active treatment for any adverse events or serious adverse events related to the investigational drug.

## The preservation of research documents, records, and files

The research center will maintain appropriate medical and research records for this study in accordance with ICH E6 GCP Section 4.9 and regulatory and institutional requirements regarding the confidentiality of subjects.

Source data refers to the original records of clinical findings, observations, or other actions necessary for the reconstruction and evaluation of the study in clinical research. Examples of these original documents and data records include but are not limited to hospital records, clinic and office record forms, laboratory reports, memos, subject diaries or assessment checklists, pharmacy dispensing records, data from automated instrument records, verified accurate and complete copies or transcripts, microfilm, photographic negatives, microfiche or magnetic media, X-ray examination results, and subject documents and records stored in the pharmacies, laboratories, and medical technology departments involved in the clinical research.

Under the supervision of the principal investigator, the clinical research staff at the center is responsible for data collection. The CRF is the primary data collection tool for the study. Investigators must ensure the accuracy, completeness, legality, and timeliness of the data reported in the CRF and all other required reports. Data reported on the CRF from source documents must be consistent with the source documents, or discrepancies must be explained. All data required on the CRF must be recorded. Any missing data must be explained. For any changes or modifications on the paper CRF, the date, initials, and explanation (if necessary) should be noted, and the original entries should not be erased. For electronic CRFs, the system will maintain an audit trail. Investigators should keep records of changes and modifications to the paper CRF.

Researchers should retain all research materials, including confirmation records for all subjects (which can effectively verify all recorded data, such as CRF and original hospital records), all original signed subject ICFs, all CRFs, detailed records of drug distribution, etc. After the completion of the clinical research, unless required by applicable laws, regulations, and/or guidelines to be retained for a longer period, essential documents (both handwritten and electronic) should be kept for at least five (5) years.

All materials from this clinical research are owned by the researchers.

## Confidentiality of research documents and patient records

Researchers are responsible for maintaining the anonymity of subjects and protecting the personal privacy and data confidentiality of subjects. Subjects can only be identified in case report forms or other documents using capital letters, numbers, and/or codes, and not by their names. The signed informed consent form and the subject enrollment form must be kept strictly confidential while ensuring that the center can identify the patients. If it is necessary to identify the subjects' names for medical reasons during the research process, all relevant personnel are obligated to maintain confidentiality.

## Return or destruction of research drugs/treatment supplies

After the study concludes, all unused clinical research drugs/treatment supplies should be returned to Suzhou Zelgen or destroyed at the study site according to Suzhou Zelgen's written instructions.

The destruction of research drugs/treatment supplies at the research site must be carried out in the presence of a representative from Suzhou Zelgen or by appropriately designated personnel from Suzhou Zelgen. If the destruction of research drugs/treatment supplies is carried out by designated personnel, a formal signed destruction certificate should be filled out and submitted to Suzhou Zelgen.

## Protocol deviation

Investigators should not change or deviate from the study protocol unless it is in an emergency situation to protect the life and safety of the subjects. If investigators deviate from the study protocol in an emergency situation to protect the life and safety of the subjects, they must notify the study sponsor and the ethics committee (if applicable) of this deviation and any deviations that affect the scientific integrity of the clinical research. All protocol deviations and their reasons and dates of occurrence must be documented.

## Research Summary Report

After the conclusion of the study, the researchers will objectively summarize the findings based on the results and conduct statistical analysis of the research data using appropriate statistical methods. An objective evaluation of the drug's safety will be made based on the results, culminating in a written summary report of this clinical research.

# References

[1] Parkin D M, Bray M F, Ferlay M J, et al. Global cancer statistics, 2002.[J]. Ca A CancerJournal for Clinicians, 2005, 55(2):74.

[2] Chen W, Zheng R, Baade PD, et al. Cancer statistics in China, 2015[J]. CA: A Cancer Journal for Clinicians, 2016, 66(2).

[3] Akateh C, Black S M, Conteh L, et al. Neoadjuvant and adjuvant treatment strategies for hepatocellular carcinoma[J]. World Journal of Gastroenterology, 2019, 25(28):3704-3721.

[4] Shi-Dong Lu, Lin Li, Xin-Min Liang, et al. Updates and advancements in the management of hepatocellular carcinoma patients after hepatectomy. Expert Rev Gastroenterol Hepatol 2019, 13 (11), 1077-1088.

[5] Chen Minshan, Yuan Yunfei, Guo Rongping, et al. The Application of Hepatic Arterial Infusion Chemotherapy in the Conversion Treatment of Liver Cancer—Experience Summary from the Cancer Prevention and Treatment Center of Sun Yat-sen University [J]. Chinese Journal of Medical Frontier (Electronic Edition) 2021, Volume 13, Issue 3, Pages 70-76, ISTIC, 2021

[6] Chen Minshan, Hu Zili. Research Progress of Hepatic Arterial Infusion Chemotherapy in the Conversion Treatment of Liver Cancer[J]. Chinese Journal of Digestive Surgery, 2021, 20(02):171-177.

[7] Liu Shaoxing, Zhu Xu. Progress of Hepatic Arterial Infusion Chemotherapy in Advanced Liver Cancer[J]. Chinese Journal of Interventional Imaging and Therapy, 2020, Volume 17, Issue 10, Pages 632-635, ISTIC PKU CSCD, 2020.

[8] Han Yue, Huang Zhen, Jiang Zhichao, et al. Expert Consensus (Draft) on Targeted Therapy for Liver Cancer[J]. Electronic Journal of Liver Cancer, 2020(2).

[9] Allison J P. Immune Checkpoint Blockade in Cancer Therapy: The 2015 Lasker-DeBakey Clinical Medical Research Award[J]. Jama, 2015, 314(11):1113-4.

[10] Cooper, Zachary, A, et al. Targeted Therapies Combined With Immune Checkpoint Therapy[J]. Cancer Journal, 2016.12.

[11] Bi F , Qin S , Gu S , et al. Donafenib Versus Sorafenib in First-Line Treatment of Unresectable or Metastatic Hepatocellular Carcinoma: A Randomized, Open-Label, Parallel-Controlled Phase II-III Trial. Journal of Clinical Oncology. https://ascopubs.org/doi/full/10.1200/JCO.21.00163.

[12] Lin Tian, Amit Goldstein, Hai Wang, et al. Mutual regulation of tumour vessel normalization and immunostimulatory reprogramming[J]. Nature, 2017, 544(7649):250-254.

[13] Prieto P A, Reuben A, Cooper Z A, et al. Targeted Therapies Combined With Immune Checkpoint Therapy[J]. The Cancer Journal, 2016, 22(2):138-146.

[14] MinKe He, Shi Ming, Zhicheng Lai, et al. A phase II trial of lenvatinib plus toripalimab and hepatic arterial infusion chemotherapy as a first-line treatment for advanced hepatocellular carcinoma (LTHAIC study). J Clin Oncol. 39, 2021 (suppl 15; abstr 4083). https://meetinglibrary.asco.org/record/198801/abstract.

[15] Ti Zhang, Jinliang Zhang, Xihao Zhang, et al. Triple combination therapy comprising angiogenesis inhibitors, anti-PD-1 antibodies, and hepatic arterial infusion chemotherapy in patients with advanced hepatocellular carcinoma. J Clin Oncol 39, 2021 (suppl 15; abstr e16124). https://meetinglibrary.asco.org/record/199547/abstract.

1. **Attachment**

**Attachment 1 Trial Flowchart**

| **Research Phase** | **Screening Period (D)** | **donafenib Monotherapy Period**  **(Duration 3~7 days)** | **Combination Therapy Period**  **(Every 3 weeks constitutes one treatment cycle)** | | | | **End of Treatment** | **Post-Treatment** | | |
| --- | --- | --- | --- | --- | --- | --- | --- | --- | --- | --- |
|  |  |  | **C1D1** | **C2D1** | | **C3D1、C4D1……** | **Confirmation within 7 days after end/discontinuation of treatment** | **Safety follow-up** | **Efficacy Follow-up** | **Survival follow-up** |
|  | **-14 ~ -0** | **D1** | **±3D** | **±3D** | | **±3D** |  | **30±7 days after last administration** | **Every 12 weeks or 24 weeks ±7 days** | **Every 12 weeks**  **±7D** |
| Informed Consent **^1^** | × |  |  |  | |  |  |  |  |  |
| Demographic Information ^2^ | × |  |  |  | |  |  |  |  |  |
| Smoking History, Alcohol Consumption History | × |  |  |  | |  |  |  |  |  |
| Past Medical History and Treatment History | × |  |  |  | |  |  |  |  |  |
| Previous tumor history and treatment history, including postoperative pathology | × |  |  |  | |  |  |  |  |  |
| Physical examination **^3^** | × | × |  | × | | × | × | × |  |  |
| Height measurement **^4^** | × |  |  |  | |  |  |  |  |  |
| Weight measurement **^5^** | × | × |  | × | | × | × | × |  |  |
| ECOG PS score **^6^** | × | × |  | × | | × | × | × | × |  |
| Vital signs **^7^** | × | × | × | × | | × | × | × |  |  |
| Child-Pugh score | × |  |  | × | | × | × |  |  |  |
| BCLC liver cancer staging | × |  |  |  | |  |  |  |  |  |
| Complete blood count, blood biochemistry, urinalysis, coagulation function **^8^** | × | × |  | × | | × | × | × |  |  |
| Fecal occult blood | × | Only performed when there are clinical indications or deemed necessary by the investigator | | | | | | |  |  |
| Pregnancy test **^9^** | × |  |  |  | |  | × |  |  |  |
| Cardiac ultrasound **^10^** | × | Only performed when there are clinical indications or deemed necessary by the investigator | | | | | | |  |  |
| 12-lead electrocardiogram | × | × |  | × | | × | × | × |  |  |
| Virology testing **^11^** | × |  |  | | Known HBV positive patients should be tested for HBV DNA,  HCV RNA testing for known HCV positive patients; | | | |  |  |
| Thyroid function **^12^** | × |  |  | | × | × | × | × |  |  |
| AFP **^13^** | × |  |  | × | | × | × | × |  |  |
| Tumor assessment (CT/MRI) **^14^** | × |  | Once every 6 weeks ± 3 days (relative to C1D1) | | | | × |  | × |  |
| Inclusion/Exclusion criteria  Confirmation | × |  |  |  | |  |  |  |  |  |
| Dispensing of donafenib **^15^** |  | × | × | × | | × |  |  |  |  |
| Assessment of patient medication adherence **^16^** |  |  | × | × | | × | × |  |  |  |
| Administration of donafenib treatment |  |  | | | | |  |  |  |  |
| Administration of Sintilimab treatment |  |  | × | × | | × |  |  |  |  |
| Administration of HAIC treatment ^17^ |  |  | × | To be assessed by the investigator based on actual conditions as needed | | |  |  |  |  |
| DLT observation and assessment ^18^ |  |  | | | |  | | | | |
| Concomitant medication/Concomitant treatment **^19^** |  | | | | | | | |  | |
| Adverse event collection ^2^ **^0^** |  | | | | | | | |  | |
| Collection of new antitumor treatment information |  |  |  |  | |  | × | × | × | × |
| Survival status |  |  |  |  | |  |  |  |  | × |

**Note:**

1. The examination results conducted at this research center before the patient signs the informed consent form (within 14 days prior to screening assessment) can be used to evaluate the eligibility of subjects and serve as the baseline for enrolled subjects. However, all subjects must sign the ICF in writing before any non-routine trial-related procedures are performed;
2. Demographic information includes: date of birth, gender, race/ethnicity;
3. Physical examination includes head, eyes, ears, nose, throat, neck, heart, chest (including lungs), abdomen, limbs, skin, lymph nodes, nervous system, and general condition of the subjects;
4. Height measured only during the screening period;
5. Weight measured during the screening period, before each cycle of PD-1 monoclonal antibody administration, and at the end of treatment/early withdrawal visit;
6. ECOG PS score does not need to be repeated on Day 1 of donafenib administration if it was conducted within 3 days prior to the first dose;
7. Vital signs include: temperature, respiration, blood pressure, and heart rate;
8. If the screening period's blood routine, blood biochemistry, urine routine, coagulation function, and electrocardiogram examinations were conducted within 3 days prior to the first administration, the corresponding examinations on Day 1 of donafenib administration may be omitted, and the results from the screening period can be used as baseline data. Safety checks during the combination treatment phase CIDI do not need to be repeated unless the subject has corresponding clinical indications or the investigator deems it necessary. Safety checks for C2D1 and subsequent cycles must be completed before each combination medication cycle, and the blood sampling time cannot be earlier than 3 days prior to medication; the results must be determined by the investigator to meet the criteria for continuing medication before medication can begin.
9. Conducted only in female subjects of childbearing potential. Women of childbearing age will undergo urine or serum pregnancy tests within 3 days prior to the first administration and at the end of treatment follow-up. If the urine pregnancy test result cannot be confirmed as negative, a serum pregnancy test is required, and the serum pregnancy result shall prevail;
10. Cardiac ultrasound does not need to be repeated on Day 1 of donafenib treatment; the results from the screening period can be used as baseline data, unless the investigator deems it necessary to perform it; Subsequently, it will only be performed if the subject has clinical indications (such as ECG abnormalities, chest tightness, cyanosis, dyspnea, etc.) or if the investigator considers it necessary;
11. Virological testing does not need to be repeated on Day 1 of the monotherapy period of donafenib and Day 1 of the combination therapy period; thereafter, HBV DNA will only be tested in known HBV-positive patients, and HCV RNA will be tested in known HCV-positive patients;
12. Thyroid function does not need to be rechecked on Day 1 of the monotherapy phase with donafenib and on Cycle 1 Day 1 of the combination therapy phase; if there are clinically significant changes in thyroid function during treatment, it is recommended to consult the endocrinology department and rule out pituitary dysfunction.
13. AFP does not need to be rechecked on Day 1 of the monotherapy phase with donafenib and on Cycle 1 Day 1 of the combination therapy phase.
14. During the study period, the same imaging techniques (scanning instruments and methods as well as imaging parameters) used at baseline must be employed, measurement methods must remain consistent with baseline, and evaluations should ideally be conducted by the same investigator. The areas to be assessed include the chest and abdomen; if there are clinical indications or suspicion of metastasis in other areas, CT/MRI examinations should be performed. If the investigator finds that there is a possibility of disease progression in the subject, an unscheduled CT/MRI examination may be conducted. If the unscheduled examination does not reveal progression, subsequent evaluations should be conducted as closely as possible to the originally scheduled examination time. If the subject confirms disease progression, no further CT/MRI examinations will be required.
15. During the monotherapy period of donafenib on Day 1, after calculating the number of doses for the subject based on the determined dosage, the corresponding quantity of medication will be dispensed to the subject. Subsequently, during each combination therapy period on Day 1, there is no need to retrieve any unused medication from the patient; the remaining medication can continue to be taken, and only the quantity of medication required for this treatment cycle needs to be supplemented.
16. After administering the first cycle of donafenib to the subjects on Day 1 of the monotherapy treatment period, subsequent inquiries will be made on Day 1 of each combination treatment period and at the end of the treatment period regarding the amount of medication taken in the previous cycle and the remaining amount in hand, in order to assess whether the patient's medication adherence in the previous cycle meets the requirements;
17. After the first HAIC treatment, the total number of subsequent HAIC treatments will be determined as needed by the investigator based on the actual situation of the subjects;
18. DLT assessment is applicable only to patients entering the safety induction phase of the study (i.e., the first 6 patients enrolled), with the observation and assessment period being: from the first administration of donafenib treatment to 21±3 days after the first HAIC treatment;
19. From 28 days prior to enrollment, all medications/treatments received by the subjects must be recorded in the CRF, including the generic name of the drug, administration dose, frequency, route, the reason for using the drug/treatment, and the start and end dates. Any new changes in medication treatment must be continuously updated;
20. The collection of adverse events (AEs) begins from the signing of the ICF until 30 days after the last administration or the start of a new antitumor treatment, whichever occurs first. Adverse signs, symptoms, or medical conditions that existed at the time of signing the informed consent form should be recorded as medical history;

# Appendix 14

**Appendix I: Common Toxicity Standards (CTC) Grading (5.0) by the National Cancer Institute (NCI)**

For complete standards, please refer to the NCI CTCAE v5.0 or the online information at the following NCI website: https://ctep.cancer.gov/protocolDevelopment/electronic_applications/ctc.htm.

**Appendix II: Efficacy Evaluation Criteria for Solid Tumors**

Efficacy evaluation criteria for solid tumors version 1.1 (RECIST v1.1): As there is currently no official Chinese version of RECIST v1.1 published, this version is an internal translation. For more detailed content, please refer to the English version at http://ctep.cancer.gov/protocolDevelopment/docs/recist_guideline.pdf.

**Abstract**

**Background Introduction**

Evaluating changes in tumor burden is an important characteristic of clinical assessment in cancer treatment. Tumor shrinkage (objective response) and disease progression are both meaningful judgment endpoints in clinical trials. Since the publication of RECIST in 2000, many researchers, associations, companies, and government authorities have adopted this standard to evaluate treatment efficacy. However, some issues that have emerged subsequently have led to the publication of this revised version (version 1.1). The revisions (see the topics in each chapter) stem from evaluations of large databases (over 6,500 patients), simulation studies, and literature reviews.

**Key revisions in version 1.1 of RECIST**

The main revisions are:

Determination of the number of lesions: For the convenience of analysis, the evaluation of many trial data will be consolidated into a single database. According to this database, the total number of lesions required to assess the response endpoint tumor burden has been reduced from a maximum of 10 to a maximum of 5 (with a maximum of 5 per organ reduced to 2).

The determination of pathological lymph nodes is now consolidated to: nodules with a short axis value of 15 mm are considered measurable target lesions for evaluation. When assessing tumor efficacy, the short axis value of the (nodular) lesions must be included in the total sum of the lesions (radius). Nodules are considered normal when the short axis value shrinks to < 10 mm.

In clinical trials where response rate is the primary endpoint, efficacy needs to be confirmed; however, this is no longer necessary in randomized controlled trials, as the control group has become an effective means of interpreting trial data. The progression of the disease is elaborated in the following aspects: in addition to the original definition—an increase of 20% in the sum of target lesions (radius)—if the total number is very small, to prevent overestimation of the degree of deterioration, there must currently be an absolute increase of 5 mm in the short axis of the lesions. In addition, guidelines regarding the definition of 'definite progression' for non-measurable or non-target lesions are also provided—specifically, areas that may be confusing in the original RECIST guidelines. Finally, there is a section dedicated to the detection of new lesions, including an explanation of the FDG-PET scan results. Imaging Guidelines: The revised RECIST includes a new imaging appendix with updated recommendations for the best anatomical assessment of lesions.

Next Steps:

A key issue considered by the working group when revising RECIST v1.1 was whether it is appropriate to modify the assessment of tumor burden from a one-dimensional anatomical evaluation to a three-dimensional anatomical evaluation or to functional assessments made using PET and MRI. The current conclusion is that there is a lack of sufficient standards or evidence to abandon anatomical assessment of tumor burden. The only explanation for this is the use of FDG-PET imaging as an auxiliary means of assessing disease progression. As discussed in detail in the topic of the last chapter, the use of these latest and promising technologies requires corresponding clinical validation studies.

Keywords: Efficacy assessment criteria; solid tumors; guidelines

1. **Background**
   1. **History of RECIST criteria**

Evaluating changes in tumor burden is an important characteristic of clinical assessment in cancer treatment. Tumor shrinkage (objective response) and time to disease progression are both important endpoints in cancer clinical trials. Years of research evidence support tumor shrinkage as an endpoint for phase II trials in the screening of new anti-tumor drugs. These studies suggest that drugs which induce tumor shrinkage in some patients with various solid tumors may potentially (albeit imperfectly) be confirmed to improve overall survival or provide other opportunities for event evaluation in randomized phase III trials. Currently, among the indicators used to evaluate treatment efficacy in phase II screening trials, objective response is more reliable than any other biomarker. Moreover, in phase II and III clinical trials for drug development, clinical trials under severe conditions are gradually utilizing progression-free survival (PFS) as an endpoint for determining efficacy, which is also based on anatomical measurements of tumor size.

However, the objective response and time to disease progression, these two tumor assessment endpoints are only valuable when established on widely accepted and easily applicable standard criteria based on anatomical tumor burden. In 1981, the World Health Organization (WHO) first published the tumor response criteria, primarily for trials where tumor response is the primary endpoint. The WHO criteria introduced the concept of overall assessment of tumor burden by measuring the two-dimensional size of lesions and summing them, judging the response to treatment by evaluating changes from baseline during the treatment period. However, in the decades following the publication of this standard, collaborative groups and pharmaceutical companies that utilized it often modified it to accommodate new technologies or clarified ambiguous points in the original literature, leading to confusion in the interpretation of trial results. In fact, the application of various response standards has resulted in significantly different treatment effects for the same therapeutic approach. In response to these issues regarding reactions, an international working group was established in the mid-19th century to standardize and simplify response standards.

The new standard, also known as RECIST (Response Evaluation Criteria in Solid Tumors), was published in 2000. The initial key features of RECIST include the determination of measurable minimum lesion size, a description of the number of follow-up lesions (up to 10; a maximum of 5 per organ), the use of one-dimensional rather than two-dimensional measurements, and an overall assessment of tumor burden. These criteria were later widely adopted by academic groups, collaborative groups, and the pharmaceutical industry, with the initial endpoint of this standard being objective response or disease progression. Additionally, authorities accepted RECIST as an appropriate standard for these evaluations.

1. **Purpose of the guidelines:**

These guidelines describe a standard method for measuring solid tumors and elaborate on the objective criteria for assessing changes in tumor size used in clinical trials for adult and pediatric cancers. It is anticipated that these criteria will be used for all trials with objective response as the primary endpoint, as well as for trials employing stable disease assessment, tumor progression, or time to progression as indicators, since all measures of treatment effect are based on the evaluation of anatomical tumor burden and its changes in the study. This article does not hypothesize the proportion of patients meeting the inclusion criteria, all of whom utilize trial endpoints that can predict the efficacy of a drug or treatment regimen: those definitions depend on the type of cancer in the ongoing trial and the specific drug under investigation. The trial protocol must include an appropriate statistical section that defines the sample size of the trial and the validity parameters based on the inclusion criteria. In addition to providing definitions and criteria for assessing tumor response, this guideline also offers recommendations for the standard reporting of clinical trial results that use tumor response as an endpoint.

Although these guidelines can be used for research on malignant brain tumors, separate standards for the assessment of response have been published in this field. Since the international criteria for lymphoma response assessment have also been published separately, this guideline is not intended for research on malignant lymphomas.

Finally, many oncologists rely on multiple imaging studies in their daily clinical practice to track patients' malignant diseases and decide on further treatment plans based on both objective and symptomatic dual criteria. These RECIST guidelines will only play an important role in decision-making when deemed reasonable by the treating oncologist.

1. **Baseline Tumor Measurement**

**3.1 Definition**
At baseline, tumor lesions/lymph nodes are classified as measurable and non-measurable as follows

**3.1.1 Measurable**
**Tumor lesions:** At least one dimension must be accurately measured to be no less than the lower limit of detection (as per the measuring instrument) (the longest diameter on the measuring instrument will be recorded):
•10 mm using CT scan (with a CT scan slice thickness not exceeding 5 mm).
•Clinical examination measuring 10 mm with calipers (lesions that cannot be accurately measured with calipers should be recorded as unmeasurable).
•20 mm using chest X-ray.
**Malignant lymph nodes**: When assessed using CT scan (with a recommended CT scan slice thickness not exceeding 5 mm), the short axis of the lymph node must reach 15 mm to be considered pathologically enlarged and measurable. Only the short axis length should be measured and tracked in preoperative and follow-up work. Information regarding lymph node measurements can also be obtained from the annotations under the 'Preoperative Documents for Target and Non-target Lesions.'

**3.1.2 Non-measurable (tumor)**

All other lesions, including small lesions (with a longest diameter of less than 10 mm or pathological lymph nodes with a short axis of 10 mm to less than 15 mm), as well as truly non-measurable lesions. Lesions considered truly non-measurable include: meningeal disease determined by pharmacological examination, ascites, pleural or pericardial effusion, inflammatory breast disease, skin or lung involvement by lymphangitis, abdominal masses/organomegaly, all of which cannot be measured using reproducible imaging techniques.
**3.1.3 Special Considerations for Measurable Lesions**
Special attention is required for bone lesions, cystic lesions, and lesions that have previously undergone local treatment:
Bone Lesions:

- Bone scans, PET scans, or plain films are considered inadequate imaging techniques for measuring bone lesions. However, pharmacologically, these techniques can be used to confirm the presence or absence of bone lesions.
- If the soft tissue component meets the aforementioned criteria for measurability, osteolytic lesions with identifiable soft tissue or mixed acute osteolytic lesions can be assessed using cross-sectional imaging techniques such as CT or MRI, and they may be considered measurable lesions.
- Osteolytic lesions are unmeasurable.

Cystic lesions:

- Simple cysts defined by X-ray that meet the inclusion criteria should not be considered malignant lesions (neither measurable nor unmeasurable), as by definition, they are simple cysts.
- If the definition of measurable lesions is met, cystic lesions presenting as cystic metastases may be considered measurable lesions. However, if there are non-cystic lesions present in the same patient, priority should be given to the target lesions.

Lesions previously treated locally:

- Tumor lesions located in previously irradiated areas or those subjected to other local treatments are generally not considered measurable unless it has been demonstrated that the lesions are still ongoing. The research protocol should specify under what conditions such lesions will be considered measurable.

**3.2 Measurement Method Specifications
3.2.1 Measurement of Lesions**

Clinical assessments are measured using calipers, with all measurements recorded in metric units. All baseline assessments must be conducted as close to the start of treatment as possible, and no earlier than four weeks.

**3.2.2 Measurement Methods**

The same assessment methods and techniques should be used to describe each reported lesion at baseline and follow-up stages. Unless follow-up indicates that the lesions are unsuitable for imaging detection, imaging assessments should typically be used rather than clinical examinations.

Clinical lesions: Only superficial lesions with a diameter greater than 10 mm (such as subcutaneous nodules) measured with calipers are considered measurable. For cases of skin lesions, it is recommended to document with color photographs, with the photographs including a scale for measuring the size of the lesions. As previously mentioned, when lesions can be assessed by both clinical examination and imaging, imaging should be used, as imaging assessment is more objective and can be utilized for the final review in clinical research.

Chest X-ray examination: Chest CT is preferred over chest X-ray examination, especially when disease progression is used as an important endpoint, because CT scans are more sensitive than X-ray examinations in identifying new lesions. However, if the lesions shown on the X-ray examination have clear boundaries and are surrounded by inflated lung, they are considered measurable.

CT, MRI: CT is currently the most effective and reproducible method for assessing the efficacy of lesions. Guidelines define measurable lesions using CT scans based on a slice thickness of no more than 5 mm. As shown in Appendix II, when the CT slice thickness exceeds 5 mm, the minimum size of the lesion should be at least twice the slice thickness. MRI may also be used in certain situations (such as whole-body scans). For more opinions on the use of CT and MRI in assessing the efficacy of solid tumors, see Appendix II.

Ultrasound examination: Ultrasound is not suitable for assessing lesion size and should not be used for measurement methods. Ultrasound examinations cannot be fully reproduced between two adjacent observations, and the results depend on the examiner; from one examination to the next, the same technique and measurement results cannot be guaranteed. If new lesions are discovered through ultrasound during the study, it is recommended to verify them with CT or MRI. If there are concerns about the radiation exposure from CT, MRI can be used as an alternative to detect the lesions under investigation.

Endoscopy and laparoscopy: These techniques are not recommended for objective tumor assessment. However, they are beneficial when using biopsy to confirm complete pathological response or to determine complete response or recurrence after surgical resection.

Tumor markers: Tumor markers should not be used alone to assess objective tumor response. However, when tumor markers begin to exceed the normal upper limit, if used to determine complete response in patients, the markers must be standardized. Because tumor markers have disease specificity, the measurement technique description should indicate the records for baseline testing of a specific disease. Special guidelines regarding changes in CA-125 (in ovarian cancer recurrence) and changes in PSA (in prostate cancer recurrence) have been published. In addition, the International Gynecologic Oncology Group (InterCohort) has established progression criteria for CA-125, which will be applied to the overall first-line objective evaluation of tumors in ovarian cancer trials.

Cytology and histology: These techniques can often be used to distinguish between PR and CR in individual cases (e.g., residual benign tumor lesions in cases where the tumor type is germ cell tumor) if required by the clinical research protocol. When the exudate is known to be a potential serious adverse consequence of treatment (such as certain taxane-based chemotherapeutics or angiogenesis inhibitors), it is important to distinguish between treatment efficacy (e.g., stable disease) and disease progression. Even if measurable tumors meet the criteria for efficacy or stability, attention must be paid to any new exudate that appears or worsens during treatment, which is confirmed by cytology.

**4. Tumor Efficacy Assessment**

**4.1 Assessment of All Tumors and Measurable Lesions**

To evaluate objective response or potential future progression, it is necessary to conduct a baseline assessment of the total tumor burden for all tumor lesions, serving as a reference for subsequent measurement results. In clinical protocols where objective response is the primary treatment endpoint, only patients with measurable lesions at baseline are eligible for inclusion. Measurable lesions are defined as the presence of at least one measurable lesion. For trials where disease progression (time to progression or degree of progression at a fixed date) is the primary treatment endpoint, the inclusion criteria must clearly specify whether it is limited to patients with measurable lesions or if patients without measurable lesions can also be included.

**4.2 Baseline Recording of Target and Non-Target Lesions**

When there are more than one measurable lesions at baseline assessment, all lesions should be recorded and measured, with a total not exceeding 5 (no more than 2 from each organ), representing target lesions for all affected organs (that is, patients with only one or two affected organs may select a maximum of two or four target lesions for baseline measurement).

Target lesions must be selected based on size (longest diameter), capable of representing all affected organs, and measurements must demonstrate good reproducibility. Sometimes, when the largest lesion cannot be measured reproducibly, a new largest lesion that can be measured reproducibly may be selected.

Lymph nodes require special attention as they are normal tissue and can be detected by imaging even in the absence of tumor metastasis. Pathological lymph nodes defined as measurable nodules or target lesions must meet the following criteria: CT measurement of the short diameter ≥ 15 mm. Only the short diameter needs to be assessed at baseline. Radiologists typically use the short diameter of the nodule to determine whether it has metastasized. Nodule size is generally represented using two-dimensional data from imaging (CT uses axial planes, while MRI selects one plane from axial, sagittal, or coronal views). The minimum value is considered the short diameter. For example, a 20 mm × 30 mm abdominal nodule with a short diameter of 20 mm can be considered a malignant, measurable nodule. In this case, 20 mm is the measurement value of the nodule. Nodules with a diameter ≥ 10 mm but < 15 mm should not be considered target lesions. Nodules < 10 mm do not fall within the pathological nodule category and do not need to be recorded or further observed.

The sum of the diameters of all target lesions calculated (including the longest diameter of non-nodular lesions and the short diameter of nodular lesions) will be reported as the baseline diameter total. If lymph nodes are included, as mentioned above, only the short diameter will be counted. The baseline diameter total will serve as a reference value for the disease baseline level.

All other lesions, including pathological lymph nodes, can be considered non-target lesions and do not need to be measured, but should be recorded during the baseline assessment. Records may indicate 'present', 'absent', or in rare cases, 'definite progression'. Widely present target lesions may be recorded together with target organs (e.g., extensive pelvic lymph node enlargement or massive liver metastases).
**4.3 Efficacy Assessment Criteria**

This section defines the criteria used to determine the objective response rate (ORR) of target lesions.
**4.3.1 Efficacy Evaluation of Target Lesions**

Complete Response (CR): All target lesions disappear, and the short axis value of any pathological lymph nodes (regardless of whether they are target lesions) must be < 10 mm.

Partial Response (PR): A decrease of at least 30% in the sum of the diameters of all target lesions, relative to the baseline total diameter.

Disease Progression: An increase of at least 20% in the sum of the diameters of all target lesions, using the minimum diameter of lesions as a reference (including the baseline total diameter of lesions, if it is the minimum value). Additionally, in addition to the relative increase of 20% in the sum of diameters, the absolute value of the sum must also increase by at least 5 mm (Note: The appearance of one or more new lesions can also be considered disease progression).

Stable Disease (SD): Using the sum of the minimum diameters of lesions during the study as a reference, lesions that shrink do not meet the criteria for PR, and lesions that increase do not meet the criteria for disease progression.
**4.3.2 Considerations for Evaluating the Efficacy of Target Lesions**
When the target lesion is a lymph node:

The actual short-axis measurement value should typically be recorded (in the same anatomical plane as the baseline measurement), even if all lymph nodes in the study regress to below 10 mm. This means that when the target lesion is a lymph node, even if the criteria for complete response are met, the sum of the diameters of the target lesions will not be 0, because lymph nodes with a short-axis value < 10 mm are defined as normal lymph nodes. Case report forms or other data collection methods may need to separately record nodular target lesions at the design stage; to determine whether there is a complete response, each nodule must achieve a short-axis value < 10 mm. For PR, SD, and disease progression, the sum of the target lesions (in diameter) will include the actual short axis values of the nodules.
Lesions that are too small to measure:

All lesions (nodular and non-nodular) recorded at baseline in the study must have their actual measurements documented in subsequent assessments, even if they are very small (e.g., 2 mm).

However, sometimes lesions or lymph nodes may be recorded at the threshold value because the signal is too weak during the CT scan, and the radiologist may be reluctant to provide an exact measurement, instead reporting it as 'too small to measure'.
It is important to record a measurement in the case report form when this situation occurs. If the radiologist believes that the lesion may disappear, the measurement can be recorded as 0mm. If the lesion is indeed present but the signal is too weak, it can be recorded as the default value of 5mm (this rule is not very applicable to lymph nodes, as normal lymph nodes have a clear size value and are often surrounded by adipose tissue, such as lymph nodes in the retroperitoneal cavity; However, if the lymph node is indeed present but the signal is too weak to measure, it can also be recorded as the default value of 5mm).

The default value of 5mm is derived from the thickness of CT scan slices (if this thickness changes, the default value of 5mm should still not be altered). The measurement of lesions that are too small to be measured may lack reproducibility; providing a default value can prevent misclassification as a false cure or false progression when measurement errors occur. It is reiterated that if the radiologist can provide an actual measurement, even if it is less than 5mm, it should be recorded.
Lesions that undergo rupture or fusion during treatment

According to the notes in Appendix II, when non-nodular lesions 'fragment,' the longest diameter of all fragments must be summed to calculate the total diameter of the target lesion. Similarly, when lesions merge, the longest diameter between them can be preserved, which helps to obtain the maximum diameter value of each lesion before merging. If the lesions completely merge and are no longer separated from each other, in this case, the vector of the longest diameter is the maximum longest diameter of the merged lesions.

**4.3.3 Assessment of Non-target Lesions**

This section defines the criteria for the response of non-target lesions. Although some non-target lesions are actually measurable, measurement is not required; qualitative assessment at the time points specified in the protocol is sufficient.

Complete Response (CR): All non-target lesions disappear, and tumor markers return to normal levels. All lymph nodes are of non-pathological size (short axis < 10 mm).

Partial Response (non-CR)/Stable Disease: One or more non-target lesions are present and/or tumor marker levels remain above normal.

Disease Progression: Existing non-target lesions show clear progression. Note: The appearance of one or more new lesions is also considered disease progression.

**4.3.4 Special Considerations for the Assessment of Non-Target Lesion Progression**

The following is a supplementary explanation regarding the definition of progression of non-target lesions: When a patient has measurable non-target lesions, even if the target lesions are assessed as stable or partially responding, a clear definition of progression based on the non-target lesions must meet the criterion that the overall deterioration of the non-target lesions has reached a level that necessitates the termination of treatment. Moreover, a general increase in the size of one or more non-target lesions is often insufficient to meet the criteria for progression; therefore, in cases where the target lesions are stable or partially responding, it is exceedingly rare to define overall tumor progression solely based on changes in non-target lesions.

When all non-target lesions of the patient are unmeasurable: This situation may occur in some Phase III trials when the inclusion criteria do not specify that measurable lesions must be present. Overall assessment still refers to the above standards, but there are no measurable data for lesions in this situation. The deterioration of non-target lesions is not easy to assess (by definition: all non-target lesions must indeed be unmeasurable). Therefore, when changes in non-target lesions lead to an increase in overall disease burden equivalent to the progression of target lesions, a clear definition of progression based on non-target lesions requires the establishment of an effective detection method for evaluation. For example, an increase in tumor burden is described as a volume increase of 73% (equivalent to a 20% increase in the diameter of measurable lesions). Additionally, peritoneal effusion may range from 'trace' to 'massive'; lymphatic involvement may progress from 'localized' to 'widely disseminated'; or it may be described in the protocol as 'sufficient to necessitate a change in treatment approach.'Examples include pleural effusion ranging from trace to massive, lymphatic involvement spreading from the primary site to distant sites, or it may be described in the protocol as 'necessitating a change in treatment.'If clear progression is observed, the patient should be considered to have disease progression at that point overall. It is best to have objective criteria applicable to the assessment of unmeasurable lesions; note that the additional criteria must be reliable.

**4.3.5 New Lesions**

The emergence of new malignant lesions indicates disease progression; therefore, some evaluations targeting new lesions are very important. Currently, there are no specific criteria for imaging detection of lesions; however, the discovery of a new lesion should be clear. For example, progression cannot be attributed to differences in imaging techniques, changes in imaging morphology, or other lesions outside the tumor (e.g., some so-called new bone lesions are merely the resolution of the original lesion or a recurrence of the original lesion). It is very important when the patient's baseline lesions show partial or complete response; for example, necrosis of a liver lesion may be classified as a new cystic lesion in the CT report, when in fact it is not.

Lesions detected during follow-up that were not found in the baseline examination will be considered new lesions and indicate disease progression. For example, in a patient with visceral lesions found during the baseline examination, if metastatic lesions are discovered during a CT or MRI of the head, the patient's intracranial metastatic lesions will be regarded as evidence of disease progression, even if a head examination was not performed during the baseline assessment.

If a new lesion is unclear, for example due to its small size, further treatment and follow-up evaluation are required to confirm whether it is a new lesion. If repeated examinations confirm it as a new lesion, the time of disease progression should be calculated from the time of its initial discovery.

Lesions undergoing FDG-PET assessment generally require additional tests for supplementary confirmation; it is reasonable to evaluate progression by combining the results of FDG-PET and supplementary CT examinations (especially for new suspicious diseases). New lesions can be clarified through FDG-PET examination, executed according to the following procedure:

The baseline FDG-PET scan result is negative, and the subsequent follow-up FDG-PET scan is positive, indicating disease progression.

No baseline FDG-PET scan was performed, and the results of the subsequent FDG-PET scan are positive:

If the new lesions found in the follow-up FDG-PET positive scan correspond with the results from the CT scan, it confirms disease progression.

If the new lesions found in the follow-up FDG-PET positive scan are not confirmed by the CT scan results, a repeat CT scan is required for confirmation (if confirmed, the time of disease progression is calculated from the initial abnormal FDG-PET scan).

If the positive results of the follow-up FDG-PET scan correspond with the lesions already present on the CT scan, and there is no progression of the lesions on imaging, then the disease is considered stable.

**4.4 Evaluation of Best Overall Efficacy**

The evaluation of best overall efficacy is the best efficacy record from the start of the trial to its conclusion, while considering any necessary conditions for confirmation. Sometimes, efficacy responses occur after the end of treatment; therefore, the protocol should clarify whether efficacy evaluations after treatment are included in the best overall efficacy evaluation. The protocol must specify how any new treatment prior to progression affects the best efficacy response. The best efficacy response in patients primarily depends on the results of target lesions, non-target lesions, and the performance of new lesions. Additionally, it relies on the nature of the trial, protocol requirements, and outcome measurement standards. Specifically, in non-randomized trials, the efficacy response is the primary objective, and confirmation of efficacy through PR or CR is necessary to determine which represents the best overall efficacy response.

**4.4.1 Time Point Response**

It is assumed that efficacy responses will occur at specific time points for each protocol. Appendix 1 will provide a summary of the overall efficacy response at each time point for a patient population with measurable disease at baseline.

If the patient has no measurable lesions (no target lesions), the assessment can be referred to in Appendix 2.

**4.4.2 Explanation of Missing and Non-evaluable Assessments**

If imaging or measurement of lesions cannot be performed at a specific time point, the patient cannot be evaluated at that time point. If only a portion of the lesions can be evaluated in an assessment, this situation is generally considered non-evaluable at that time point, unless there is evidence to confirm that the missing lesions will not affect the efficacy response evaluation at the specified time point. This situation is likely to occur in cases of disease progression. For example: a patient has a total of 3 lesions measuring 50 mm at baseline, but subsequently only 2 lesions are evaluable, totaling 80 mm; the patient will be assessed as having disease progression, regardless of the impact of the missing lesions.

**4.4.3 Best Overall Response: All Time Points**

Once all patient data is available, the best overall response can be determined.

Assessment of best overall response when confirmation of complete or partial efficacy response is not required: The best efficacy response in the trial is the best response at all time points (for example: a patient is evaluated as SD in the first cycle, PR in the second cycle, and disease progression in the last cycle, but their best overall response is evaluated as PR). When the best overall response is evaluated as SD, it must meet the minimum duration specified in the protocol from baseline. If the minimum duration criteria are not met, even if the best overall response is rated as SD, it will not be recognized; the patient's best overall response will depend on subsequent evaluations. For example: a patient rated SD in the first cycle and disease progression in the second cycle, but did not meet the minimum duration requirement for SD, will have their best overall response rated as disease progression. The same patient who is rated as SD in the first cycle and then lost to follow-up will be considered unassessable.

When the study requires confirmation of complete or partial efficacy response, the assessment of best overall response: it can only be declared as complete or partial response if each subject meets the criteria for partial or complete response as specified in the trial protocol and is confirmed for efficacy at a subsequent time point (generally four weeks later) as specifically mentioned in the protocol. In this case, the best overall response is described in Appendix 2.

**Special Notes on Efficacy Assessment 4.4.4**

When nodular lesions are included in the overall target lesion assessment, and the size of the lesion reduces to 'normal' size (< 10 mm), they will still have a lesion size scan report. To avoid overestimating the situation reflected by the increase in lesion size, even if the lesion is normal, the measurement results will still be recorded. As previously mentioned, this means that subjects with a complete response will not be recorded as 0 on the CRF.

If efficacy confirmation is required during the trial, repeated 'non-measurable' time points will complicate the assessment of the best efficacy. The analysis plan of the trial must clarify how these missing data/evaluations can be interpreted when determining efficacy. For example, in most trials, a subject's response of PR-NE-PR can be considered as confirmation of efficacy.

When a subject experiences an overall deterioration in health that necessitates stopping treatment, but there is no objective evidence to support this, it should be reported as symptomatic progression. Efforts should still be made to assess objective progression even after treatment has been discontinued. Symptomatic deterioration is not a description of objective response assessment: it is the reason for stopping treatment. The objective response of such subjects will be assessed through the conditions of target and non-target lesions as shown in Appendices 1 to 3.

Conditions defined as early progression, early death, and unassessable situations are exceptions in the study and should be clearly described in each protocol (depending on the treatment interval and treatment cycle).

In some cases, it is difficult to distinguish local lesions from normal tissue. When the assessment of complete response is based on such definitions, we recommend performing a biopsy prior to the efficacy evaluation of complete response in local lesions. When some subjects have abnormal imaging results of local lesions that are considered indicative of lesion fibrosis or scar formation, FDG-PET is used as an assessment standard similar to biopsy for confirming complete response. In this case, the application of FDG-PET should be prospectively described in the protocol, supported by reports from specialized medical literature addressing this situation. However, it must be recognized that the inherent limitations of both FDG-PET and biopsy (including the varying resolutions and sensitivities of the two) will lead to false positive results in the assessment of complete response.

**Appendix 1 Time Point Response: Subjects with Target Lesions (Including or Excluding Non-Target Lesions)**

| **Target Lesion** | **Non-Target Lesion** | **New Lesion** | **Overall Response** |
| --- | --- | --- | --- |
| CR | CR | Non | CR |
| CR | Non-CR/Non-Disease Progression | Non | PR |
| CR | Not Assessable | Non | PR |
| PR | Non-Progression or Not Fully Assessable | Non | PR |
| SD | Non-Progression or Not Fully Assessable | Non | SD |
| Not Fully Assessable | Non-Progression | Non | NE |
| Disease Progression | Any Situation | Yes or No | Disease Progression |
| Any Situation | Disease Progression | Yes or No | Disease Progression |
| Any Situation | Any Situation | Yes | Disease Progression |
| CR = Complete Response | PR = Partial Response | SD = Stable Disease | NE = Not Evaluated |

**Table 2 Time Point Response - Subjects with Non-Target Lesions Only**

| **Non-Target Lesion** | **New Lesion** | **Overall Response** |
| --- | --- | --- |
| CR | Non | CR |
| Non-CR or non-disease progression | Non | Non-CR or Non-Disease Progression |
| Not Fully Assessable | Non | Not Assessable |
| Unclear Disease Progression | Yes or No | Disease Progression |
| Any Situation | Yes | Disease Progression |

Note: For non-target lesions, 'Non-CR/Non-Disease Progression' refers to efficacy better than SD. As SD is increasingly used as an endpoint for evaluating efficacy, the efficacy of Non-CR/Non-Disease Progression has been established to address situations where no measurable lesions are defined.

For unclear progression findings (such as very small uncertain new lesions; Treatment for cystic changes or necrotic lesions of the existing lesions can continue until the next evaluation. If disease progression is confirmed at the next evaluation, the date of progression should be the date when suspected progression first occurred.

**Table 3: Best overall response requiring confirmation for CR and PR efficacy**

| **First time point overall response** | **Subsequent time point overall response** | **Best overall response** |
| --- | --- | --- |
| CR | CR | CR |
| CR | PR | SD, disease progression, or PR ^a^ |
| CR | SD | If SD persists for a sufficient duration, it is considered SD; otherwise, it should be classified as disease progression. |
| CR | Disease Progression | If SD persists for a sufficient duration, it is considered SD; otherwise, it should be classified as disease progression. |
| CR | NE | If SD persists for a sufficient duration, it is considered SD; otherwise, it should be classified as NE. |
| PR | CR | PR |
| PR | PR | PR |
| PR | SD | SD |
| PR | Disease Progression | If SD persists for a sufficient duration, it is considered SD; otherwise, it should be classified as disease progression. |
| PR | NE | If SD persists for a sufficient duration, it is considered SD; otherwise, it should be classified as NE. |
| NE | NE | NE |

Note: CR = Complete Response, PR = Partial Response, SD = Stable Disease; NE = Not Evaluated.

Superscript 'a': If a CR truly occurs at the first time point, any disease that appears at subsequent time points will result in the efficacy evaluation of that subject being classified as PD at later time points, even if the subject's efficacy reaches the PR standard relative to baseline (because the disease will reappear after CR). The best response depends on whether SD occurs within the shortest treatment interval. However, sometimes the first evaluation is CR, but subsequent scans suggest that small lesions still seem to appear, thus the actual efficacy of the subject at the first time point should be PR rather than CR. In this case, the initial CR judgment should be modified to PR, and the best response is PR.

**Frequency of tumor re-evaluation at 4.5**

The frequency of tumor re-evaluation during treatment is determined by the treatment regimen and should align with the type and schedule of the treatment. However, in Phase II trials where the benefit of treatment is unclear, it is reasonable to conduct follow-ups every 6 to 8 weeks (timed at the end point of a cycle), with adjustments to the interval length possible in special protocols or circumstances. The protocol should specifically indicate which tissue sites require baseline level assessments (typically those most likely to be closely related to metastatic lesions of the tumor type under investigation) and the frequency of repeated evaluations. Under normal circumstances, both target lesions and non-target lesions should be evaluated at each assessment. In certain selectable situations, the evaluation frequency of some non-target lesions may be reduced, for example, a repeat bone scan is only required when the efficacy evaluation of the target disease confirms CR or there is suspicion of progression in bone lesions.

The re-evaluation of the tumor after treatment depends on whether the response rate or the time to the occurrence of a specific event (progression/death) is used as the endpoint of the clinical trial. If the endpoint is the time to the occurrence of a specific event (e.g., TTP/DFS/PFS), then routine repeated evaluations as specified in the protocol are required. Especially in randomized comparative trials, the predetermined evaluations should be listed in the timetable (e.g., 6 to 8 weeks during treatment, or 3 to 4 months after treatment) and should not be influenced by other factors, such as treatment delays, administration intervals, and any other events that may lead to an imbalance in treatment arms regarding the timing of disease evaluation.

**4.6 Efficacy Assessment/Confirmation of Remission**

**4.6.1 Confirmation**

For non-randomized clinical studies with efficacy as the primary endpoint, the efficacy of PR and CR must be confirmed to ensure that the efficacy is not the result of evaluation error. This also allows for a reasonable interpretation of the results in the presence of historical data; however, the efficacy in the historical data of these trials should also be confirmed. However, in all other cases, such as randomized trials (Phase II or III) or studies with disease stability or disease progression as the primary endpoints, efficacy confirmation is no longer required, as it does not add value to the interpretation of trial results. Nevertheless, the removal of the requirement for efficacy confirmation makes central review to prevent bias even more important, especially in non-blinded experimental studies.

In the case of SD, there must be at least one measurement that meets the SD criteria specified in the protocol within the shortest time interval after the trial begins (generally no less than 6 to 8 weeks).

**4.6.2 Overall Response Duration**

Overall response duration is defined as the time from the first measurement that meets the criteria for complete response (CR) or partial response (PR), whichever is measured first, to the time of the first documented disease recurrence or progression (using the minimum recorded measurement in the trial as a reference for disease progression). Total complete response duration is defined as the time from the first measurement that meets the criteria for complete response (CR) to the time of the first documented disease recurrence or progression.

**4.6.3 Disease Stabilization Duration**

This is the time from the start of treatment to the time of disease progression (in randomized trials, this starts from the time of randomization), using the minimum total sum recorded in the trial as a reference (if the baseline total sum is the minimum, it is used as the reference for calculating disease progression). The clinical relevance of disease stability varies across different studies and diseases. If the proportion of patients maintaining the shortest duration of stable disease is used as an endpoint in a specific trial, the protocol should explicitly state the minimum time interval between the two measurements in the definition of SD.

Note: The duration of response, stable disease, and PFS are influenced by the frequency of follow-up evaluations after baseline assessment. The definition of standard follow-up frequency is beyond the scope of this guideline. Follow-up frequency should consider various factors, such as disease type and stage, treatment cycles, and standard protocols. However, if comparisons between trials are necessary, the limitations of the accuracy of these measurement endpoints should be considered.

**4.7PFS/TTP**

**4.7.1 Phase II Clinical Trials**

This guideline primarily focuses on the application of objective response as a study endpoint in Phase II clinical trials. In certain cases, the response rate may not be the optimal choice for evaluating the potential anticancer activity of a new drug/new regimen. In these cases, progression-free survival (PFS) or post-progression free survival (PPF) at the defined time point may be considered appropriate alternative indicators that provide the initial signal of the biological activity of the new drug. However, it is evident that in a non-controlled trial, these evaluations may be questioned, as seemingly valuable observations may relate to biological factors such as patient selection rather than the effect of the drug intervention. Therefore, a phase II clinical trial designed as a randomized controlled trial is best with these as study endpoints. However, the clinical presentation of certain tumors remains consistent (usually in poor condition), making non-randomized trials reasonable. In these cases, however, caution must be exercised in recording efficacy evidence when assessing expected PFS or PPF due to the lack of a positive control.

**Subsequent content includes phase III evaluation endpoints, independent assessments, result reporting, etc.; please refer to the English version for details.**

## Appendix III: Modified Criteria for Evaluation of Solid Tumor Efficacy

Since there is currently no official Chinese version of mRECIST, this is an internal translation; please refer to the English version for details.

Sections 1 and 2 mainly introduce the background knowledge (see the English version for details)

3. Measurability of the tumor at baseline level

3.1 Definition

At baseline level, tumor lesions/lymph nodes will be classified into measurable and non-measurable categories as defined below:

3.1.1 Measurable Lesions

Tumor lesions: At least one dimension that can be accurately measured (recorded as the maximum diameter), with a minimum length as follows:

- CT scan 10 mm (CT scan slice thickness not greater than 5 mm)
- Clinical routine examination instruments 10 mm (tumor lesions that cannot be accurately measured with measuring instruments should be recorded as non-measurable)
- Chest X-ray 20 mm
- Malignant lymph nodes: pathologically enlarged and measurable, a single lymph node's short diameter on CT scan must be ≥15 mm (recommended CT scan slice thickness not exceeding 5 mm). Only measure and follow up on the short diameter at baseline and during follow-up.

3.1.2 Unmeasurable lesions

All other lesions, including small lesions (longest diameter <10 mm or pathological lymph node short diameter ≥10 mm to <15 mm) and lesions that cannot be measured. Unmeasurable lesions include: meningeal disease, ascites, pleural or pericardial effusion, inflammatory breast cancer, cancerous lymphangitis of the skin/lung, abdominal masses that cannot be diagnosed and followed up by imaging, and cystic lesions.

3.1.3 Special Considerations Regarding Lesion Measurement

Bone lesions, cystic lesions, and lesions that have previously undergone local treatment need to be specifically noted:

Bone Lesions:

- Bone scans, PET scans, or plain films are not suitable for measuring bone lesions, but can be used to confirm the presence or disappearance of bone lesions;
- Lytic lesions or mixed lytic/sclerotic lesions with defined soft tissue components, and where the soft tissue components meet the aforementioned criteria for measurability, can be considered measurable lesions if these lesions can be evaluated using imaging techniques such as CT or MRI;
- Osteolytic lesions are classified as non-measurable lesions.

Cystic lesions:

- - - - Lesions that meet the definition criteria for a simple cyst in radiological imaging should not be considered malignant lesions solely due to their classification as simple cysts; they are neither measurable lesions nor non-measurable lesions.
      - If the lesions are cystic metastatic lesions and meet the aforementioned criteria for measurability, they can be considered measurable lesions. However, if there are non-cystic lesions present in the same patient, non-cystic lesions should be prioritized as target lesions.

Lesions that have undergone local treatment:

- - - - Lesions located in areas previously treated with radiotherapy or other local regional therapies are generally considered non-measurable lesions, unless there is clear progression of the lesion. The study protocol should detail the conditions under which these lesions are classified as measurable.

3.2 Measurement Method Description

3.2.1 Lesion Measurement

All tumor measurements in clinical evaluations must be recorded in metric units. All baseline assessments of tumor lesion size should be completed as close to the start of treatment as possible, and must be completed within 28 days (4 weeks) prior to the start of treatment.

3.2.2 Evaluation Methods

The same techniques and methods should be used for baseline assessment and subsequent measurements of the lesions. All lesions must be evaluated using imaging examinations, except for those lesions that can only be assessed through clinical examination.

Clinical lesions: Clinical lesions can only be considered measurable if they are superficial and have a diameter of ≥10 mm at the time of measurement (e.g., skin nodules). For patients with skin lesions, it is recommended to archive color photographs that include a ruler to measure the size of the lesions. When evaluating lesions using both imaging and clinical examinations, imaging should be preferred whenever possible, as it is more objective and can be reviewed repeatedly at the end of the study.

Chest X-ray: When tumor progression is an important study endpoint, chest CT should be prioritized, as CT is more sensitive than X-ray, especially for newly developed lesions. Chest X-ray detection is only applicable when the boundaries of the measured lesions are clear and lung ventilation is good.

CT, MRI: CT is currently the best available and repeatable method for efficacy evaluation. The definition of measurability in this guideline is based on CT scan slice thickness ≤ 5 mm. If the CT slice thickness is greater than 5 mm, the minimum lesion measurement should be at least twice the slice thickness. MRI may also be acceptable in certain cases (e.g., whole-body scans).

Ultrasound: Ultrasound should not be used as a measurement method for assessing lesion size. Due to its operator dependence, ultrasound lacks reproducibility after measurements are completed and cannot ensure consistency between different measurements in technique and measurement. If new lesions are discovered using ultrasound during the trial, confirmation should be done using CT or MRI. If considering the radiation exposure from CT, MRI may be used as an alternative.

Endoscopy and laparoscopy: These techniques are not recommended for objective evaluation of tumors, but they can be used to confirm complete response (CR) when obtaining biopsy specimens, and may be used to confirm recurrence in trials where the endpoint is CR followed by recurrence or surgical resection.

Tumor markers: Tumor markers cannot be used alone to evaluate objective tumor response. However, if the marker levels exceed the upper limit of normal at baseline, they must return to normal levels when evaluating complete response. Because tumor markers vary by disease, this factor must be considered when writing measurement standards into the protocol. Specific criteria for CA-125 response (recurrent ovarian cancer) and PSA (recurrent prostate cancer) response have been published. Additionally, the International Gynecologic Cancer Society has established CA-125 progression criteria, which will be incorporated into the objective tumor response criteria for first-line treatment of ovarian cancer.

Cytological/histological techniques: These techniques may be used to identify PR and CR under specific circumstances as outlined in the protocol (for example, residual benign tumor tissue is often present in lesions of germ cell tumors). When exudate may be a potential adverse reaction to a certain therapy (such as treatment with taxane compounds or angiogenesis inhibitors), and measurable tumors meet the criteria for response or disease stability, the occurrence or worsening of tumor-related exudate during treatment can be diagnosed using cytological techniques to differentiate between response (or disease stability) and disease progression.

4. Tumor Response Assessment

4.1 Assessment of All Tumors and Measurable Lesions

To evaluate objective response or potential future progression, it is necessary to conduct a baseline assessment of the total tumor burden for all tumor lesions, serving as a reference for subsequent measurement results. In clinical protocols where objective response is the primary treatment endpoint, only patients with measurable lesions at baseline are eligible for inclusion. Measurable lesions are defined as the presence of at least one measurable lesion. For trials where disease progression (time to progression or degree of progression at a fixed date) is the primary treatment endpoint, the inclusion criteria must clearly specify whether it is limited to patients with measurable lesions or if patients without measurable lesions can also be included.

4.2 Baseline Recording of Target and Non-Target Lesions

When there are more than one measurable lesions at baseline assessment, all lesions should be recorded and measured, with a total not exceeding 5 (no more than 2 from each organ), representing target lesions for all affected organs (that is, patients with only one or two affected organs may select a maximum of two or four target lesions for baseline measurement).

Target lesions must be selected based on size (longest diameter), capable of representing all affected organs, and measurements must demonstrate good reproducibility. Sometimes, when the largest lesion cannot be measured reproducibly, a new largest lesion that can be measured reproducibly may be selected.

Lymph nodes require special attention as they are normal tissue and can be detected by imaging even in the absence of tumor metastasis. Defined as measurable nodules or pathological lymph nodes that must meet the following criteria: CT measurement of the short diameter ≥ 15 mm. Only the short diameter needs to be measured at baseline. Radiologists typically use the short diameter of the nodule to determine whether it has metastasized. Nodule size is generally represented using two-dimensional data from imaging (CT uses axial planes, while MRI selects one plane from axial, sagittal, or coronal views). The minimum value is considered the short diameter. For example, a 20 mm × 30 mm abdominal nodule with a short diameter of 20 mm can be considered a malignant, measurable nodule. In this example, 20 mm is the measurement value of the nodule. Nodules with a diameter ≥ 10 mm but < 15 mm should not be considered target lesions. Nodules less than 10 mm do not fall within the pathological nodule category and do not need to be recorded or further observed.

The sum of the diameters of all target lesions calculated (including the longest diameter of non-nodular lesions and the short diameter of nodular lesions) will be reported as the baseline diameter total. If lymph nodes are included, as mentioned above, only the short diameter will be counted. The baseline diameter total will serve as a reference value for the disease baseline level.

All other lesions, including pathological lymph nodes, can be considered non-target lesions and do not need to be measured, but should be recorded during the baseline assessment. Records may indicate 'present', 'missing', or in very rare cases, 'definite progression'. Widely present target lesions may be recorded together with target organs (e.g., extensive pelvic lymphadenopathy or massive liver metastasis).

4.3 Response Criteria

4.3.1 Assessment of Target Lesions

Complete Response (CR): All target lesions disappear, and all pathological lymph nodes (including target and non-target nodules) must have a short diameter reduced to <10 mm.

Partial Response (PR): The sum of the diameters of target lesions must decrease by at least 30% from baseline levels.

Progressive Disease (PD): Referring to the minimum sum of the diameters of all measured target lesions throughout the study, an increase of at least 20% in diameter and relative increase is required (if the baseline measurement is the minimum, then the baseline value is used as a reference); In addition, the absolute increase in the sum of diameters must be at least 5 mm (the appearance of one or more new lesions is also considered disease progression).

Stable Disease (SD): The degree of reduction in target lesions does not reach PR, and the degree of increase does not reach PD levels, falling between the two; during the study, the minimum sum of diameters can be used as a reference.

4.3.2 Considerations for Target Lesion Assessment

Lymph Nodes: Even if the identified target lymph nodes decrease to less than 10 mm, the actual short diameter corresponding to the baseline must still be recorded during each measurement (consistent with the anatomical plane at baseline measurement). This means that if the lymph nodes are classified as target lesions, even if they meet the criteria for complete response, it cannot be said that the lesions have completely disappeared, because the short diameter of normal lymph nodes is defined as <10 mm. In the CRF form or other recording methods, target lymph node lesions must be specifically recorded in designated locations: for CR, all lymph node short diameters must be <10 mm; For PR, SD, and PD, the actual measured short diameter of the target lymph nodes will be included in the sum of the diameters of the target lesions.

Target lesions that are too small to measure: In clinical research, all lesions (nodular or non-nodular) recorded at baseline should be re-measured in subsequent assessments, even if the lesions are very small (e.g., 2 mm). However, sometimes they may be too small, resulting in images from CT scans being very blurry, making it difficult for radiologists to define exact values, and they may report it as 'too small to measure'. In such cases, it is very important to record the previous value on the CRF form. If the radiologist believes that the lesion may have disappeared, it should also be recorded as 0 mm. If the lesion is indeed present but is somewhat vague, making it impossible to provide an accurate measurement, it can be defaulted to 5 mm. (Note: The likelihood of lymph nodes exhibiting this situation is low, as they generally have measurable sizes under normal circumstances, or are often surrounded by adipose tissue, as in the retroperitoneal cavity; However, if such a situation arises where a measurement cannot be provided, it is also defaulted to 5 mm.)The default value of 5 mm is derived from the slice thickness of the CT scan (this value does not change with different slice thickness values of the CT). Since the likelihood of the same measurement value occurring repeatedly is low, providing this default value will reduce the risk of erroneous assessments. However, it must be reiterated that if the radiologist can provide the exact size of the lesion, even if the diameter is less than 5 mm, the actual value must be recorded.

Separated or combined lesions: When non-nodular lesions fragment into pieces, the longest diameter of each separated part is summed to calculate the total diameter of the lesion. Similarly, for combined lesions, they can be distinguished through the planes between the combined parts, and then the maximum diameter of each can be calculated. However, if the combination is inseparable, the longest diameter should be taken as the longest diameter of the fused lesion as a whole.

4.3.3 Assessment of Non-target Lesions

This section defines the criteria for the response of non-target lesions. Although some non-target lesions are actually measurable, measurement is not required; qualitative assessment at the time points specified in the protocol is sufficient.

Complete Response (CR): All non-target lesions disappear, and tumor markers return to normal levels. All lymph nodes are of non-pathological size (short axis < 10 mm).

Partial Response/Non-disease Progression: One or more non-target lesions are present and/or tumor marker levels remain above normal.

Disease Progression: Existing non-target lesions show clear progression. Note: The appearance of one or more new lesions is also considered disease progression.

4.3.4 Special Considerations for the Assessment of Non-target Lesion Progression

The following is a supplementary explanation regarding the definition of progression of non-target lesions: When a patient has measurable non-target lesions, even if the target lesions are assessed as stable or partially responding, a clear definition of progression based on the non-target lesions must meet the criterion that the overall deterioration of the non-target lesions has reached a level that necessitates the termination of treatment. Moreover, a general increase in the size of one or more non-target lesions is often insufficient to meet the criteria for progression; therefore, in cases where the target lesions are stable or partially responding, it is exceedingly rare to define overall tumor progression solely based on changes in non-target lesions.

This situation may occur in some Phase III trials when the inclusion criteria do not specify that measurable lesions must be present, particularly when all non-target lesions in the patient are unmeasurable. Overall assessment still refers to the above standards, but there are no measurable data for lesions in this situation. The deterioration of non-target lesions is not easy to assess (by definition: all non-target lesions must indeed be unmeasurable). Therefore, when changes in non-target lesions lead to an increase in overall disease burden equivalent to the progression of target lesions, a clear definition of progression based on non-target lesions requires the establishment of an effective detection method for evaluation. For example, an increase in tumor burden is described as a volume increase of 73% (equivalent to a 20% increase in the diameter of measurable lesions). Additionally, peritoneal effusion may range from 'trace' to 'massive'; lymphatic involvement may progress from 'localized' to 'widely disseminated'; or it may be described in the protocol as 'sufficient to necessitate a change in treatment approach.'Examples include pleural effusion ranging from trace to massive, lymphatic involvement spreading from the primary site to distant sites, or it may be described in the protocol as 'necessitating a change in treatment.'If clear progression is observed, the patient should be considered to have disease progression at that point overall. It is best to have objective criteria applicable to the assessment of unmeasurable lesions; note that the additional criteria must be reliable.

4.3.5 New Lesions

The emergence of new malignant lesions indicates disease progression; therefore, some evaluations targeting new lesions are very important. Currently, there are no specific criteria for imaging detection of lesions; however, the discovery of a new lesion should be clear. For example, progression cannot be attributed to differences in imaging techniques, changes in imaging morphology, or other lesions outside the tumor (e.g., some so-called new bone lesions are merely the resolution of the original lesion or a recurrence of the original lesion). It is very important when the patient's baseline lesions show partial or complete response; for example, necrosis of a liver lesion may be classified as a new cystic lesion in the CT report, when in fact it is not.

Lesions detected during follow-up that were not found in the baseline examination will be considered new lesions and indicate disease progression. For example, in a patient with visceral lesions found during the baseline examination, if metastatic lesions are discovered during a CT or MRI of the head, the patient's intracranial metastatic lesions will be regarded as evidence of disease progression, even if a head examination was not performed during the baseline assessment.

If a new lesion is unclear, for example due to its small size, further treatment and follow-up evaluation are required to confirm whether it is a new lesion. If repeated examinations confirm it as a new lesion, the time of disease progression should be calculated from the time of its initial discovery.

Lesions undergoing FDG-PET assessment generally require additional tests for supplementary confirmation; it is reasonable to evaluate progression by combining the results of FDG-PET and supplementary CT examinations (especially for new suspicious diseases). New lesions can be clarified through FDG-PET examination, executed according to the following procedure:

The baseline FDG-PET scan result is negative, and the subsequent follow-up FDG-PET scan is positive, indicating disease progression.

No baseline FDG-PET scan was performed, and the results of the subsequent FDG-PET scan are positive:

If the new lesions found in the follow-up FDG-PET positive scan correspond with the results from the CT scan, it confirms disease progression.

If the new lesions found in the follow-up FDG-PET positive scan are not confirmed by the CT scan results, a repeat CT scan is required for confirmation (if confirmed, the time of disease progression is calculated from the initial abnormal FDG-PET scan).

If the positive results of the follow-up FDG-PET scan correspond with the lesions already present on the CT scan, and there is no progression of the lesions on imaging, then the disease is considered stable.

4.4 Best Overall Response Evaluation

The evaluation of best overall efficacy is the best efficacy record from the start of the trial to its conclusion, while considering any necessary conditions for confirmation. Sometimes, efficacy responses occur after the end of treatment; therefore, the protocol should clarify whether efficacy evaluations after treatment are included in the best overall efficacy evaluation. The protocol must specify how any new treatment prior to progression affects the best efficacy response. The best efficacy response in patients primarily depends on the results of target lesions, non-target lesions, and the performance of new lesions. Additionally, it relies on the nature of the trial, protocol requirements, and outcome measurement standards. Specifically, in non-randomized trials, the efficacy response is the primary objective, and confirmation of efficacy through PR or CR is necessary to determine which represents the best overall efficacy response.

4.4.1 Timepoint Response

It is assumed that efficacy responses will occur at specific time points for each protocol. Table 1 will provide a summary of the overall response of patients with measurable disease at baseline at each timepoint.

If the patient has no measurable lesions (no target lesions), the assessment can be referenced in Table 2.

4.4.2 Explanation of Missing and Non-evaluable Assessments

If imaging or measurement of lesions cannot be performed at a specific time point, the patient cannot be evaluated at that time point. If only a portion of the lesions can be evaluated in an assessment, this situation is generally considered non-evaluable at that time point, unless there is evidence to confirm that the missing lesions will not affect the efficacy response evaluation at the specified time point. This situation is likely to occur in cases of disease progression. For example: a patient has a total of 3 lesions measuring 50 mm at baseline, but subsequently only 2 lesions are evaluable, totaling 80 mm; the patient will be assessed as having disease progression, regardless of the impact of the missing lesions.

4.4.3 Best Overall Response: All Time Points

Once all patient data is available, the best overall response can be determined.

Assessment of best overall response when confirmation of complete or partial efficacy response is not required: The best efficacy response in the trial is the best response at all time points (for example: a patient is evaluated as SD in the first cycle, PR in the second cycle, and PD in the last cycle, but their best overall response is evaluated as PR). When the best overall response is evaluated as SD, it must meet the minimum duration specified in the protocol from baseline. If the minimum duration criteria are not met, even if the best overall response is rated as SD, it will not be recognized; the patient's best overall response will depend on subsequent evaluations. For example: a patient is evaluated as SD in the first cycle and PD in the second cycle, but they did not meet the minimum time requirement for SD, their best overall response is evaluated as PD. The same patient who is rated as SD in the first cycle and then lost to follow-up will be considered unassessable.

Assessment of best overall response when confirmation of complete or partial efficacy response is required: It can only be declared as complete or partial response if each subject meets the criteria for partial or complete response specified in the trial and efficacy confirmation is performed at subsequent time points (generally four weeks later) as specifically mentioned in the protocol. In this case, the best overall response is described in Table 2.

4.4.4 Special Notes on Efficacy Assessment

When nodular lesions are included in the overall target lesion assessment, and the size of the nodules reduces to 'normal' size (<10 mm), they will still have a lesion size scan report. To avoid overestimating the situation reflected by the increase in lesion size, even if the lesion is normal, the measurement results will still be recorded. As previously mentioned, this means that for subjects with a complete response, the CRF will not record a value of 0.

If efficacy confirmation is required during the trial, repeated 'non-measurable' time points will complicate the assessment of the best efficacy. The analysis plan of the trial must clarify how these missing data/evaluations can be interpreted when determining efficacy. For example, in most trials, a subject's response of PR-NE-PR can be considered as confirmation of efficacy.

When a subject experiences an overall deterioration in health that necessitates stopping treatment, but there is no objective evidence to support this, it should be reported as symptomatic progression. Efforts should still be made to assess objective progression even after treatment has been discontinued. Symptomatic deterioration is not a description of objective response assessment: it is the reason for stopping treatment. The objective response of such subjects will be assessed based on the status of target and non-target lesions as shown in Tables 1 to 3.

Conditions defined as early progression, early death, and unassessable situations are exceptions in the study and should be clearly described in each protocol (depending on the treatment interval and treatment cycle).

In some cases, it is difficult to distinguish local lesions from normal tissue. When the assessment of complete response is based on such definitions, we recommend performing a biopsy prior to the efficacy evaluation of complete response in local lesions. When some subjects have abnormal imaging results of local lesions that are considered indicative of lesion fibrosis or scar formation, FDG-PET is used as an assessment standard similar to biopsy for confirming complete response. In this case, the application of FDG-PET should be prospectively described in the protocol, supported by reports from specialized medical literature addressing this situation. However, it must be recognized that the inherent limitations of both FDG-PET and biopsy (including the varying resolutions and sensitivities of the two) will lead to false positive results in the assessment of complete response.

Table 1 Response at Time Points: Subjects with Target Lesions (including or excluding non-target lesions)

| Target Lesion | Non-Target Lesion | New Lesion | Overall Response |
| --- | --- | --- | --- |
| CR | CR | Non | CR |
| CR | Non-CR/Non-PD | Non | PR |
| CR | Not Assessable | Non | PR |
| PR | Non-Progression or Not Fully Assessable | Non | PR |
| SD | Non-Progression or Not Fully Assessable | Non | SD |
| Not Fully Assessable | Non-Progression | Non | NE |
| PD | Any Situation | Yes or No | PD |
| Any Situation | PD | Yes or No | PD |
| Any Situation | Any Situation | Yes | PD |
| CR = Complete Response | PR = Partial Response | SD = Stable Disease | PD = Progressive Disease  NE = Not Evaluated |

Table 2 Response at Time Points - Subjects with Only Non-Target Lesions

| Non-Target Lesion | New Lesion | Overall Response |
| --- | --- | --- |
| CR | Non | CR |
| Non-CR or non-PD | Non | Non-CR or Non-PD |
| Not Fully Assessable | Non | Not Assessable |
| Unclear PD | Yes or No | PD |
| Any Situation | Yes | PD |

Note: For non-target lesions, 'Non-CR/Non-PD' refers to efficacy superior to SD. As SD is increasingly used as an endpoint for evaluating efficacy, the definition of Non-CR/Non-PD efficacy is established to address situations where no measurable lesions are specified.

For unclear progression findings (such as very small uncertain new lesions; Treatment for cystic changes or necrotic lesions of the existing lesions can continue until the next evaluation. If disease progression is confirmed at the next evaluation, the date of progression should be the date when suspected progression first occurred.

Table 2 Best Overall Response Requiring Confirmation for CR and PR

| First time point overall response | Subsequent time point overall response | Best overall response |
| --- | --- | --- |
| CR | CR | CR |
| CR | PR | SD, PD, or PR ^a^ |
| CR | SD | If SD persists for a sufficient duration, it is classified as SD; otherwise, it should be classified as PD. |
| CR | PD | If SD persists for a sufficient duration, it is classified as SD; otherwise, it should be classified as PD. |
| CR | NE | If SD persists for a sufficient duration, it is considered SD; otherwise, it should be classified as NE. |
| PR | CR | PR |
| PR | PR | PR |
| PR | SD | SD |
| PR | PD | If SD persists for a sufficient duration, it is classified as SD; otherwise, it should be classified as PD. |
| PR | NE | If SD persists for a sufficient duration, it is considered SD; otherwise, it should be classified as NE. |
| NE | NE | NE |

Note: CR stands for complete response, PR stands for partial response, SD stands for stable disease, PD stands for progressive disease, and NE stands for not evaluable. Superscript 'a': If a CR truly occurs at the first time point, any disease that appears at subsequent time points will result in the efficacy evaluation of that subject being classified as PD at later time points, even if the subject's efficacy reaches the PR standard relative to baseline (because the disease will reappear after CR). The best response depends on whether SD occurs within the shortest treatment interval. However, sometimes the first evaluation is CR, but subsequent scans suggest that small lesions still seem to appear, thus the actual efficacy of the subject at the first time point should be PR rather than CR. In this case, the initial CR judgment should be modified to PR, and the best response is PR.

4.5. Frequency of Tumor Reevaluation

The frequency of tumor re-evaluation during treatment is determined by the treatment regimen and should align with the type and schedule of the treatment. However, in phase II trials where the benefit of treatment is unclear, it is reasonable to conduct follow-ups every 6 to 8 weeks (timed at the end of a cycle), with adjustments to the interval length possible in special protocols or circumstances. The protocol should specifically indicate which tissue sites require baseline level assessments (typically those most likely to be closely related to the metastatic lesions of the tumor type under investigation) and the frequency of evaluations. Under normal circumstances, both target lesions and non-target lesions should be evaluated at each assessment. In certain selectable situations, the evaluation frequency of some non-target lesions may be reduced, for example, a repeat bone scan is only required when the efficacy evaluation of the target disease confirms CR or there is suspicion of progression in bone lesions.

The re-evaluation of the tumor after treatment depends on whether the response rate or the time to the occurrence of a specific event (progression/death) is used as the endpoint of the clinical trial. If the endpoint is the time to the occurrence of a specific event (e.g., TTP/DFS/PFS), then routine repeated evaluations as specified in the protocol are required. Especially in randomized comparative trials, the predetermined evaluations should be listed in the timetable (e.g., 6 to 8 weeks during treatment, or 3 to 4 months after treatment) and should not be influenced by other factors, such as treatment delays, administration intervals, and any other events that may lead to an imbalance in treatment arms regarding the timing of disease evaluation.

4.6 Efficacy Assessment/Confirmation of Remission

4.6.1 Confirmation

For non-randomized clinical studies with efficacy as the primary endpoint, the efficacy of PR and CR must be confirmed to ensure that the efficacy is not the result of evaluation error. This also allows for reasonable interpretation of results in the presence of historical data; however, the efficacy in the historical data of these trials should also be confirmed. However, in all other cases, such as randomized trials (Phase II or III) or studies with disease stability or disease progression as the primary endpoints, efficacy confirmation is no longer required, as it holds no value for the interpretation of trial results. Nevertheless, the removal of the requirement for efficacy confirmation makes central review to prevent bias even more important, especially in non-blinded experimental studies.

In the case of SD, there must be at least one measurement that meets the SD criteria specified in the protocol within the shortest time interval after the trial begins (generally no less than 6 to 8 weeks).

4.6.2 Overall Remission Period

Overall response duration is defined as the time from the first measurement that meets the criteria for complete response (CR) or partial response (PR), whichever is measured first, to the time of the first documented disease recurrence or progression (using the minimum recorded measurement in the trial as a reference for disease progression). Total complete response duration is defined as the time from the first measurement that meets the criteria for complete response (CR) to the time of the first documented disease recurrence or progression.

4.6.3 Disease Stability Period

It is the time from the start of treatment to disease progression (in randomized trials, from the time of random assignment), using the minimum total in the trial as a reference (if the baseline total is the minimum, it is used as a reference for PD calculation). The clinical relevance of disease stability varies across different studies and diseases. If the proportion of patients maintaining the shortest duration of stable disease is used as an endpoint in a specific trial, the protocol should explicitly state the minimum time interval between the two measurements in the definition of SD.

Note: The duration of response, stable disease, and PFS are influenced by the frequency of follow-up evaluations after baseline assessment. The definition of standard follow-up frequency is beyond the scope of this guideline. Follow-up frequency should consider various factors, such as disease type and stage, treatment cycles, and standard protocols. However, if comparisons between trials are necessary, the limitations of the accuracy of these measurement endpoints should be considered.

The content from 4.7 onwards serves as endpoint indicators, independent evaluations, and result reports, please refer to the English version for details.

**Appendix Four: Child-Pugh Classification Standards for Liver Function**

[The compensated and decompensated stages of cirrhosis provide a rough estimate of liver function in patients with cirrhosis; the boundary between the two stages is difficult to delineate, and there is significant variability in the severity of illness among patients in the decompensated stage. Child (1964) classified the varying degrees of five indicators: serum bilirubin, ascites, serum albumin concentration, prothrombin time, and general condition into three levels (1, 2, 3) for scoring. The minimum score for the five indicators is 5 points, and the maximum score is 15 points. Based on the score, patients are classified into three categories: A, B, and C. Due to the difficulty in scoring general condition, Pugh subsequently replaced the presence and severity of hepatic encephalopathy with general condition, which is known as the Child-Pugh modification grading system. The Child-Pugh modification grading system is divided into three levels: Class A scores 5-6 points, indicating low surgical risk; Class B scores 7-9 points, indicating moderate surgical risk; Class C scores 10-15 points, indicating high surgical risk.](http://www.haodf.com/jibing/ganyinghua.htm)

**Child-Pugh scoring and grading of liver disease severity**

| **Indicators** | **Abnormality degree scoring** | | |
| --- | --- | --- | --- |
|  | **1** | **2** | **3** |
| Hepatic encephalopathy | None | 1～2 | 3～4 |
| Ascites | None | Mild | Moderate or above |
| Serum total bilirubin (μmol/L) | ＜34.2 | 34.2～51.3 | ＞51.3 |
| Serum Albumin (g/L) | ≥35 | 28～34 | ＜28 |
| Prothrombin Time (seconds) | ≤14 | 15～17 | ≥18 |

**Appendix Five Eastern Oncology Cooperative Group (ECOG) Performance Status Scale**

| **Grade** | **Standard** |
| --- | --- |
| 0 | Completely normal, able to perform all normal activities without restriction (Karnofsky 90-100) |
| 1 | Unable to engage in strenuous physical activity, but can walk and perform light physical activities or office work (Karnofsky 70-80) |
| 2 | Can walk, self-care is possible, but unable to work; bed rest during the day does not exceed 50% (Karnofsky 50-60) |
| 3 | Self-care is barely possible; requires bed rest or sitting in a chair for more than 50% of the day (Karnofsky 30-40) |
| 4 | Complete loss of mobility, severe inability to care for oneself, must be bedridden or use a wheelchair (Karnofsky 10-20) |

**Appendix Six Portal Vein Tumor Thrombus (PVTT) Classification**

According to Cheng's classification, PVTT is divided based on the extent of invasion into the portal vein:

I _0_ Type: Microscopic portal vein microthrombus;

Type I, thrombus involving secondary and higher-order portal vein branches (lobe or segment branches);

Type II, thrombus involving primary portal vein branches (left or right branch);

Type III, thrombus involving the main trunk of the portal vein;

Type IV, with tumor thrombus involving the superior mesenteric vein.

**Appendix Seven: Management Measures for Allergic Reactions**

**Required Equipment**

- Tourniquet ;
- Oxygen ;
- Epinephrine for subcutaneous injection, intravenous injection, and/or endotracheal use according to standard diagnostic and therapeutic protocols ;
- Antihistamines ;
- Corticosteroids ;
- Intravenous fluids, tubing, catheters, and tape.

**Procedure**

In the event of suspected allergic reactions during the infusion of the investigational drug, the following procedures should be implemented:

- 1. Stop the infusion of the investigational drug ;
  2. Use a tourniquet at the proximal injection site to slow the systemic absorption of the investigational drug. Do not occlude arterial blood flow to the affected limb ;
  3. Maintain airway patency ;
  4. Administer antihistamines, epinephrine, or other medications as per the patient's condition under the guidance of the on-duty physician, continue to monitor the patient and record the observations.

**Appendix Eight Recommendations for Replacement Therapy in Hypothyroidism**

Patients who develop treatment-related hypothyroidism are recommended to use levothyroxine sodium tablets (commonly known by the brand name Euthyrox).

The doses recommended in this appendix are general principles; the individual daily dose for patients should be determined based on laboratory tests and clinical examination results. Due to the elevation of total thyroxine (T4) and FT4 levels in many patients, the baseline concentration of serum thyroid-stimulating hormone is a reliable basis for determining the treatment method. General thyroid hormone treatment should start with a low dose and gradually increase every 2 to 4 weeks until a sufficient dose is reached. Generally, patients who develop hypothyroidism require lifelong medication.

For elderly patients, patients with coronary heart disease, and those with severe or long-term hypothyroidism, special attention should be paid during the initiation phase of thyroid hormone therapy. A lower initial dose (e.g., 12.5 μg/day) should be selected, and the dosage should be gradually increased over a longer time interval (e.g., increasing by 12.5 μg/day every two weeks).

If the patient's final maintenance dose is below the optimal dose, it may not fully correct their TSH levels.

Experience shows that low-dose administration is effective for lighter-weight patients as well as those with large nodular goiter.

Levothyroxine sodium tablets should be taken with an appropriate liquid (e.g., half a cup of water) half an hour before breakfast, on an empty stomach, in a single daily dose.

**Table 1 Recommended Dosage Chart**

|  | **dose** | **50 μg tablet** | **100 μg tablet** | **Method of administration** |
| --- | --- | --- | --- | --- |
| Initial dose  (Increase by 25–50 μg every 2–4 weeks after the initial dose, until the maintenance dose is reached) | 25～50 μg | 1/2 to 1 tablet | 1/4 to 1/2 tablet | Once daily |
| Maintenance dose | 100～200 μg | 2 to 4 tablets | 1 to 2 tablets | Once daily |

For secondary hypothyroidism, the cause must be determined before initiating replacement therapy with this product, and glucocorticoid supplementation should be provided if necessary. Once the treatment with levothyroxine is established. In the case of changing medications, it is recommended to adjust the dose based on the patient's clinical response and the results of laboratory tests.

For the rest, please refer to the relevant product instructions.
